# DBU-Promoted Cascade Cyclization/Thiolation of β‑Enamino Diketones to 5‑Sulfenylated 3‑Hydroxy-γ-lactams

**DOI:** 10.1021/acs.joc.6c00140

**Published:** 2026-05-04

**Authors:** Julia Poletto, Julia C. M. Willig, Jeniffer N. A. Camargo, Fernanda A. Rosa

**Affiliations:** Laboratory for Synthesis of Heterocycles (SINTHET), Chemistry Department, State University of Maringá - UEM, Maringá, Paraná 87020-900, Brazil

## Abstract

Efficient access to γ-sulfenylated 3-hydroxy-γ-lactams remains limited, because the existing multicomponent methods do not enable γ-heterofunctionalization. We report a mild DBU-promoted cyclization/thiolation cascade that converts β-enamino diketones and thiols into 5-sulfenylated γ-lactams in up to 98% yield with a broad *N*-aryl/*N*-alkyl scope and diverse sulfur nucleophiles. Control and in situ NMR experiments supported a nucleophile-initiated mechanism, providing a streamlined route to functionally rich γ-lactam scaffolds for heterocycles and medicinal chemistry.

## Graphical abstract

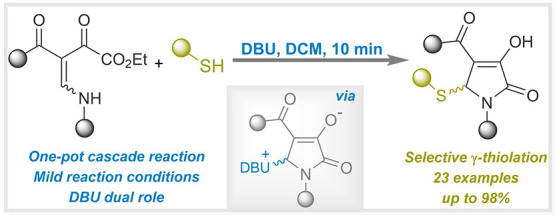

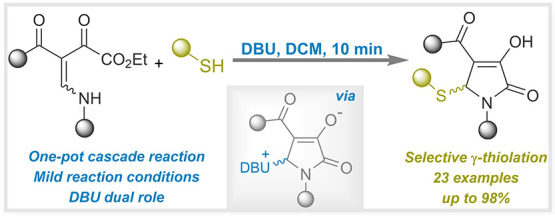

## Introduction

Synthesis of structurally diverse and functionally enriched γ-lactam scaffolds remains a central goal in heterocyclic chemistry. Among these, 1,5-dihydro-2*H*-pyrrol-2-ones have garnered significant attention because of their synthetic versatility and biological relevance, serving both as intermediates in organic synthesis and as core structures in bioactive natural products.^1^ Within this family, 3-hydroxy-1,5-dihydro-2*H*-pyrrol-2-ones represent a lesser-studied subclass found in natural compounds such as phenopyrrozin and *p*-hydroxyphenopyrrozin (Scheme 1A), which possess antimicrobial properties against Gram-positive bacteria.^2^ The hydroxy group in these compounds not only enhances interactions with biological targets but also provides a valuable site for further chemical modification.^3^ Moreover, dithiolopyrrolone (DTP) natural products^4^ (Scheme 1A), including holomycin and thiolutin, exhibit broad-spectrum inhibition in bacteria, fungi, and human cell lines.

**Scheme 1:**
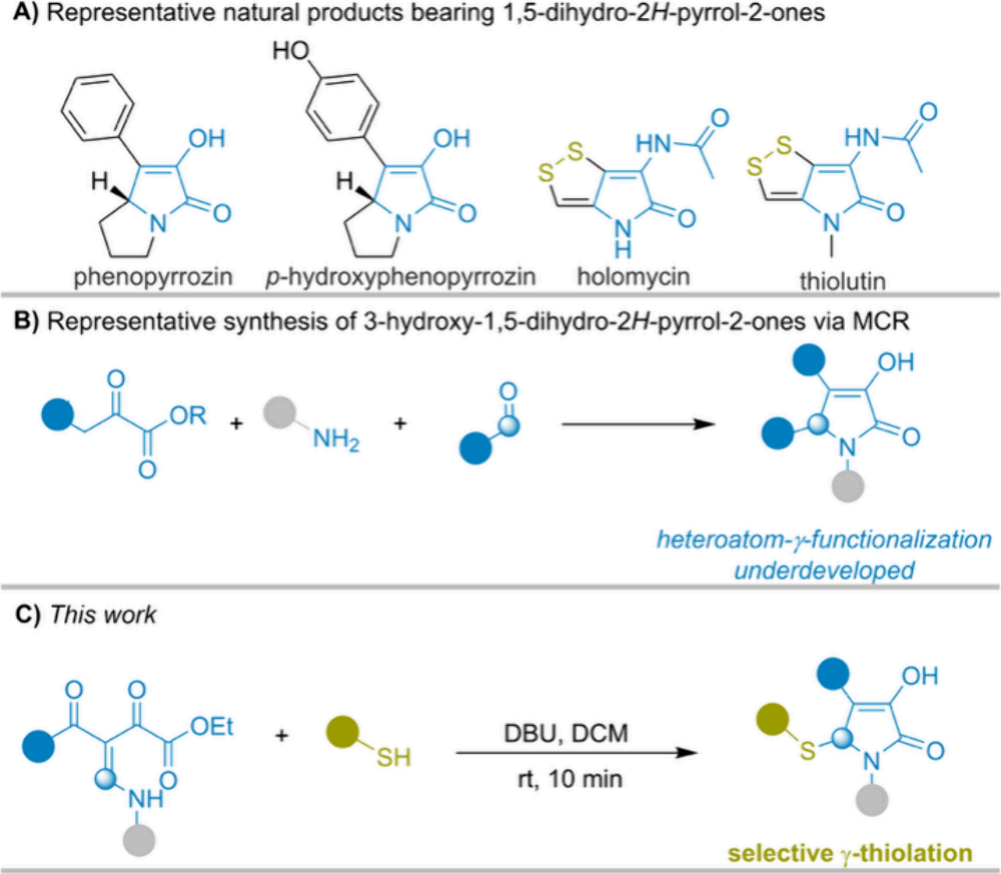
Importance of 1,5-Dihydro-2*H*-pyrrol-2-ones and Their Synthetic Limitations

To access these valuable scaffolds, multicomponent reactions involving α-oxo esters or acids, aldehydes, and amines have been developed, which highlight their efficiency and modularity.^5^ Despite their practicality, these approaches mainly furnish 5-aryl-3-hydroxy-γ-lactams and do not enable γ-heterofunctionalization, thereby limiting the scaffold diversity (Scheme 1B). This limitation is particularly notable, given the prevalence of heteroatom-containing motifs in biologically active compounds. Among these motifs, sulfur-containing fragments are especially widespread in bioactive molecules and natural products,^6^ maintaining strong interest in efficient carbon–sulfur bond-forming reactions. Nevertheless, methods that achieve γ-lactam formation and sulfur incorporation in a single operation, particularly at the γ-position, remain scarce and often lack either synthetic efficiency or broad applicability.^7^

Consequently, streamlined strategies that merge cyclization and functionalization in a single step are of considerable synthetic value.

Herein, we report a mild and operationally simple strategy for the synthesis of 5-sulfenylated 3-hydroxy-1,5-dihydro-2*H*-pyrrol-2-ones via a one-pot cyclization/thiolation cascade reaction (Scheme 1C). This transformation proceeds from β-enamino diketones, exploiting their synthetic flexibility to access a broad range of sulfenylated γ-lactams. Their inherent structural features, particularly the presence of multiple nucleophilic and electrophilic centers, render these substrates ideal precursors for simultaneous cyclization and functionalization in a single cascade.^8^ The method exhibits a broad substrate scope, excellent functional-group tolerance, and amenability for further diversification, making it a valuable addition to the synthetic toolbox for heterocyclic and medicinal chemistry.

## Results and Discussion

The initial reaction conditions were selected on the basis of our previous studies with related β-enamino diketone substrates, in which treatment with DBU and PTSA led to the formation of a 4-acyl-3,5-dihydroxy-pyrrol-2-one intermediate.^8b^ Thus, we examined a one-pot stepwise sequence involving BED **1a** and DBU, followed by the addition of ethyl thioglycolate **2a** and PTSA (Table 1). Solvent screening at ambient temperature revealed that protic solvents such as MeOH and EtOH provided higher yields of **3a** than did polar aprotic solvents such as MeCN and DCM (Table 1, entries 1–4).

**Table 1:**
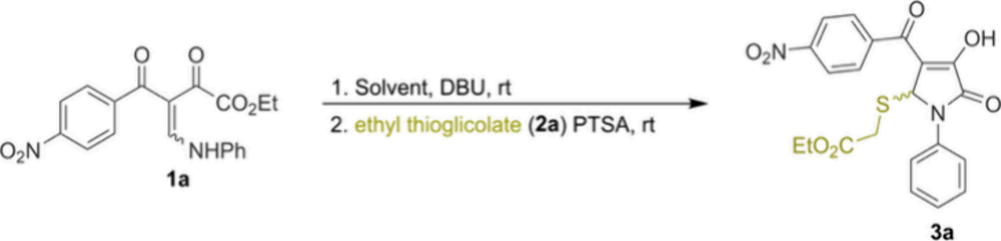
Optimization of the Reaction Conditions^a^

Entry	Solvent	Time 1 (min)	PTSA (equiv)	Time 2 (min)	Yield (%)^b^
1	DCM	3	2.2	60	37
2	MeCN	40	2.2	60	32
3	MeOH	40	2.2	60	54
4	EtOH	40	2.2	60	63
5	DCM	3	1.2	60	80
6	MeCN	40	1.2	60	75
7	MeOH	40	1.2	60	81
8	EtOH	40	1.2	60	76
9	DCM	3	–	60	84
10^c^	DCM	3	–	60	–^d^
11^e^	DCM	–	–	–	84
12^f^	DCM	–	–	–	90

a Reaction conditions: Step 1. **1a** (0.20 mmol, 1.0 equiv), DBU (0.24 mmol, 1.2 equiv), solvent (3 mL), rt, 3–40 min. Step 2. **2a** (0.22 mmol, 1.1 equiv), PTSA, rt, (10–60 min).

b Yield of the isolated product.

c DBU (0.12 mmol, 0.6 equiv).

d Recovered 40% of **1a**.

e Reaction performed in cascade: **1a** (0.20 mmol, 1.0 equiv), DBU (0.24 mmol, 1.2 equiv), **2a** (0.22 mmol, 1.1 equiv), solvent (3 mL), rt, 60 min.

f Reaction performed in cascade for 10 min.

The investigation of PTSA loading showed that 1.2 equiv afforded good results, particularly in DCM (80% yield; Table 1, entries 5–8). Surprisingly, however, the reaction in DCM also proceeded efficiently in the absence of PTSA, delivering **3a** in high yield within 10 min (Table 1, entry 9). In contrast, decreasing the DBU loading to 0.6 equiv significantly impaired the reaction, leading to incomplete conversion and recovery of 40% of starting material **1a** (Table 1, entry 10), thereby underscoring the crucial role of DBU in this transformation. These observations prompted further evaluation of the cascade protocol under the optimized conditions (Table 1, entries 10 and 11). This cascade approach delivered markedly higher efficiency and improved yields compared to the initial stepwise procedure and was therefore selected as the standard method.

With the optimized cascade conditions in hand, we explored the substrate scope for the synthesis of γ-sulfenylated 3-hydroxy-γ-lactams **3** (Scheme 2). First, we assessed the versatility of the protocol across a series of *N*-substituted β-enamino diketones. *N*-Aryl BED derivatives bearing diverse substitution patterns and electronic properties on either phenyl ring underwent smooth conversion under the standard conditions, consistently affording the corresponding γ-sulfenylated 3-hydroxy-γ-lactams **3a–3m** in excellent isolated yields (80–98%).

**Scheme 2:**
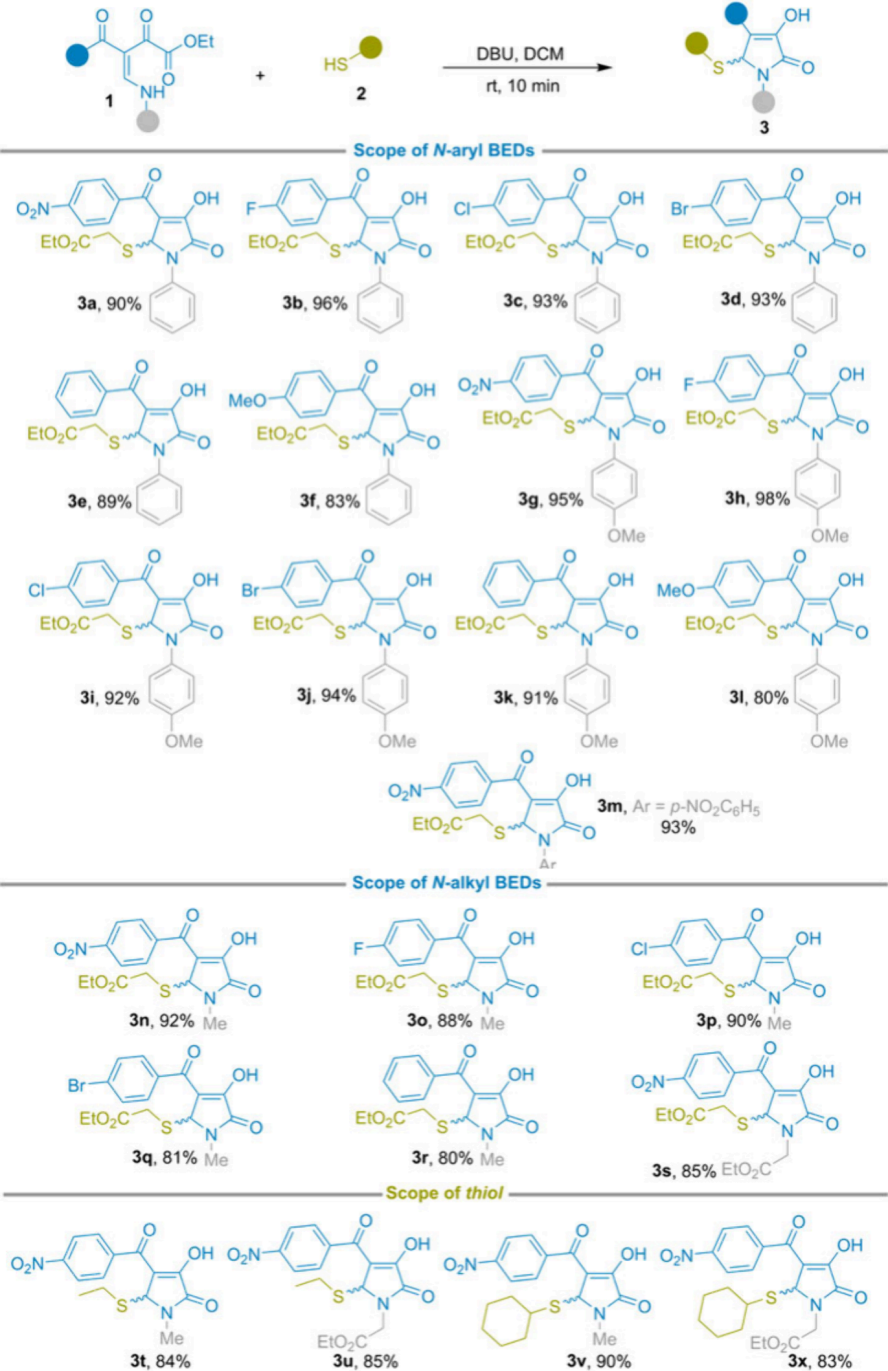
Reaction Scope^a^

a Reaction conditions: **1** (0.20 mmol), DBU (0.24 mmol), **2** (0.22 mmol), DCM (3 mL), rt, 10 min. Isolated yield

Importantly, the protocol was also effective for *N*-alkyl BED substrates, delivering products **3n–3s** in high yields, thereby demonstrating a broad tolerance with respect to *N*-substituent variation. In addition to ethyl thioglycolate, other thiol nucleophiles were evaluated under the established conditions. Both ethanethiol and cyclohexanethiol participated smoothly in the cascade, providing sulfenylated lactams **3t–3x** in good yields. These results highlight the generality of the protocol for the incorporation of diverse sulfur-containing fragments into the γ-lactam framework.

To further illustrate the feasibility of this reaction, an experiment was conducted on a gram scale. Upon increasing the reaction scale to 2.49 mmol, the desired compound **3d** was produced with an 98% yield (Scheme 3).

**Scheme 3:**
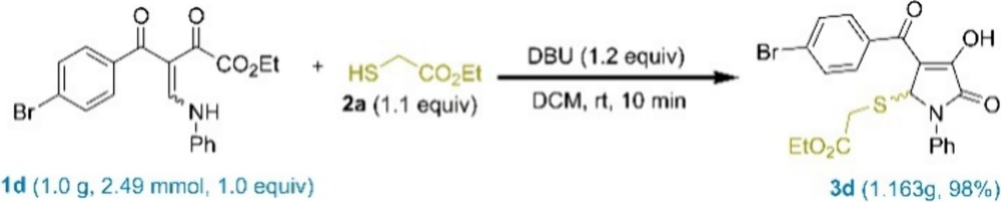
Gram-Scale Synthesis

To gain insight into the tandem reaction mechanism, a series of experiments were conducted, as outlined in Scheme 4. The addition of TEMPO, a well-known radical scavenger, did not inhibit this reaction, suggesting that the process likely follows a nonradical pathway. In contrast, no reaction occurred in the absence of DBU, highlighting its essential role in promoting the transformation. Further evidence for direct interaction between DBU and BED **1a** was obtained by NMR analysis. Upon addition of DBU to a solution of **1a** in DCM, the reaction mixture immediately changed color, consistent with the rapid formation of a new species. In the ^1^H NMR spectrum, DBU exhibited pronounced downfield shifts, especially those assigned to H-2, H-6, H-9, H-10, and H-11, indicating substantial decreased electron density at these sites (see for full details). Notably, the methylene protons at C-6 of DBU became diastereotopic, appearing as distinct signals with different chemical shifts and coupling constants, which is consistent with loss of symmetry upon adduct formation (Scheme 4c). HMBC correlation between H-6a/H-6b and C-5, together with the correlation between H-5 and C-6, supports the assignment of Int-**II**. When ethyl thioglycolate was added to a solution of Int-**II** in DCM, complete consumption of this intermediate was observed within 10 min, accompanied by the formation of a new species assigned as Int-**IV** based on NMR analysis of the crude reaction mixture. In this species, the C-6 protons of DBU became equivalent, and no log-range ^1^H–^13^C correlations were observed between DBU and the pyrrolone framework, consistent with the dissociation of the DBU fragment and the formation of Int-**IV**. Collectively, these observations support a nucleophile-initiated pathway for the cascade process.

**Scheme 4:**
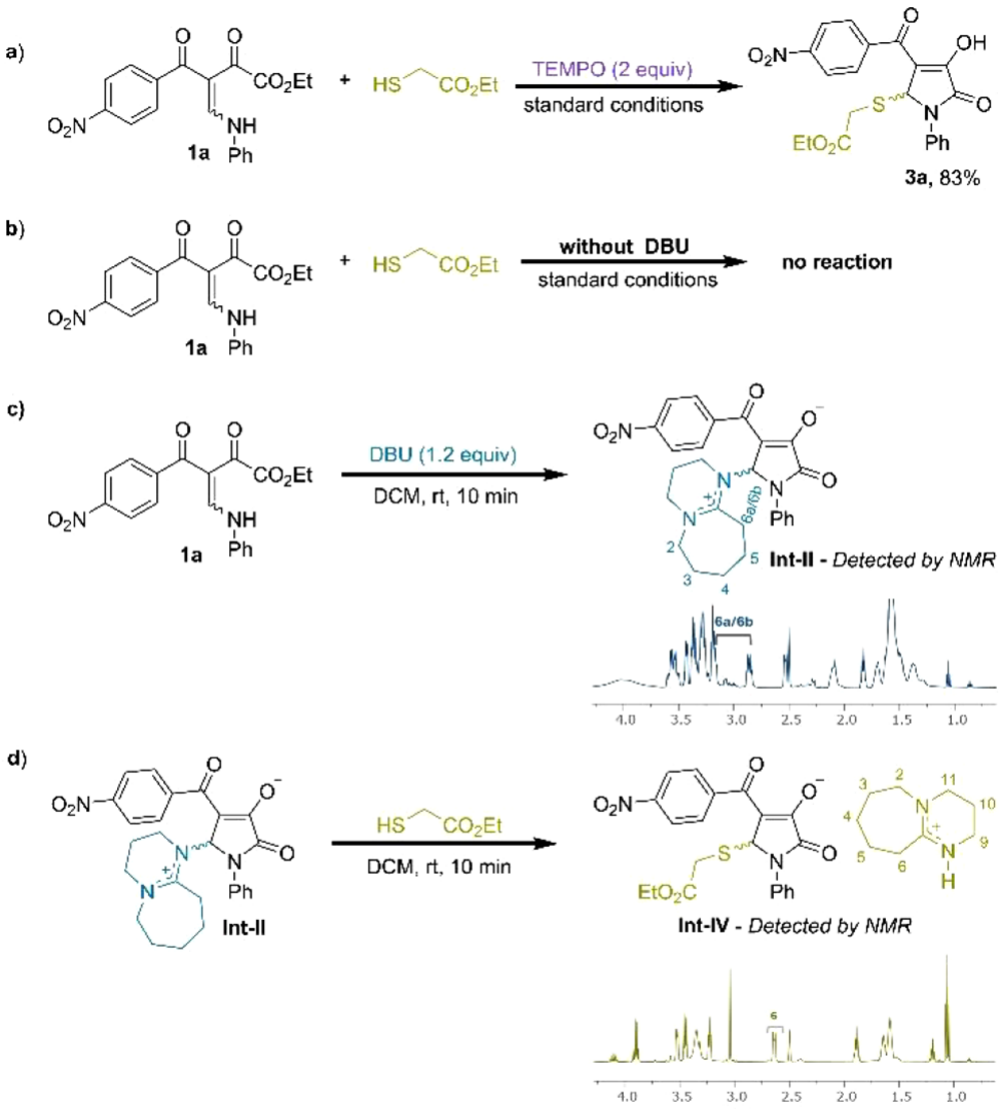
Control Experiments

Based on these experiments and literature precedents,^9^ a tentative mechanism for the DBU-promoted cyclization/thiolation of BEDs with thiols is proposed (Scheme 5). Initially, DBU promotes intramolecular lactamization of BED **1** via base catalysis, giving pyrrol-2,3-dione **I**. Conjugate addition of DBU to cyclic intermediate **I** then furnishes zwitterionic enolate **II**. This enolate acts as a Brønsted base, deprotonating the thiol to generate the corresponding thiolate, which reacts with activated intermediate **III** to form specie **IV**. Finally, proton transfer and neutralization of **IV** provided the desired thiolated product **3**, regenerating DBU. Notably, DBU appears to play a dual role, acting as both a nucleophilic catalyst and a base.

**Scheme 5:**
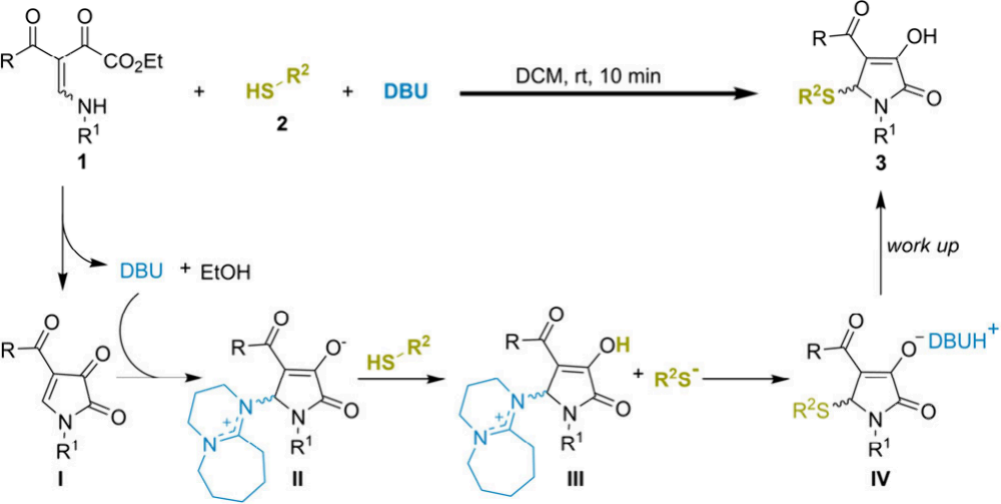
Plausible Reaction Mechanism

## Conclusion

In summary, we have developed a one-pot DBU-promoted cyclization/thiolation cascade that enables direct access to 5-sulfenylated 3-hydroxy-γ-lactams from β-enamino diketones and thiols under mild conditions. This method tolerates a wide range of *N*-aryl and *N*-alkyl substitution patterns and accommodates distinct sulfur nucleophiles, providing more than 20 γ-sulfenylated products in generally high to excellent yields. These results substantially expand the structural diversity of 3-hydroxy-γ-lactam scaffolds, addressing the long-standing challenge of γ-heterofunctionalization in this family. Mechanistic investigations indicated that DBU plays a dual role, promoting intramolecular lactamization and generating a nucleophilic zwitterionic enolate that activates the thiol and drives the tandem sequence. The combination of simplicity, efficiency, and broad applicability highlights this transformation as a useful platform for the rapid construction and further diversification of γ-lactam-based architectures that are of potential interest in medicinal chemistry.

## Supplementary Material





## Data Availability

The data underlying this study are available in the published article and its .

## Notes

Experimental Section with detailed experimental procedures, characterization data of all of the new compounds, and copies of NMR spectra of the products (PDF)
FAIR data, including the primary NMR FID files, for compounds **3a**–**3x** (ZIP)
