# Supplementary material for: DBU-Promoted Cascade Cyclization/Thiolation of β‑Enamino Diketones to 5‑Sulfenylated 3‑Hydroxy-γ-lactams
Source: J Org Chem. 2026 May 4;91(19):6610–4. doi: 10.1021/acs.joc.6c00140 (PMC13185102; doi:10.1021/acs.joc.6c00140)
Supplement: Supplementary file 2 [file jo6c00140_si_002.pdf]

DBU-promoted cascade cyclization/thiolation of β-enamino diketones to 5-sulfenylated 3-hydroxy-γ-lactams

Julia Poletto, Julia C. M. Willig, Jeniffer N. A. Camargo and Fernanda A. Rosa*

Laboratory for Synthesis of Heterocycles (SINTHET), Chemistry Department, State University of Maringá, Maringá, Paraná, 87020-900, Brazil.

*Email: farosa@uem.br

Table of contents

1. General Information……………………………………………………..………….……………...S1

2. Experimental Section.......................................……………..…………..………………….…..…...S1

3. ^{1}H and $^{13}C\{^{1}H\}$ NMR Spectra for the 5-Sulfenylated 3-hydroxy-γ-lactams S19

4. References………………………………………………………………………………..….....S65

1. General Information

Reagents and Solvents

All reactions were performed in conventional glassware. All reagents and solvents were purchased from commercial suppliers. Solvents were dried and purified according to recommended procedures.[ref.1]

Analysis and Characterization

Analytical TLC was performed on Merck aluminium-backed silica gel 60 F254 plates and visualized with UV light (254 nm). NMR spectra were recorded at 298 K on Bruker Avance III spectrometers, operating at nominal ^{1}H frequencies of 300 and 500 MHz, and internally referenced to residual solvent signals: DMSO-d_6 δ 2.50 (^{1}H). Chemical shifts (δ) are reported in ppm, coupling constants (J) are given in Hertz (Hz). Signals are reported as singlet (s), doublet (d), triplet (t), quartet (q), multiplet (m), broad (br). 13C NMR spectra were recorded at 298 K on Bruker Avance III spectrometers, operating at nominal ^{1}H frequencies of 75 and 125 MHz, and internally referenced to residual solvent signals: DMSO-d_6 δ 39.5 (^{13}C). Structural assignments were made with additional information from gHSQC and gHMBC experiments. HRMS spectra were acquired on a Bruker MicrOTOF Q-TOF instrument.

2. Experimental Section

2.1 General Procedure for synthesis of 3

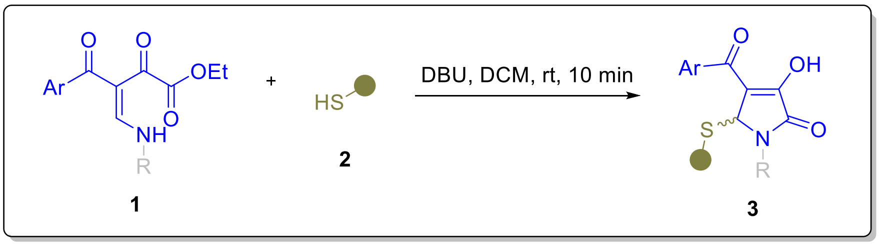

To a solution of β-enamino diketone **1** (0.2 mmol, 1.0 equiv) in DCM (3 mL), DBU (0.24 mmol, 1.2 equiv, 0.037 g) and the corresponding thiol **2** (0.22 mmol, 1.1 equiv) were added. The reaction mixture was stirred at room temperature for 10 min, with progress monitored via TLC. Subsequently, the solvent was removed under reduced pressure, and MeOH (3 mL) followed by 10% aqueous HCl (7 mL) were added to the residue. The resulting solid was collected by filtration, washed with cold distilled H_2O (10 mL), and dried in vacuo, affording the corresponding products **3** in good yields without further purification.

Note:
The preparation of β-enamino diketones **1**[ref.2] followed previously reported procedures, and all these compounds are known.

2.2 Scale-up experiment

To a solution of β-enamino diketone **1d** (2.49 mmol, 1.0 equiv, 1.0 g) in DCM (10 mL), DBU (2.99 mmol, 1.2 equiv, 0.464 g) and ethyl thioglycolate **2a** (2.74 mmol, 1.1 equiv, 0.339 g) were added. The reaction mixture was stirred at room temperature for 10 min, with progress monitored via TLC. Subsequently, the solvent was removed under reduced pressure, and MeOH (5 mL) followed by 10% aqueous HCl (13 mL) were added to the residue. The resulting solid was collected by filtration, washed with cold distilled H_2O (25 mL), and dried in vacuo to afford **3d** (1.163 g, 98% yield) as a white amorphous solid.

Ethyl 2-((4-hydroxy-3-(4-nitrobenzoyl)-5-oxo-1-phenyl-2,5-dihydro-1*H*-pyrrol-2-yl)thio)acetate (3a)

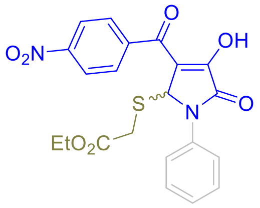

3a

The compound **3a** was synthesized from β-enamino diketone **1a** (Ar = 4-$NO_2C_6H_4$, R = Ph; 0.2 mmol, 1.0 equiv., 0.074 g) in DCM (3 mL), DBU (0.24 mmol, 1.2 equiv, 0.037 g) and ethyl thioglycolate **2a** (0.22 mmol, 1.1 equiv., 0.026 g) were added. The reaction mixture was stirred at room temperature for 10 min, with progress monitored via TLC. Subsequently, the solvent was removed under reduced pressure, and MeOH (3 mL) followed by 10% aqueous HCl (7 mL) were added to the residue. The resulting solid was collected by filtration, washed with cold distilled H_2O (10 mL), and dried in vacuo to give **3a** (0.080 g, 90% yield) as white amorphous solid.

^{1}H NMR (300.06 MHz, DMSO-*d$_6$*): δ 8.35 (d, *J* = 8.9 Hz, 2H), 8.02 (d, *J* = 8.9 Hz, 2H), 7.69-7.66 (m, 2H), 7.50-7.45 (m, 2H), 7.33-7.27 (m, 1H), 6.44 (s, 1H), 3.92 (q, *J* = 7.1 Hz, 2H), 3.09 (d, *J* = 1.5 Hz, 2H), 1.06 (t, *J* = 7.1 Hz, 3H).
^{13}C{^{1}H} NMR (75.46 MHz, DMSO-*d$_6$*): δ 186.7, 169.5, 163.2, 153.4, 149.5, 143.3, 135.2, 130.1, 128.8, 126.2, 123.4, 114.9, 61.2, 61.1, 28.8, 13.8.
HRMS (ESI-) *m/z*: $[M-H]^-$ Calcd for $C_{21}H_{17}N_2O_7S^-$ 441.0762; Found 441.0780.

Ethyl 2-((3-(4-fluorobenzoyl)-4-hydroxy-5-oxo-1-phenyl-2,5-dihydro-1*H*-pyrrol-2-yl)thio)acetate (3b)

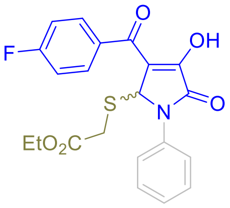

3b

The compound **3b** was prepared from β-enamino diketone **1b** (Ar = 4-FC_6H_4, R = Ph; 0.2 mmol, 1.0 equiv., 0.068 g) in DCM (3 mL), DBU (0.24 mmol, 1.2 equiv, 0.037 g) and ethyl thioglycolate **2a** (0.22 mmol, 1.1 equiv., 0.026 g) were added. The reaction mixture was stirred at room temperature for 10 min, with progress monitored via TLC. Subsequently, the solvent was removed under reduced pressure, and MeOH (3 mL) followed by 10% aqueous HCl (7 mL) were added to the residue. The resulting solid was collected by filtration, washed with cold distilled H_2O (10 mL), and dried in vacuo to give **3b** (0.079 g, 96% yield) as white amorphous solid.

^{1}H NMR (300.06 MHz, DMSO-*d$_6$*): δ 7.97-7.92 (m, 2H), 7.69-7.65 (m, 2H, Ph), 7.49-7.44 (m, 2H, Ph), 7.40-7.34 (m, 2H), 7.31-7.26 (m, 1H), 6.42 (s, 1H), 3.89 (q, *J* = 7.1 Hz, 2H), 3.06 (s, 2H), 1.04 (t, *J* = 7.1 Hz, 3H).
^{13}C{^{1}H} NMR (75.46 MHz, DMSO-*d$_6$*): δ 186.7, 169.4, 164.9 (d, *J* = 251.3 Hz), 163.3, 151.2, 135.3, 134.3 (d, *J* = 2.8 Hz), 132.1 (d, *J* = 9.4 Hz), 128.7, 126.0, 123.4, 115.7, 115.3 (d, *J* = 21.9 Hz), 61.4, 61.0, 28.8. 13.7.
HRMS (ESI-) *m/z*: $[M-H]^-$ Calcd for $C_{21}H_{17}FNO_5S^-$ 414.0817; Found 414.0822.

Ethyl 2-((3-(4-chlorobenzoyl)-4-hydroxy-5-oxo-1-phenyl-2,5-dihydro-1*H*-pyrrol-2-yl)thio)acetate (3c)

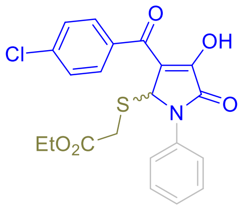

3c

The compound **3c** was prepared from β-enamino diketone **1c** (Ar = 4-ClC_6H_4, R = Ph; 0.2 mmol, 1.0 equiv., 0.071 g) in DCM (3 mL), DBU (0.24 mmol, 1.2 equiv, 0.037 g) and ethyl thioglycolate **2a** (0.22 mmol, 1.1 equiv., 0.026 g) were added. The reaction mixture was stirred at room temperature for 10 min, with progress monitored via TLC. Subsequently, the solvent was removed under reduced pressure, and MeOH (3 mL) followed by 10% aqueous HCl (7 mL) were added to the residue. The resulting solid was collected by filtration, washed with cold distilled H_2O (10 mL), and dried in vacuo to give **3c** (0.080 g, 93% yield) as white amorphous solid.

^{1}H NMR (300.06 MHz, DMSO-*d₆*): δ 7.86 (d, J = 8.6 Hz, 2H), 7.68-7.65 (m, 2H), 7.61 (d, J = 8.6 Hz, 2H), 7.49-7.44 (m, 2H), 7.31-7.26 (m, 1H), 6.42 (s, 1H), 3.90 (q, J = 7.1 Hz, 2H), 3.06 (s, 2H), 1.04 (t, J = 7.1 Hz, 3H).
^{13}C{^{1}H} NMR (75.46 MHz, DMSO-d_6): δ 187.0, 169.4, 163.3, 151.7, 137.7, 136.5, 135.3, 131.0, 128.7, 128.4, 126.0, 123.4, 115.5, 61.4, 61.1, 28.8, 13.8.
HRMS (ESI-) *m/z*: $[M-H]^-$ Calcd for $C_{21}H_{17}ClNO_5S^-$ 430.0521; Found, 430.0537.

Ethyl 2-((3-(4-bromobenzoyl)-4-hydroxy-5-oxo-1-phenyl-2,5-dihydro-1*H*-pyrrol-2-yl)thio)acetate (3d)

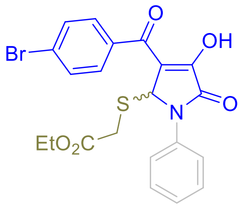

3d

The compound **3d** was prepared from β-enamino diketone **1d** (Ar = 4-BrC_6H_4, R = Ph; 0.2 mmol, 1.0 equiv., 0.080 g) in DCM (3 mL), DBU (0.24 mmol, 1.2 equiv, 0.037 g) and ethyl thioglycolate **2a** (0.22 mmol, 1.1 equiv., 0.026 g) were added. The reaction mixture was stirred at room temperature for 10 min, with progress monitored via TLC. Subsequently, the solvent was removed under reduced pressure, and MeOH (3 mL) followed by 10% aqueous HCl (7 mL) were added to the residue. The resulting solid was collected by filtration, washed with cold distilled H_2O (10 mL), and dried in vacuo to give **3d** as white amorphous solid. Yield: 0.089 g, 93%, from 0.080 g of **1d**, and 1.163 g, 98%, from 1.0 g of **1d**).

^{1}H NMR (300.06 MHz, DMSO-d_6): δ 7.77-7.76 (m, 4H), 7.68-7.64 (m, 2H), 7.49-7.44 (m, 2H), 7.31-7.26 (m, 1H), 6.42 (s, 1H), 3.90 (q, J = 7.1 Hz, 2H), 3.06 (s, 2H), 1.04 (t, J = 7.1 Hz, 3H).
^{13}C{^{1}H} NMR (75.46 MHz, DMSO-d_6): δ 187.2, 169.4, 163.3, 151.7, 136.8, 135.3, 131.3, 131.0, 128.7, 126.8, 126.0, 123.4, 115.5, 61.3, 61.0, 28.8, 13.7.
HRMS (ESI-) *m/z*: $[M-H]^-$ Calcd for $C_{21}H_{17}BrNO_5S^-$ 474.0016; Found, 474.0036.

Ethyl 2-((3-benzoyl-4-hydroxy-5-oxo-1-phenyl-2,5-dihydro-1*H*-pyrrol-2-yl)thio)acetate (3e)

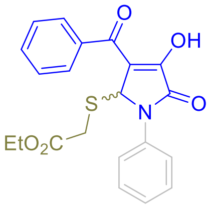

3e

The compound **3e** was prepared from β-enamino diketone **1e** (Ar = Ph, R = Ph; 0.2 mmol, 1.0 equiv., 0.065 g) in DCM (3 mL), DBU (0.24 mmol, 1.2 equiv, 0.037 g) and ethyl thioglycolate **2a** (0.22 mmol, 1.1 equiv., 0.026 g) were added. The reaction mixture was stirred at room temperature for 10 min, with progress monitored via TLC. Subsequently, the solvent was removed under reduced pressure, and MeOH (3 mL) followed by 10% aqueous HCl (7 mL) were added to the residue. The resulting solid was collected by filtration, washed with cold distilled H_2O (10 mL), and dried in vacuo to give **3e** (0.071 g, 89% yield) as white amorphous solid.

^{1}H NMR (300.06 MHz, DMSO-*d$_6$*): δ 7.88-7.85 (m, 2H), 7.69-7.62 (m, 3H), 7.56-7.53 (m, 2H), 7.51-7.49 (m, 1H), 7.47-7.44 (m, 2H), 7.31-7.26 (m, 1H), 6.43 (s, 1H), 3.90 (q, *J* = 7.1 Hz, 2H), 3.07 (s, 2H), 1.04 (t, *J* = 7.1 Hz, 3H).
^{13}C{^{1}H} NMR (75.46 MHz, DMSO-*d$_6$*): δ 188.2, 169.3, 163.4, 151.1, 137.6, 135.3, 132.9, 129.1, 128.7, 128.3, 126.0, 123.3, 115,8, 61.5, 61.0, 28.8, 13.7.
HRMS (ESI-) *m/z*: [M-H]$^-$ Calcd for $C_{21}H_{18}NO_5S^-$ 396.0911; Found, 396.0927.

Ethyl 2-((4-hydroxy-3-(4-methoxybenzoyl)-5-oxo-1-phenyl-2,5-dihydro-1*H*-pyrrol-2-yl)thio)acetate (3f)

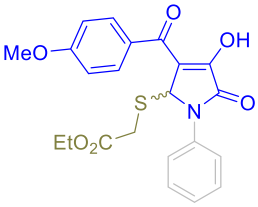

3f

The compound **3f** was prepared from β-enamino diketone **1f** (Ar = 4-OMeC_6H_4, R = Ph; 0.2 mmol, 1.0 equiv., 0.071 g) in DCM (3 mL), DBU (0.24 mmol, 1.2 equiv, 0.037 g) and ethyl thioglycolate **2a** (0.22 mmol, 1.1 equiv., 0.026 g) were added. The reaction mixture was stirred at room temperature for 10 min, with progress monitored via TLC. Subsequently, the solvent was removed under reduced pressure, and MeOH (3 mL) followed by 10% aqueous HCl (7 mL) were added to the residue. The resulting solid was collected by filtration, washed with cold distilled H_2O (10 mL), and dried in vacuo to give **3f** (0.071 g, 83% yield) as white amorphous solid.

^{1}H NMR (300.06 MHz, DMSO-*d$_6$*): δ 7.89 (d, *J* = 8.9 Hz, 2H), 7.68-7.65 (m, 2H), 7.49-7.43 (m, 2H), 7.31-7.25 (m, 1H), 7.07 (d, *J* = 8.9 Hz, 2H), 6.42 (s, 1H), 3.88 (q, *J* = 7.1 Hz, 2H), 3.87 (s, 3H), 3.05 (s, 2H), 1.04 (t, *J* = 7.1 Hz, 3H).
^{13}C{^{1}H} NMR (75.46 MHz, DMSO-*d$_6$*): δ 186.7, 169.2, 163.5, 163.3, 149.7, 135.4, 131.7, 130.1, 128.7, 125.9, 123.3, 116.5, 113.6, 61.6, 61.0, 55.6, 28.8, 13.7.
HRMS (ESI-) *m/z*: [M-H]$^-$ Calcd for $C_{22}H_{20}NO_6S^-$ 426.1017; Found, 426.1031.

Ethyl 2-((4-hydroxy-1-(4-methoxyphenyl)-3-(4-nitrobenzoyl)-5-oxo-2,5-dihydro-1*H*-pyrrol-2-yl)thio)acetate (3g)

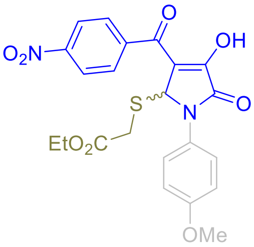

3g

The compound **3g** was prepared from β-enamino diketone **1g** (Ar = 4-NO2C$_6$H$_4$, R = 4-OMeC$_6$H$_4$; 0.2 mmol, 1.0 equiv., 0.080 g) in DCM (3 mL), DBU (0.24 mmol, 1.2 equiv, 0.037 g) and ethyl thioglycolate **2a** (0.22 mmol, 1.1 equiv., 0.026 g) were added. The reaction mixture was stirred at room temperature for 10 min, with progress monitored via TLC. Subsequently, the solvent was removed under reduced pressure, and MeOH (3 mL) followed by 10% aqueous HCl (7 mL) were added to the residue. The resulting solid was collected by filtration, washed with cold distilled H_2O (10 mL), and dried in vacuo to give **3g** (0.090 g, 95% yield) as white amorphous solid.

^{1}H NMR (300.06 MHz, DMSO-*d$_6$*): δ 8.35 (d, *J* = 8.9 Hz, 2H), 8.01 (d, *J* = 8.9 Hz, 2H), 7.56 (d, *J* = 9.0 Hz, 2H), 7.03 (d, *J* = 9.0 Hz, 2H), 6.35 (s, 1H), 3.94 (q, *J* = 7.1 Hz, 2H), 3.79 (s, 3H), 3.07 (d, *J* = 3.3 Hz, 2H), 1.07 (t, *J* = 7.1 Hz, 3H).
^{13}C{^{1}H} NMR (75.46 MHz, DMSO-*d$_6$*): δ 186.7, 169.5, 163.0, 157.5, 153.6, 149.4, 143.4, 130.1, 128.1, 125.3, 123.4, 114.8, 114.0, 61.7, 61.1, 55.4, 28.8, 13.8.
HRMS (ESI-) *m/z*: [M-H]$^-$ Calcd for $C_{22}H_{19}N_2O_8S^-$ 471.0868; Found, 471.0883.

Ethyl 2-((3-(4-fluorobenzoyl)-4-hydroxy-1-(4-methoxyphenyl)-5-oxo-2,5-dihydro-1*H*-pyrrol-2-yl)thio)acetate (3h)

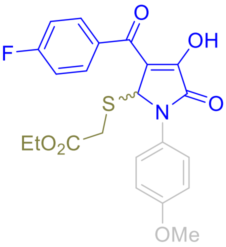

3h

The compound **3h** was prepared from β-enamino diketone **1h** (Ar = 4-FC$_6$H$_4$, R = 4-OMeC$_6$H$_4$; 0.2 mmol, 1.0 equiv., 0.074 g) in DCM (3 mL), DBU (0.24 mmol, 1.2 equiv, 0.037 g) and ethyl thioglycolate **2a** (0.22 mmol, 1.1 equiv., 0.026 g) were added. The reaction mixture was stirred at room temperature for 10 min, with progress monitored via TLC. Subsequently, the solvent was removed under reduced pressure, and MeOH (3 mL) followed by 10% aqueous HCl (7 mL) were added to the residue. The resulting solid was collected by filtration, washed with cold distilled H_2O (10 mL), and dried in vacuo to give **3h** (0.087 g, 98% yield) as white amorphous solid.

^{1}H NMR (300.06 MHz, DMSO-*d$_6$*): δ 7.95-7.90 (m, 2H), 7.55 (d, *J* = 9.0 Hz, 2H), 7.40-7.34 (m, 2H), 7.02 (d, *J* = 9.0 Hz, 2H), 6.34 (s, 1H), 3.91 (q, *J* = 7.1 Hz, 2H), 3.79 (s, 3H), 3.05 (d, *J* = 1.8 Hz, 2H), 1.06 (t, *J* = 7.1 Hz, 3H).
^{13}C{^{1}H} NMR (75.46, MHz DMSO-*d$_6$*): δ 186.6, 169.4, 164.9 (d, *J* = 251.2 Hz), 163.1, 157.4, 151.4, 134.3 (d, *J* = 2.8 Hz), 132.0 (d, *J* = 9.4 Hz), 128.2, 125.2, 115.6, 115.3 (d, *J* = 22.0 Hz), 114.0, 61.9, 61.0, 55.3, 28.8, 13.7. **HRMS (ESI-)** *m/z*: [M-H]$^-$ Calcd for $C_{22}H_{19}FNO_6S^-$ 444.0923; Found, 444.0938.

Ethyl 2-((3-(4-chlorobenzoyl)-4-hydroxy-1-(4-methoxyphenyl)-5-oxo-2,5-dihydro-1*H*-pyrrol-2-yl)thio)acetate (3i)

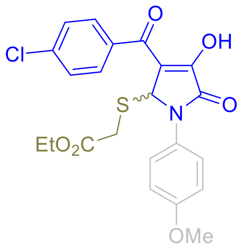

3i

The compound **3i** was prepared from β-enamino diketone **1i** (Ar = 4-ClC_6H_4, R = 4-OMeC_6H_4; 0.2 mmol, 1.0 equiv., 0.077 g) in DCM (3 mL), DBU (0.24 mmol, 1.2 equiv, 0.037 g) and ethyl thioglycolate **2a** (0.22 mmol, 1.1 equiv., 0.026 g) were added. The reaction mixture was stirred at room temperature for 10 min, with progress monitored via TLC. Subsequently, the solvent was removed under reduced pressure, and MeOH (3 mL) followed by 10% aqueous HCl (7 mL) were added to the residue. The resulting solid was collected by filtration, washed with cold distilled H_2O (10 mL), and dried in vacuo to give **3i** (0.084 g, 92% yield) as white amorphous solid.

^{1}H NMR (300.06 MHz, DMSO-*d$_6$*): δ 7.84 (d, *J* = 8.6 Hz, 2H), 7.60 (d, *J* = 8.6 Hz, 2H), 7.55 (d, *J* = 9.1 Hz, 2H), 7.02 (d, *J* = 9.1 Hz, 2H), 6.34 (s, 1H), 3.92 (q, *J* = 7.1 Hz, 2H), 3.79 (s, 3H), 3.04 (d, *J* = 1.9 Hz, 2H), 1.06 (t, *J* = 7.1 Hz, 3H).
^{13}C{^{1}H} NMR (75.46 MHz, DMSO-*d$_6$*): δ 186.9, 169.4, 163.1, 157.5, 152.0, 137.6, 136.5, 130.9, 128.4, 128.2, 125.2, 115.3, 114.0, 61.9, 61.0, 55.3, 28.8, 13.8.
HRMS (ESI-) *m/z*: [M-H]$^-$ Calcd for $C_{22}H_{19}ClNO_6S^-$ 460.0627; Found, 460.0646.

Ethyl 2-((3-(4-bromobenzoyl)-4-hydroxy-1-(4-methoxyphenyl)-5-oxo-2,5-dihydro-1*H*-pyrrol-2-yl)thio)acetate (3j)

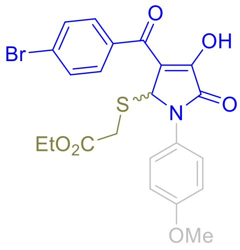

3j

The compound **3j** was prepared from β-enamino diketone **1j** (Ar = 4-BrC_6H_4, R = 4-OMeC_6H_4; 0.2 mmol, 1.0 equiv., 0.086 g) in DCM (3 mL), DBU (0.24 mmol, 1.2 equiv, 0.037 g) and ethyl thioglycolate **2a** (0.22 mmol, 1.1 equiv., 0.026 g) were added. The reaction mixture was stirred at room temperature for 10 min, with progress monitored via TLC. Subsequently, the solvent was removed under reduced pressure, and MeOH (3 mL) followed by 10% aqueous HCl (7 mL) were added to the residue. The resulting solid was collected by filtration, washed with cold distilled H_2O (10 mL), and dried in vacuo to give **3j** (0.095 g, 94% yield) as white amorphous solid.

^{1}H NMR (300.06 MHz, DMSO-*d$_6$*): δ 7.75 (s, 4H), 7.55 (d, *J* = 9.0 Hz, 2H), 7.02 (d, *J* = 9.0 Hz, 2H), 6.33 (s, 1H), 3.92 (q, *J* = 7.1 Hz, 2H), 3.79 (s, 3H), 3.04 (d, *J* = 1.9 Hz, 2H), 1.06 (t, *J* = 7.1 Hz, 3H).
^{13}C{^{1}H} NMR (75.46 MHz, DMSO-*d$_6$*): δ 187.1, 169.4, 163.1, 157.5, 152.0, 136.9, 131.3, 131.0, 128.1, 126.7, 125.2, 115.3, 114.0, 61.9, 61.0, 55.3, 28.8, 13.8.
HRMS (ESI-) *m/z*: [M-H]$^-$ Calcd for $C_{22}H_{19}BrNO_6S^-$ 504.0122; Found, 504.0149.

Ethyl 2-((3-benzoyl-4-hydroxy-1-(4-methoxyphenyl)-5-oxo-2,5-dihydro-1*H*-pyrrol-2-yl)thio)acetate (3k)

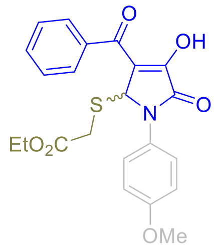

3k

The compound **3k** was prepared from β-enamino diketone **1k** (Ar = Ph, R = 4-OMeC_6H_4; 0.2 mmol, 1.0 equiv., 0.077 g) in DCM (3 mL), DBU (0.24 mmol, 1.2 equiv, 0.037 g) and ethyl thioglycolate **2a** (0.22 mmol, 1.1 equiv., 0.026 g) were added. The reaction mixture was stirred at room temperature for 10 min, with progress monitored via TLC. Subsequently, the solvent was removed under reduced pressure, and MeOH (3 mL) followed by 10% aqueous HCl (7 mL) were added to the residue. The resulting solid was collected by filtration, washed with cold distilled H_2O (10 mL), and dried in vacuo to give **3k** (0.078 g, 91% yield) as white amorphous solid.

^{1}H NMR (300.06 MHz, DMSO-*d$_6$*): δ 7.86-7.83 (m, 2H), 7.67-.7.61 (m, 1H), 7.50-7.55 (m, 2H), 7.56 (d, *J* = 9.1 Hz, 2H), 7.02 (d, *J* = 9.0 Hz, 2H), 6.35 (s, 1H), 3.91 (q, *J* = 7.1 Hz, 2H), 3.79 (s, 3H), 3.05 (d, *J* = 2.4 Hz, 2H), 1.06 (t, *J* = 7.1 Hz, 3H).
^{13}C{^{1}H} NMR (75.46 MHz, DMSO-*d$_6$*): δ 188.2, 169.3, 163.2, 157.4, 151.3, 137.7, 132.9, 129.1, 128.2, 125.2, 115.7, 114.0, 62.0, 61.0, 55.3, 28.9, 13.7.
HRMS (ESI-) *m/z*: [M-H]$^-$ Calcd for $C_{22}H_{20}NO_6S^-$ 426.1017; Found, 426.1033.

Ethyl 2-((4-hydroxy-3-(4-methoxybenzoyl)-1-(4-methoxyphenyl)-5-oxo-2,5-dihydro-1*H*-pyrrol-2-yl)thio)acetate (3l)

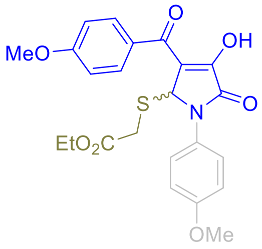

3l

The compound **3l** was prepared from β-enamino diketone **1l** (Ar = 4-OMeC_6H_4, R = 4-OMeC_6H_4; 0.2 mmol, 1.0 equiv., 0.077 g) in DCM (3 mL), DBU (0.24 mmol, 1.2 equiv, 0.037 g) and ethyl thioglycolate **2a** (0.22 mmol, 1.1 equiv., 0.026 g) were added. The reaction mixture was stirred at room temperature for 10 min, with progress monitored via TLC. Subsequently, the solvent was removed under reduced pressure, and MeOH (3 mL) followed by 10% aqueous HCl (7 mL) were added to the residue. The resulting solid was collected by filtration, washed with cold distilled H_2O (10 mL), and dried in vacuo to give **3l** (0.074 g, 80% yield) as white amorphous solid.

^{1}H NMR (300.06 MHz, DMSO-*d$_6$*): δ 7.88 (d, *J* = 8.9 Hz, 2H), 7.55 (d, *J* = 9.0 Hz, 2H), 7.06 (d, *J* = 8.9 Hz, 2H), 7.02 (d, *J* = 9.0 Hz, 2H), 6.34 (s, 1H), 3.90 (q, *J* = 7.1 Hz, 2H), 3.87 (s, 3H), 3.79 (s, 3H), 3.04 (d, *J* = 1.7 Hz, 2H), 1.06 (t, *J* = 7.1 Hz, 3H).
^{13}C{^{1}H} NMR (75.46 MHz, DMSO-*d$_6$*): δ 186.7, 169.3, 163.3, 157.4, 150.0, 131.7, 130.2, 128.3, 125.2, 116.3, 114.0, 113.6, 62.1, 61.0, 55.6, 55.3, 28.9, 13.7.
HRMS (ESI-) *m/z*: [M-H]$^-$ Calcd for $C_{23}H_{22}NO_7S^-$ 456.1122; Found, 456.1134.

Ethyl 2-((4-hydroxy-3-(4-nitrobenzoyl)-1-(4-nitrophenyl)-5-oxo-2,5-dihydro-1*H*-pyrrol-2-yl)thio)acetate (3m)

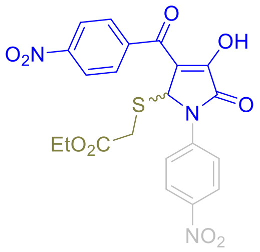

3m

The compound **3m** was prepared from β-enamino diketone **1m** (Ar = 4-$NO_2C_6H_4$, R = 4-$NO_2C_6H_4$; 0.2 mmol, 1.0 equiv., 0.083 g) in DCM (3 mL), DBU (0.24 mmol, 1.2 equiv, 0.037 g) and ethyl thioglycolate **2a** (0.22 mmol, 1.1 equiv., 0.026 g) were added. The reaction mixture was stirred at room temperature for 10 min, with progress monitored via TLC. Subsequently, the solvent was removed under reduced pressure, and MeOH (3 mL) followed by 10% aqueous HCl (7 mL) were added to the residue. The resulting solid was collected by filtration, washed with cold distilled H_2O (10 mL), and dried in vacuo to give **3m** (0.091 g, 93% yield) as light yellow amorphous solid.

^{1}H NMR (500.13 MHz, DMSO-*d₆*): δ 8.36 (d, *J* = 8.7 Hz, 2H), 8.35 (d, *J* = 9.3 Hz, 2H), 8.06-8.04 (m, 4H), 6.57 (s, 1H), 3.90-3.87 (m, 2H), 3.14 (d, *J* = 3.6 Hz, 2H), 1.03 (t, *J* = 7.1 Hz, 3H).
^{13}C{^{1}H} NMR (125.77 MHz, DMSO-*d₆*): δ 186.8, 169.5, 164.0, 153.3, 149.6, 144.2, 143.3, 141.2, 130.3, 124.4, 123.5, 122.7, 114.9, 61.3, 28.8, 13.8.
HRMS (ESI+) *m/z*: **[M + H]$^+$** Calcd for $C_{21}H_{18}N_3O_9S^+$ 488.0758; Found, 488.0750.

Ethyl 2-((4-hydroxy-1-methyl-3-(4-nitrobenzoyl)-5-oxo-2,5-dihydro-1*H*-pyrrol-2-yl)thio)acetate (3n)

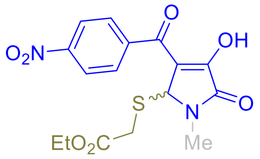

3n

The compound **3n** was prepared from β-enamino diketone **1n** (Ar = 4-$NO_2C_6H_4$, R = Me; 0.2 mmol, 1.0 equiv., 0.061 g) in DCM (3 mL), DBU (0.24 mmol, 1.2 equiv, 0.037 g) and ethyl thioglycolate **2a** (0.22 mmol, 1.1 equiv., 0.026 g) were added. The reaction mixture was stirred at room temperature for 10 min, with progress monitored via TLC. Subsequently, the solvent was removed under reduced pressure, and MeOH (3 mL) followed by 10% aqueous HCl (7 mL) were added to the residue. The resulting solid was collected by filtration, washed with cold distilled H_2O (10 mL), and dried in vacuo to give **3n** (0.070 g, 92% yield) as white amorphous solid.

^{1}H NMR (500.13 MHz, DMSO-*d₆*): δ 8.32 (d, *J* = 8.9 Hz, 2H), 7.95 (d, *J* = 8.9 Hz, 2H), 5.61 (d, *J* = 0.7 Hz, 1H), 4.02-3.99 (m, 2H), 3.14 (s, 2H), 2.95 (d, *J* = 0.7 Hz, 3H), 1.15 (t, *J* = 7.1 Hz, 3H).
^{13}C{^{1}H} NMR (75.46 MHz, DMSO-*d₆*): δ 186.4, 169.8, 163.8, 154.6, 149.4, 143.4, 130.0, 123.4, 114.5, 62.8, 61.2, 28.8, 27.1, 13.9.
HRMS (ESI-) *m/z*: [M-H]$^-$ Calcd for $C_{16}H_{15}N_2O_7S^-$ 379.0605; Found, 379.0593.

Ethyl 2-((3-(4-fluorobenzoyl)-4-hydroxy-1-methyl-5-oxo-2,5-dihydro-1*H*-pyrrol-2-yl)thio)acetate (3o)

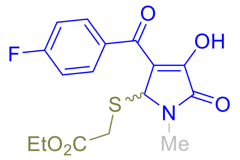

3o

The compound **3o** was prepared from β-enamino diketone **1o** (Ar = 4-FC_6H_4, R = Me; 0.2 mmol, 1.0 equiv., 0.056 g) in DCM (3 mL), DBU (0.24 mmol, 1.2 equiv, 0.037 g) and ethyl thioglycolate **2a** (0.22 mmol, 1.1 equiv., 0.026 g) were added. The reaction mixture was stirred at room temperature for 10 min, with progress monitored via TLC. Subsequently, the solvent was removed under reduced pressure, and MeOH (3 mL) followed by 10% aqueous HCl (7 mL) were added to the residue. The resulting solid was collected by filtration, washed with cold distilled H_2O (10 mL), and dried in vacuo to give **3o** (0.062 g, 88% yield) as white amorphous solid.

^{1}H NMR (300.06 MHz, DMSO-*d*$_6$): δ 7.89-1.84 (m, 2H), 7.37-7.31 (m, 2H), 5.60 (d, *J* = 0.7 Hz, 1H), 4.01-3.96 (m, 2H), 3.12 (d, *J* = 2.3 Hz, 2H), 2.94 (d, *J* = 0.7 Hz, 3H), 1.14 (t, *J* = 7.1 Hz, 3H).
^{13}C{^{1}H} NMR (75.46 MHz, DMSO-*d*$_6$): δ 186.4, 169.7, 164.8 (d, *J* = 251.1 Hz), 164.0, 152.4, 134.3 (d, *J* = 2.8 Hz), 131.9 (d, *J* = 9.4 Hz), 115.3 (d, *J* = 21.9 Hz), 115.2, 63.0, 61.1, 28.8, 27.1, 13.8,
HRMS (ESI-) *m/z*: $[M-H]^-$ Calcd for $C_{16}H_{15}FNO_5S^-$ 352.0660; Found, 352.0667.

Ethyl 2-((3-(4-chlorobenzoyl)-4-hydroxy-1-methyl-5-oxo-2,5-dihydro-1*H*-pyrrol-2-yl)thio)acetate (3p)

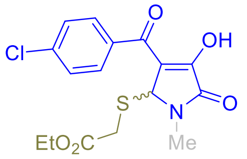

3p

The compound **3p** was prepared from β-enamino diketone **1p** (Ar = 4-ClC_6H_4, R = Me; 0.2 mmol, 1.0 equiv., 0.059 g) in DCM (3 mL), DBU (0.24 mmol, 1.2 equiv, 0.037 g) and ethyl thioglycolate **2a** (0.22 mmol, 1.1 equiv., 0.026 g) were added. The reaction mixture was stirred at room temperature for 10 min, with progress monitored via TLC. Subsequently, the solvent was removed under reduced pressure, and MeOH (3 mL) followed by 10% aqueous HCl (7 mL) were added to the residue. The resulting solid was collected by filtration, washed with cold distilled H_2O (10 mL), and dried in vacuo to give **3p** (0.066 g, 90% yield) as white amorphous solid.

^{1}H NMR (300.06 MHz, DMSO-*d*$_6$): δ 7.78 (d, *J* = 8.6 Hz, 2H), 7.58 (d, *J* = 8.6 Hz, 2H), 5.60 (d, *J* = 0.7 Hz, 1H), 4.01-3.96 (m, 2H), 3.11 (d, *J* = 2.0 Hz, 2H), 2.94 (d, *J* = 0.6 Hz, 3H), 1.14 (t, *J* = 7.1 Hz, 3H).
^{13}C{^{1}H} NMR (75.46 MHz, DMSO-*d*$_6$): δ 186.7, 169.7, 163.9, 152.9, 137.5, 136.5, 130.8, 128.4, 115.0, 63.0, 61.2, 28.8, 27.1, 13.9.
HRMS (ESI-) *m/z*: $[M-H]^-$ Calcd for $C_{16}H_{15}ClNO_5S^-$ 368.0365; Found, 368.0390.

Ethyl 2-((3-(4-bromobenzoyl)-4-hydroxy-1-methyl-5-oxo-2,5-dihydro-1*H*-pyrrol-2-yl)thio)acetate (3q)

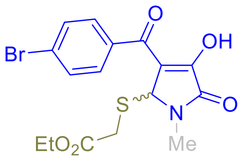

3q

The compound **3q** was prepared from β-enamino diketone **1q** (Ar = 4-BrC$_6$H$_4$, R = Me; 0.2 mmol, 1.0 equiv., 0.068 g) in DCM (3 mL), DBU (0.24 mmol, 1.2 equiv, 0.037 g) and ethyl thioglycolate **2a** (0.22 mmol, 1.1 equiv., 0.026 g) were added. The reaction mixture was stirred at room temperature for 10 min, with progress monitored via TLC. Subsequently, the solvent was removed under reduced pressure, and MeOH (3 mL) followed by 10% aqueous HCl (7 mL) were added to the residue. The resulting solid was collected by filtration, washed with cold distilled H_2O (10 mL), and dried in vacuo to give **3q** (0.067 g, 81% yield) as white amorphous solid.

^{1}H NMR (300.06 MHz, DMSO-*d$_6$*): δ 7.71 (m, 4H), 5.60 (d, *J* = 0.6 Hz, 1H), 4.02-3.96 (m, 2H), 3.11 (d, *J* = 2.1 Hz, 2H), 2.94 (d, *J* = 0.7 Hz, 3H), 1.14 (t, *J* = 7.1 Hz, 3H).
^{13}C{^{1}H} NMR (75.46 MHz, DMSO-*d$_6$*): δ 186.9, 169.7, 163.9, 152.9, 136.8, 131.3 130.9, 126.6, 115.0, 62.9, 61.1, 28.8, 27.1, 13.9.
HRMS (ESI-) *m/z*: [M-H]$^-$ Calcd for $C_{16}H_{15}BrNO_5S^-$ 411.9860; Found, 411.9880.

Ethyl 2-((3-benzoyl-4-hydroxy-1-methyl-5-oxo-2,5-dihydro-1*H*-pyrrol-2-yl)thio)acetate (3r)

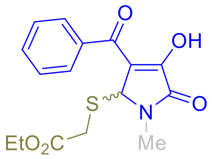

3r

The compound **3r** was prepared from β-enamino diketone **1r** (Ar = Ph, R = Me; 0.2 mmol, 1.0 equiv., 0.052 g) in DCM (3 mL), DBU (0.24 mmol, 1.2 equiv, 0.037 g) and ethyl thioglycolate **2a** (0.22 mmol, 1.1 equiv., 0.026 g) were added. The reaction mixture was stirred at room temperature for 10 min, with progress monitored via TLC. Subsequently, the solvent was removed under reduced pressure, and MeOH (3 mL) followed by 10% aqueous HCl (7 mL) were added to the residue. The resulting solid was collected by filtration, washed with cold distilled H_2O (10 mL), and dried in vacuo to give **3r** (0.053 g, 80% yield) as white amorphous solid.

^{1}H NMR (300.06 MHz, DMSO-*d$_6$*): δ 7.81-7.77 (m, 2H), 7.62-7.59 (m, 1H), 7.53-7.51 (m, 2H), 5.61 (d, *J* = 0.6 Hz, 1H), 4.03-3.97 (m, 2H), 3.13 (d, *J* = 3.0 Hz, 2H), 2.94 (d, *J* = 0.6 Hz, 3H), 1.15 (t, *J* = 7.1 Hz, 3H).
^{13}C{^{1}H} NMR (75.46 MHz, DMSO-*d$_6$*): δ 188.0, 169.7, 164.0, 152.3, 137.7, 132.8, 129.0, 128.2, 115.3, 63.1, 61.1, 28.8, 27.0, 13.8.
HRMS (ESI-) *m/z*: [M-H]$^-$ Calcd for $C_{16}H_{16}NO_5S^-$ 334.0755; Found, 334.0771.

Ethyl 2-((1-(2-ethoxy-2-oxoethyl)-4-hydroxy-3-(4-nitrobenzoyl)-5-oxo-2,5-dihydro-1*H*-pyrrol-2-yl)thio)acetate (3s)

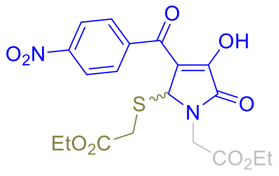

3s

The compound **3s** was prepared from β-enamino diketone **1s** (Ar = 4-$NO_2C_6H_4$, R = CH_2CO_2Et; 0.2 mmol, 1.0 equiv., 0.076 g) in DCM (3 mL), DBU (0.24 mmol, 1.2 equiv, 0.037 g) and ethyl thioglycolate **2a** (0.22 mmol, 1.1 equiv., 0.026 g) were added. The reaction mixture was stirred at room temperature for 10 min, with progress monitored via TLC. Subsequently, the solvent was removed under reduced pressure, and MeOH (3 mL) followed by 10% aqueous HCl (7 mL) were added to the residue. The resulting solid was collected by filtration, washed with cold distilled H_2O (10 mL), and dried in vacuo to give **3s** (0.077 g, 85% yield) as white amorphous solid.

^{1}H NMR (300.06 MHz, DMSO-*d₆*): δ 8.33 (d, J = 8.8 Hz, 2H), 7.97 (d, J = 8.8 Hz, 2H), 5.64 (s, 1H), 4.35 (d, J = 17.7 Hz, 1H), 4.20-4.12 (m, 3H), 4.04-3.96 (m, 2H), 3.23 (d, J = 4.0 Hz, 2H), 1.22 (t, J = 7.1 Hz, 3H), 1.13 (t, J = 7.1 Hz, 3H).
^{13}C{^{1}H} NMR (75.46 MHz, DMSO-d_6): δ 186.6, 169.9, 168.1, 164.3, 153.8, 149.5, 143.1, 130.2, 123.4, 115.6, 62.1, 61.2, 41.7, 29.2, 14.0, 13.9.
HRMS (ESI-) *m/z*: $[M-H]^-$ Calcd for $C_{19}H_{19}N_2O_9S^-$ 451.0817; Found, 451.0836.

5-(Ethylthio)-3-hydroxy-1-methyl-4-(4-nitrobenzoyl)-1,5-dihydro-2*H*-pyrrol-2-one (3t)

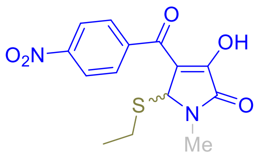

3t

The compound **3t** was prepared from β-enamino diketone **1n** (Ar = 4-$NO_2C_6H_4$, R = Me; 0.2 mmol, 1.0 equiv., 0.061 g) in DCM (3 mL), DBU (0.24 mmol, 1.2 equiv, 0.037 g) and ethanethiol **2b** (0.22 mmol, 1.1 equiv., 0.014 g) were added. The reaction mixture was stirred at room temperature for 10 min, with progress monitored via TLC. Subsequently, the solvent was removed under reduced pressure, and MeOH (3 mL) followed by 10% aqueous HCl (7 mL) were added to the residue. The resulting solid was collected by filtration, washed with cold distilled H_2O (10 mL), and dried in vacuo to give **3t** (0.054 g, 84% yield) as light yellow amorphous solid.

^{1}H NMR (300.06 MHz, DMSO-d_6): δ 8.32 (d, J = 8.6 Hz, 2H), 7.95 (d, J = 8.6 Hz, 2H), 5.53 (s, 1H), 3.00 (s, 3H), 2.34-2.27 (m, 2H), 1.11 (t, J = 7.4 Hz, 3H).
^{13}C{^{1}H} NMR (75.46 MHz, DMSO-d_6): δ 186.8, 163.8, 153.6, 149.5, 143.2, 130.0, 123.5, 115.9, 62.8, 27.1, 20.9, 14.7.
HRMS (ESI-) *m/z*: $[M-H]^-$ Calcd for $C_{14}H_{13}N_2O_5S^-$ 321.0551; Found, 321.0567.

Ethyl 2-(2-(ethylthio)-4-hydroxy-3-(4-nitrobenzoyl)-5-oxo-2,5-dihydro-1*H*-pyrrol-1-yl)acetate (3u)

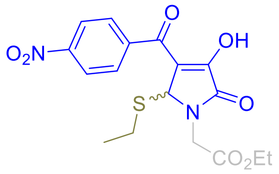

3u

The compound **3u** was prepared from β-enamino diketone **1s** (Ar = 4-$NO_2C_6H_4$, R = CH_2CO_2Et; 0.2 mmol, 1.0 equiv., 0.076 g) in DCM (3 mL), DBU (0.24 mmol, 1.2 equiv, 0.037 g) and ethanethiol **2b** (0.22 mmol, 1.1 equiv., 0.014 g) were added. The reaction mixture was stirred at room temperature for 10 min, with progress monitored via TLC. Subsequently, the solvent was removed under reduced pressure, and MeOH (3 mL) followed by 10% aqueous HCl (7 mL) were added to the residue. The resulting solid was collected by filtration, washed with cold distilled H_2O (10 mL), and dried in vacuo to give **3u** (0.067 g, 85% yield) as light yellow amorphous solid.

^{1}H NMR (500.13 MHz, DMSO-*d₆*): δ 8.33 (d, *J* = 8.8 Hz, 2H), 7.98 (d, *J* = 8.8 Hz, 2H), 5.58 (s, 1H), 4.42 (d, *J* = 17.6 Hz, 1H), 4.21 (d, *J* = 17.6 Hz, 1H), 4.16 (qd, *J* = 7.1, 1.5 Hz, 2H), 2.34 (q, *J* = 7.5 Hz, 2H), 1.22 (t, *J* = 7.1 Hz, 3H), 1.06 (t, *J* = 7.1 Hz, 3H).
^{13}C{^{1}H} NMR (125.77 MHz, DMSO-*d₆*): δ 186.9, 168.1, 164.2, 152.9, 149.6, 142.9, 130.1, 123.6, 116.6, 62.0, 61.2, 41.8, 20.8, 14.3, 14.0.
HRMS (ESI-) *m/z*: [M-H]$^-$ Calcd for $C_{17}H_{17}N_2O_7S^-$ 393.0762; Found, 393.0780.

5-(Cyclohexylthio)-3-hydroxy-1-methyl-4-(4-nitrobenzoyl)-1,5-dihydro-2*H*-pyrrol-2-one (3v)

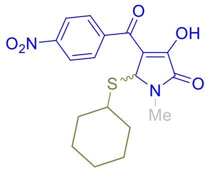

3v

The compound **3v** was prepared from β-enamino diketone **1n** (Ar = 4-$NO_2C_6H_4$, R = Me; 0.2 mmol, 1.0 equiv., 0.061 g) in DCM (3 mL), DBU (0.24 mmol, 1.2 equiv, 0.037 g) and cyclohexanethiol **2c** (0.22 mmol, 1.1 equiv., 0.026 g) were added. The reaction mixture was stirred at room temperature for 10 min, with progress monitored via TLC. Subsequently, the solvent was removed under reduced pressure, and MeOH (3 mL) followed by 10% aqueous HCl (7 mL) were added to the residue. The resulting solid was collected by filtration, washed with cold distilled H_2O (10 mL), and dried in vacuo to give **3v** (0.067 g, 90% yield) as white amorphous solid.

^{1}H NMR (300.06 MHz, DMSO-*d₆*): δ 8.34 (d, *J* = 8.8 Hz, 2H), 7.93 (d, *J* = 8.8 Hz, 2H), 5.56 (s, 1H), 3.00 (s, 3H), 2.69-2.61 (m, 1H), 1.83-1.74 (m, 2H), 1.63-1.56 (m, 2H), 1.50-1.45 (m, 1H), 1.36-1.16 (m, 5H).
^{13}C{^{1}H} NMR (75.46 MHz, DMSO-*d₆*): δ 186.8, 163.7, 153.3, 149.5, 143.1, 130.0, 123.6, 116.4, 63.0, 41.5, 34.3, 34.1, 27.1, 25.3, 25.3, 25.0.
HRMS (ESI-) *m/z*: [M-H]$^-$ Calcd for $C_{18}H_{19}N_2O_5S^-$ 375.1020; Found, 375.1025.

Ethyl 2-(2-(cyclohexylthio)-4-hydroxy-3-(4-nitrobenzoyl)-5-oxo-2,5-dihydro-1*H*-pyrrol-1-yl)acetate (3w)

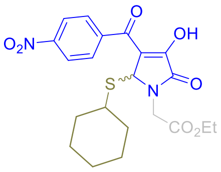

3w

The compound **3w** was prepared from β-enamino diketone **1s** (Ar = 4-$NO_2C_6H_4$, R = CH_2CO_2Et; 0.2 mmol, 1.0 equiv., 0.076 g) in DCM (3 mL), DBU (0.24 mmol, 1.2 equiv, 0.037 g) and cyclohexanethiol **2c** (0.22 mmol, 1.1 equiv., 0.026 g) were added. The reaction mixture was stirred at room temperature for 10 min, with progress monitored via TLC. Subsequently, the solvent was removed under reduced pressure, and MeOH (3 mL) followed by 10% aqueous HCl (7 mL) were added to the residue. The resulting solid was collected by filtration, washed with cold distilled H_2O (10 mL), and dried in vacuo to give **3w** (0.075 g, 83% yield) as light yellow amorphous solid.

^{1}H NMR (300.06 MHz, DMSO-*d*$_6$): δ 8.35 (d, *J* = 8.9 Hz, 2H), 7.97 (d, *J* = 8.9 Hz, 2H), 5.59 (s, 1H), 4.47 (d, *J* = 17.6 Hz, 1H), 4.22-4.14 (m, 3H), 2.71-2.63 (m, 1H), 1.78-1.70 (m, 2H), 1.59-1.43 (m, 3H), 1.32-1.15 (m, 8H).
^{13}C{^{1}H} NMR (75.46 MHz, DMSO-*d*$_6$): δ 186.9, 168.1, 164.2, 152.7, 149.6, 142.8, 130.1, 123.6, 117.2, 62.1, 61.2, 41.6, 41.4, 34.4, 33.9, 25.4, 25.2, 25.0, 14.0.
HRMS (ESI-) *m/z*: $[M-H]^-$ Calcd for $C_{21}H_{23}N_2O_7S^-$ 447.1231; Found, 447.1239.

CONTROL EXPERIMENTS

Radical scavenger experiment

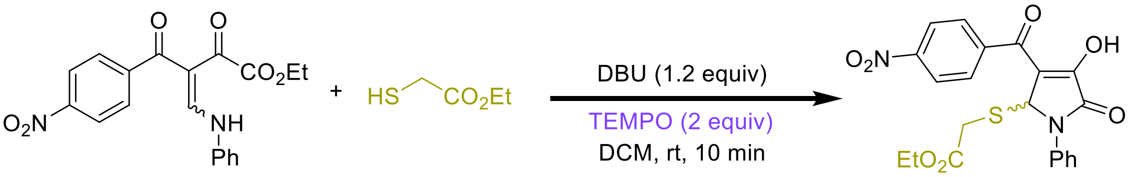

1a **3a**, 83%

The radical scavenger experiment was carried out by adding TEMPO (2,2,6,6-tetramethylpiperidin-1-yl)oxyl, 2.0 equiv.) to the standard reaction mixture under the optimized conditions.
To a solution of **1a** (0.2 mmol, 1.0 equiv., 0.074 g), DBU (0.24 mmol, 1.2 equiv, 0.037 g), radical scavenger TEMPO (0.40 mmol, 2 equiv., 0.063 g) in DCM (3 mL), ethylthioglicolate **2a** (0.22 mmol, 1.1 equiv., 0.026 g) was added. The resulting solution was allowed to stir at room temperature for 10 minutes. After this, the solvent was removed under reduced pressure, and MeOH (3 mL) followed by 10% aqueous HCl (7 mL) were added to the residue. The resulting solid was collected by filtration, washed with cold distilled H_2O (10 mL), and dried in vacuo to give **3a** (0.073 g, 83% yield) as white amorphous solid.

Investigation of DBU role

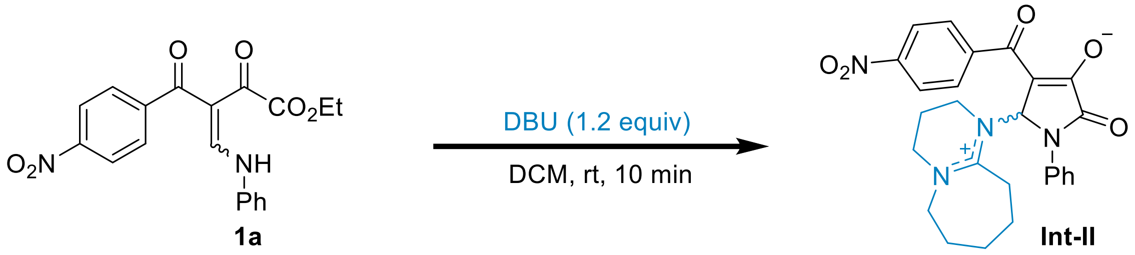

A solution of **1a** (0.2 mmol, 1.0 equiv., 0.074 g) and DBU (0.24 mmol, 1.2 equiv, 0.037 g) in DCM was allowed to stir at room temperature for 10 minutes. After this, the solvent was removed under reduced pressure, and the residue was analyzed by NMR (Figure S2).

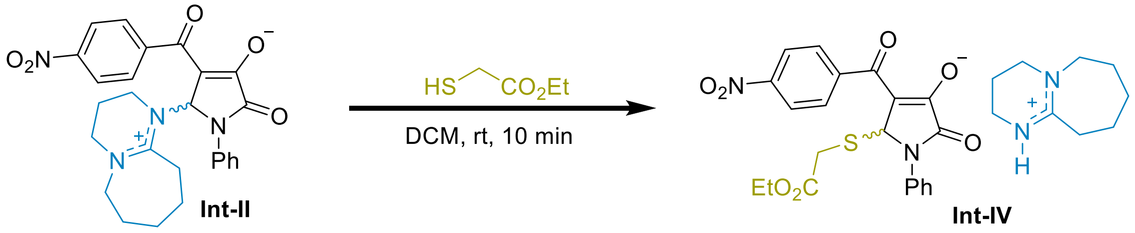

To a solution of **Int-II** in DCM, ethyl thioglycolate (0.22 mmol, 1.1 equiv., 0.026 g) was added and allowed to stir at room temperature for 10 minutes. After this, the solvent was removed under reduced pressure, and the residue was analyzed by NMR (Figure S4).

Note: The intermediates **Int-II** and **Int-IV** proved difficult to isolate in pure form.

Structural Characterization of Int-II and Int-IV

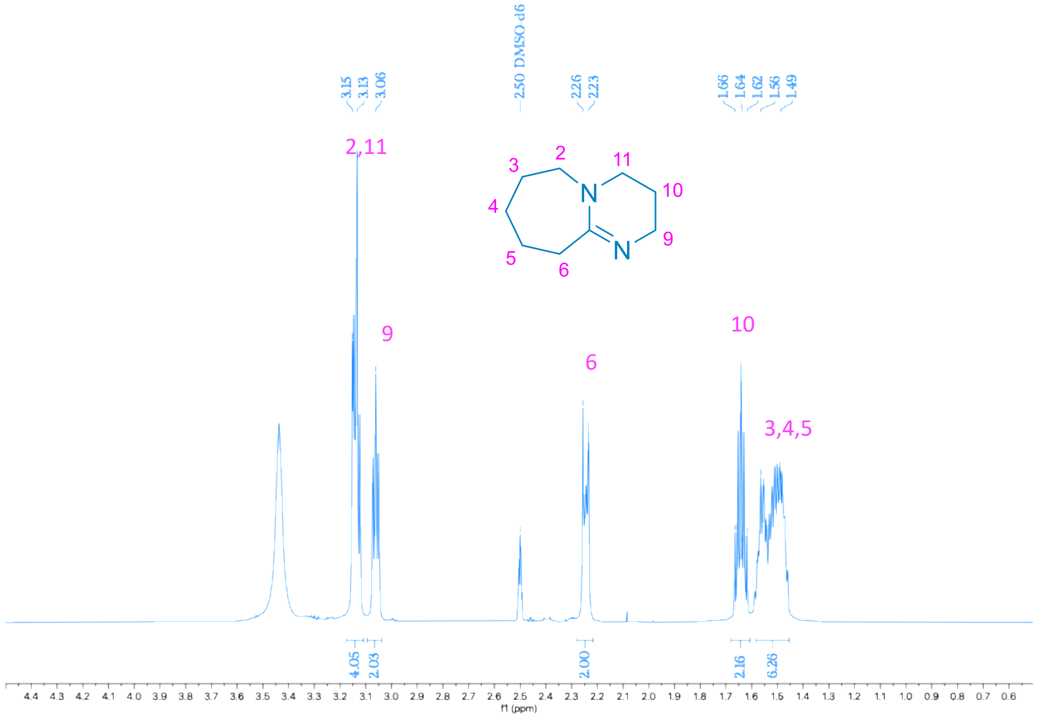

Figure S1. ^{1}H NMR spectrum of DBU (DMSO-d_6, 500 MHz)

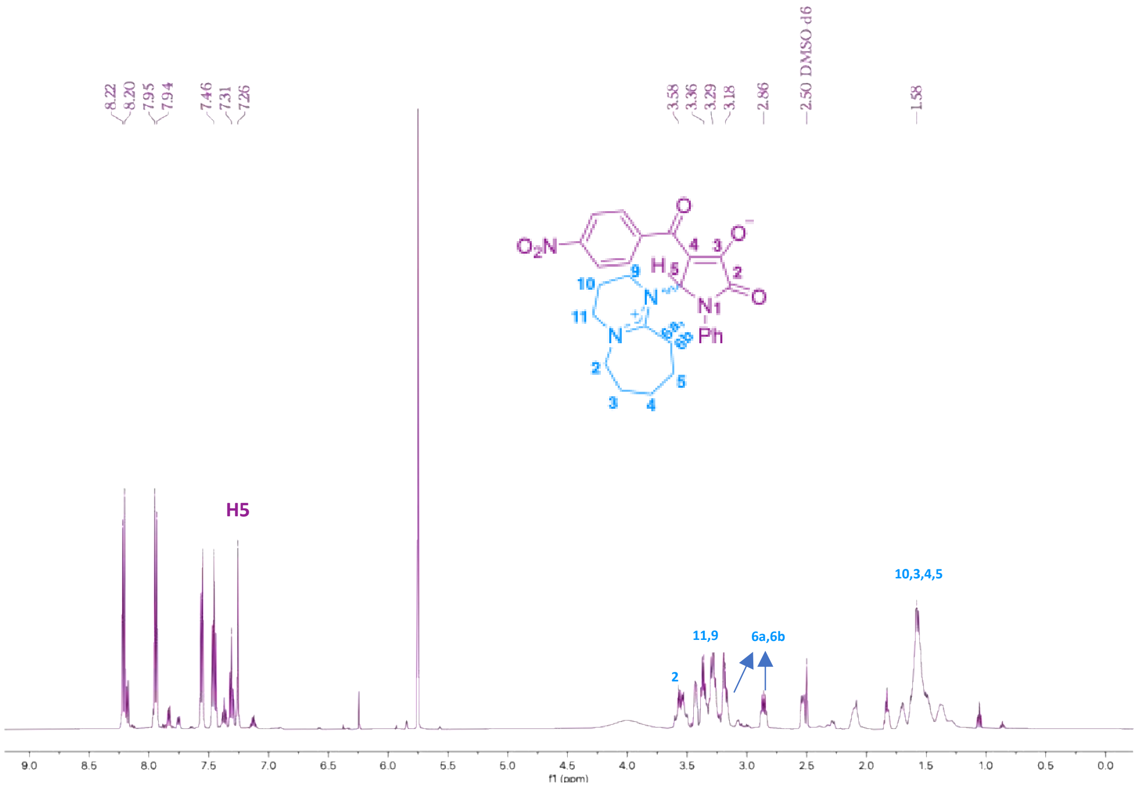

Figure S2. ^{1}H NMR spectrum of **Int-II** (DMSO-d_6, 500 MHz)

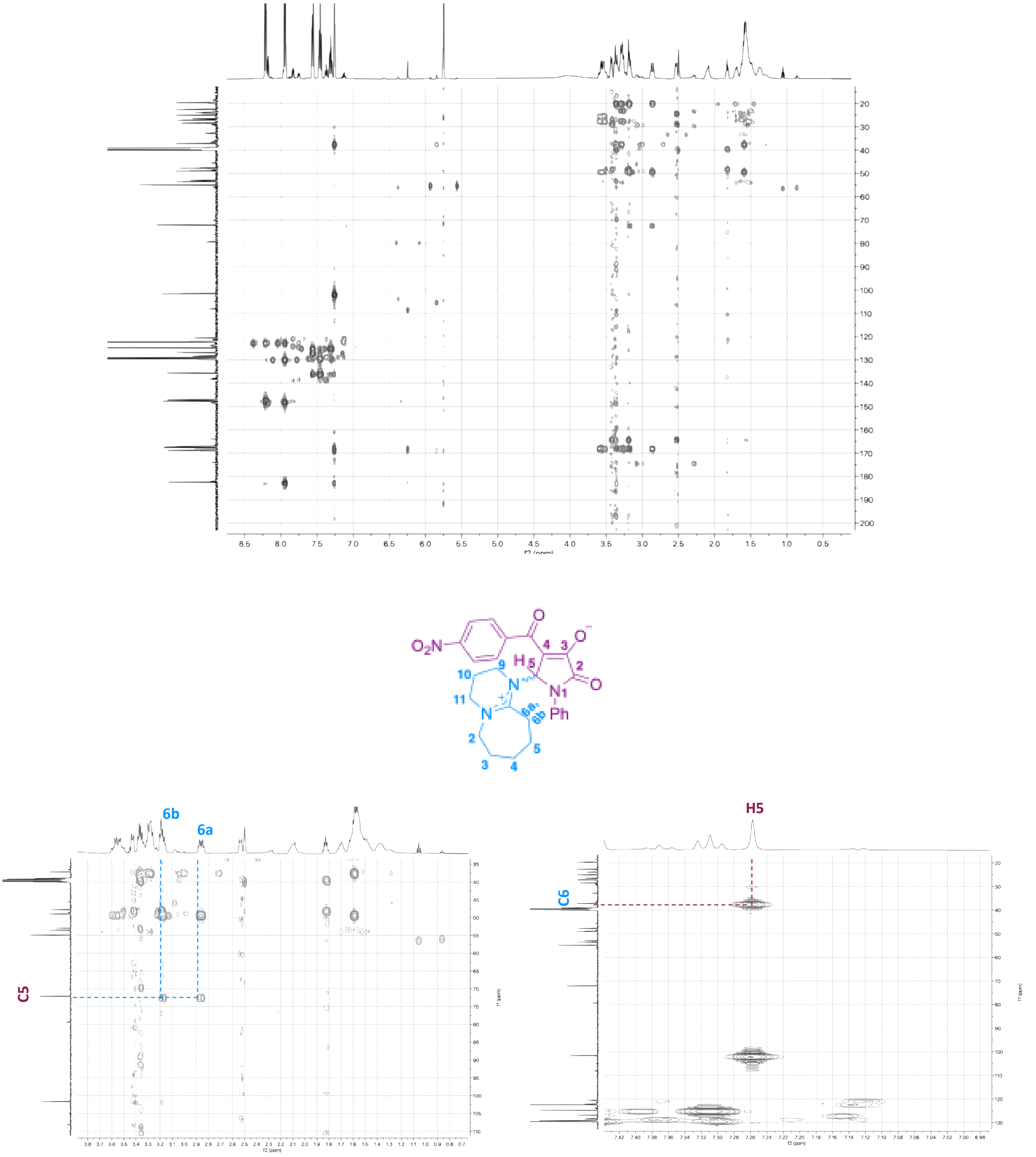

Figure S3. HMBC NMR analysis of **Int-II** (DMSO-d_6, 500 MHz)

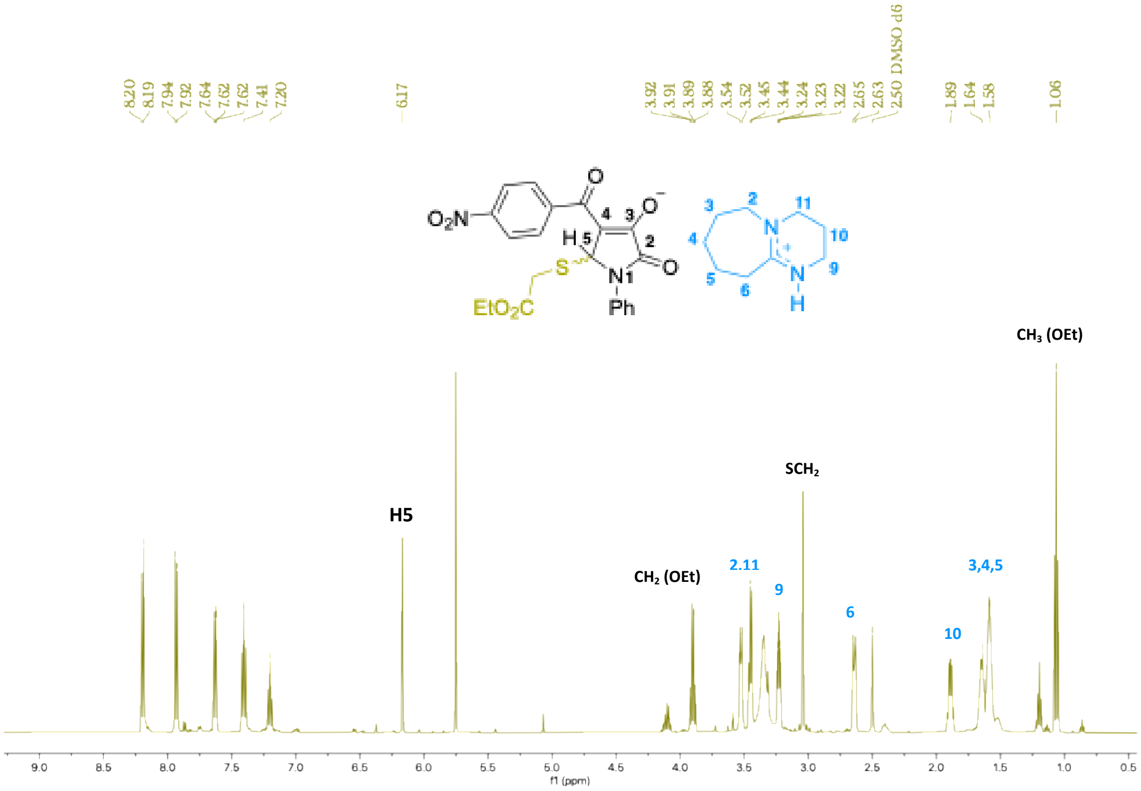

Figure S4. ¹H NMR spectrum of **Int-IV** (DMSO-d_6, 500 MHz)

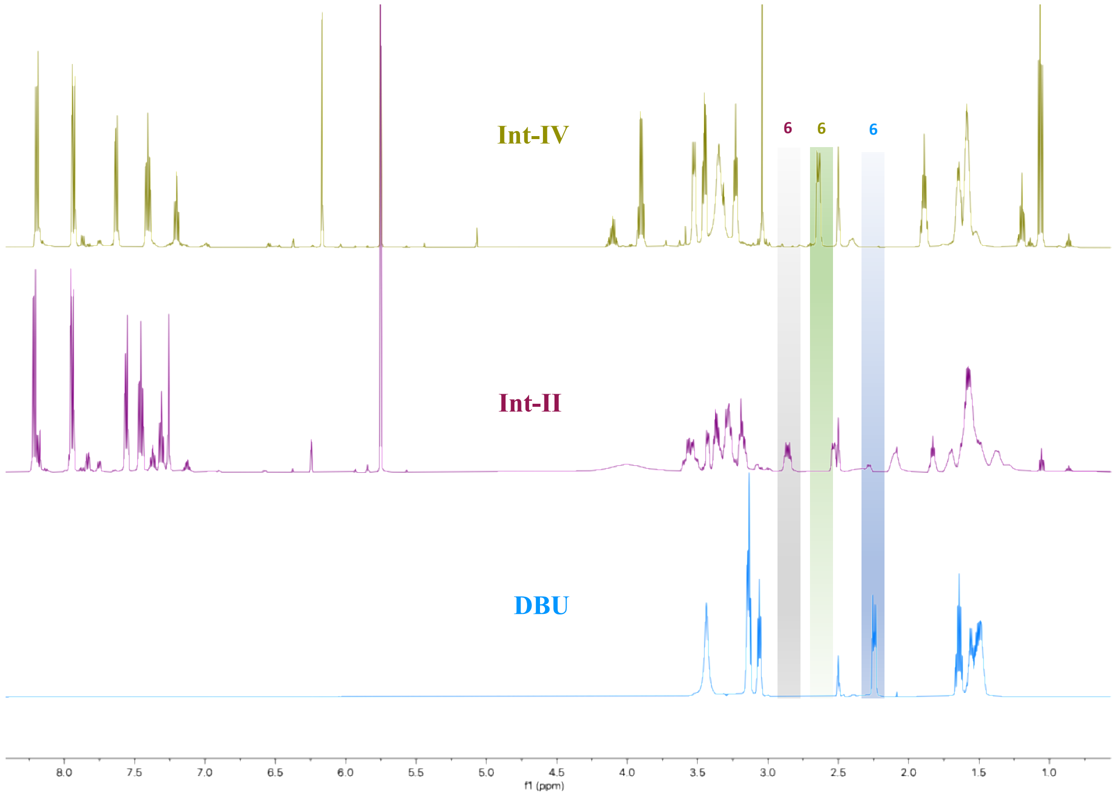

Figure S5. Comparison the ¹H NMR spectra of DBU (blue), **Int-II** (purple), and **Int-IV** (olive), emphasizing the changes in proton chemical shifts (DMSO-d_6, 500 MHz)

3. ^{1}H and ^{13}C{^{1}H} NMR Spectra for the 5-Sulfenylated 3-hydroxy-γ-lactams

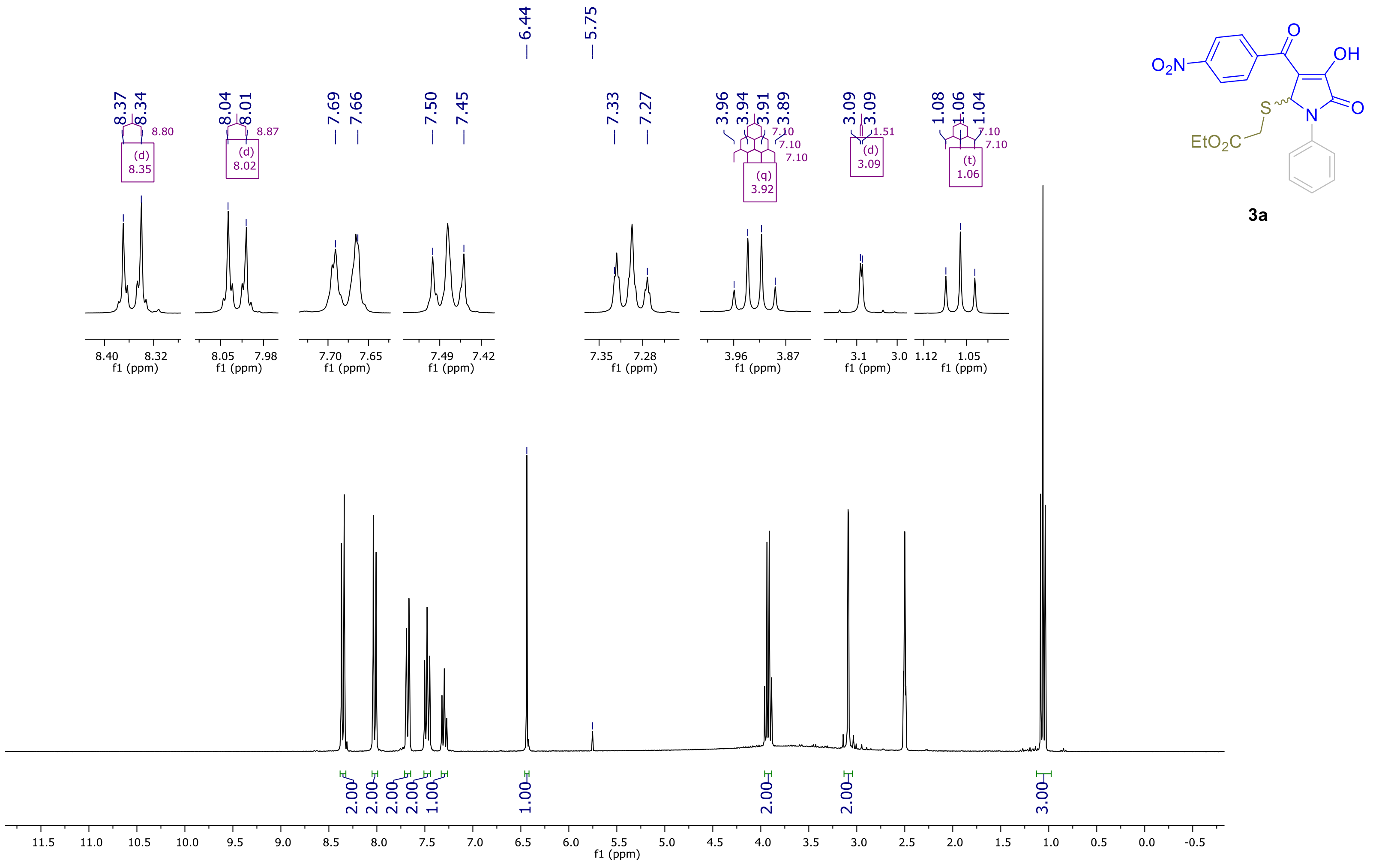

Figure S6. ^{1}H NMR Spectrum (300.06 MHz, DMSO-d$_6$) of compound **3a**

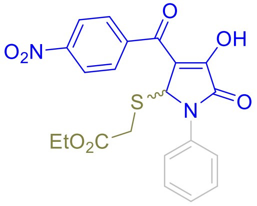

3a

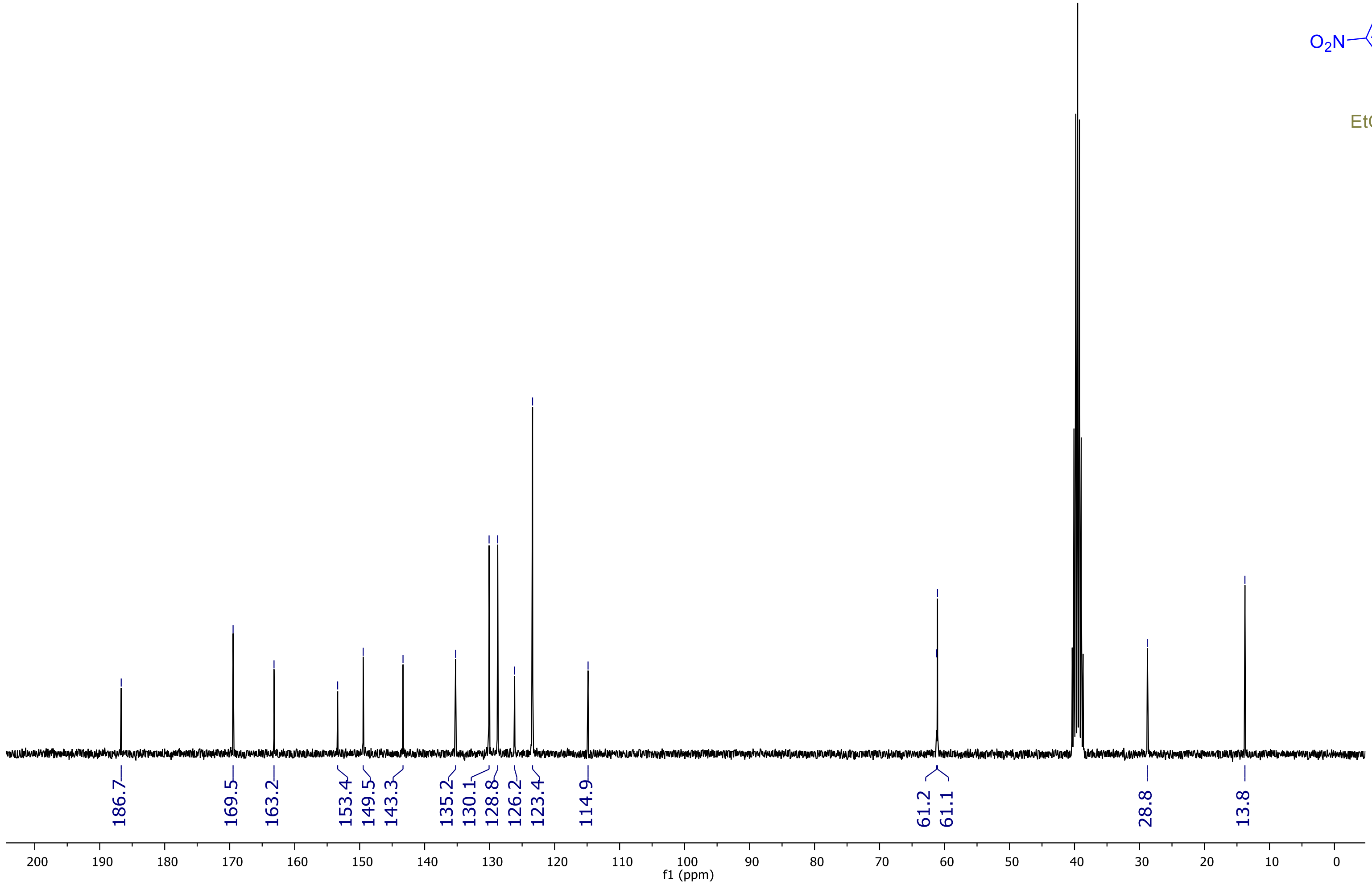

Figure S7. $^{13}C\{^{1}H\}$ NMR Spectrum (75.46 MHz, DMSO-d_6) of compound **3a**

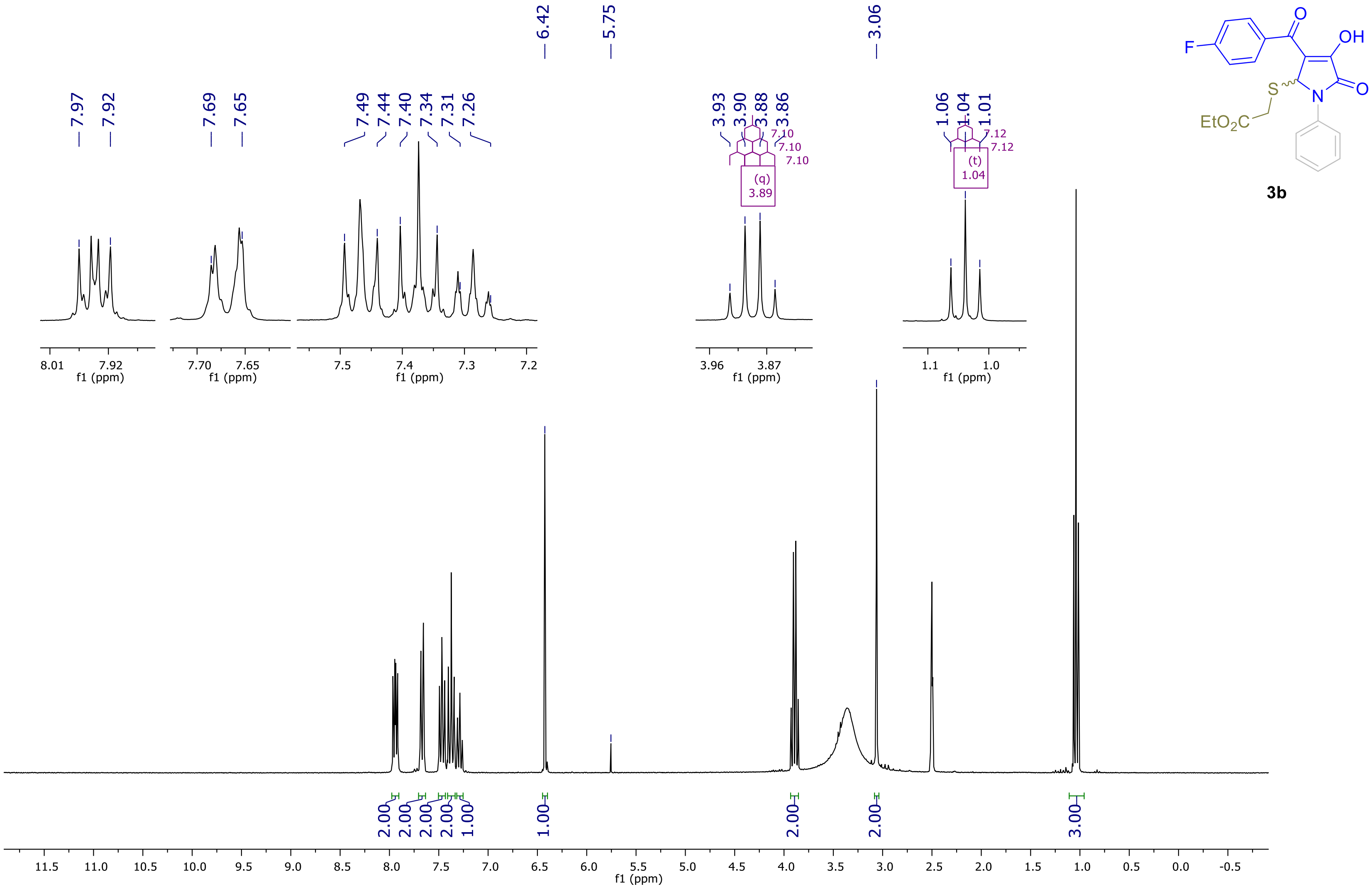

Figure S8. ^{1}H NMR Spectrum (300.06 MHz, DMSO-d$_6$) of compound **3b**

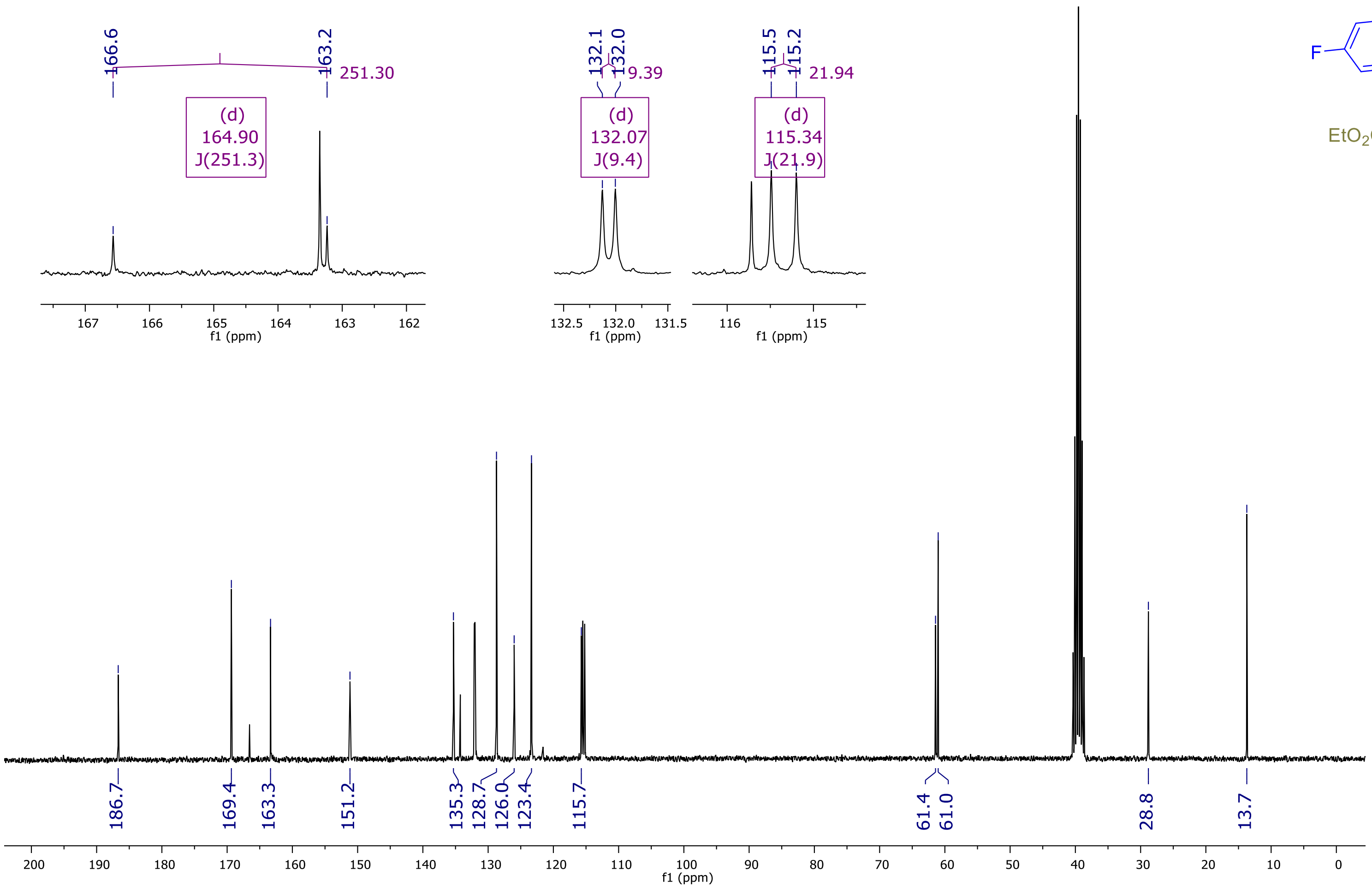

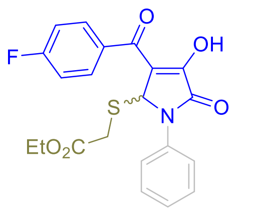

3b

Figure S9. $^{13}C\{^1H\}$ NMR Spectrum (75.46 MHz, DMSO-d_6) of compound **3b**

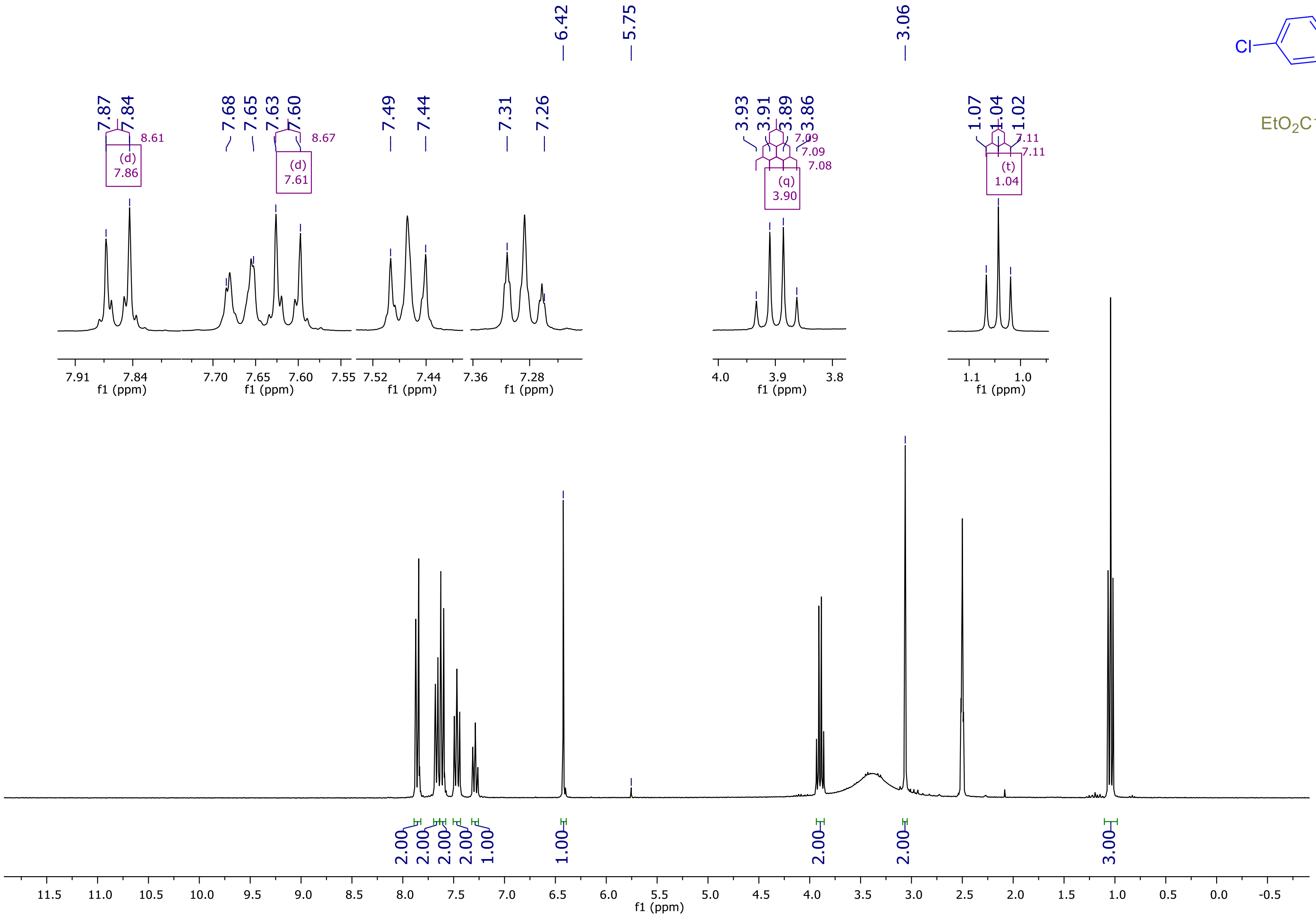

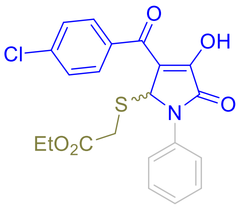

3c

Figure S10. ^{1}H NMR Spectrum (300.06 MHz, DMSO-d_6) of compound **3c**

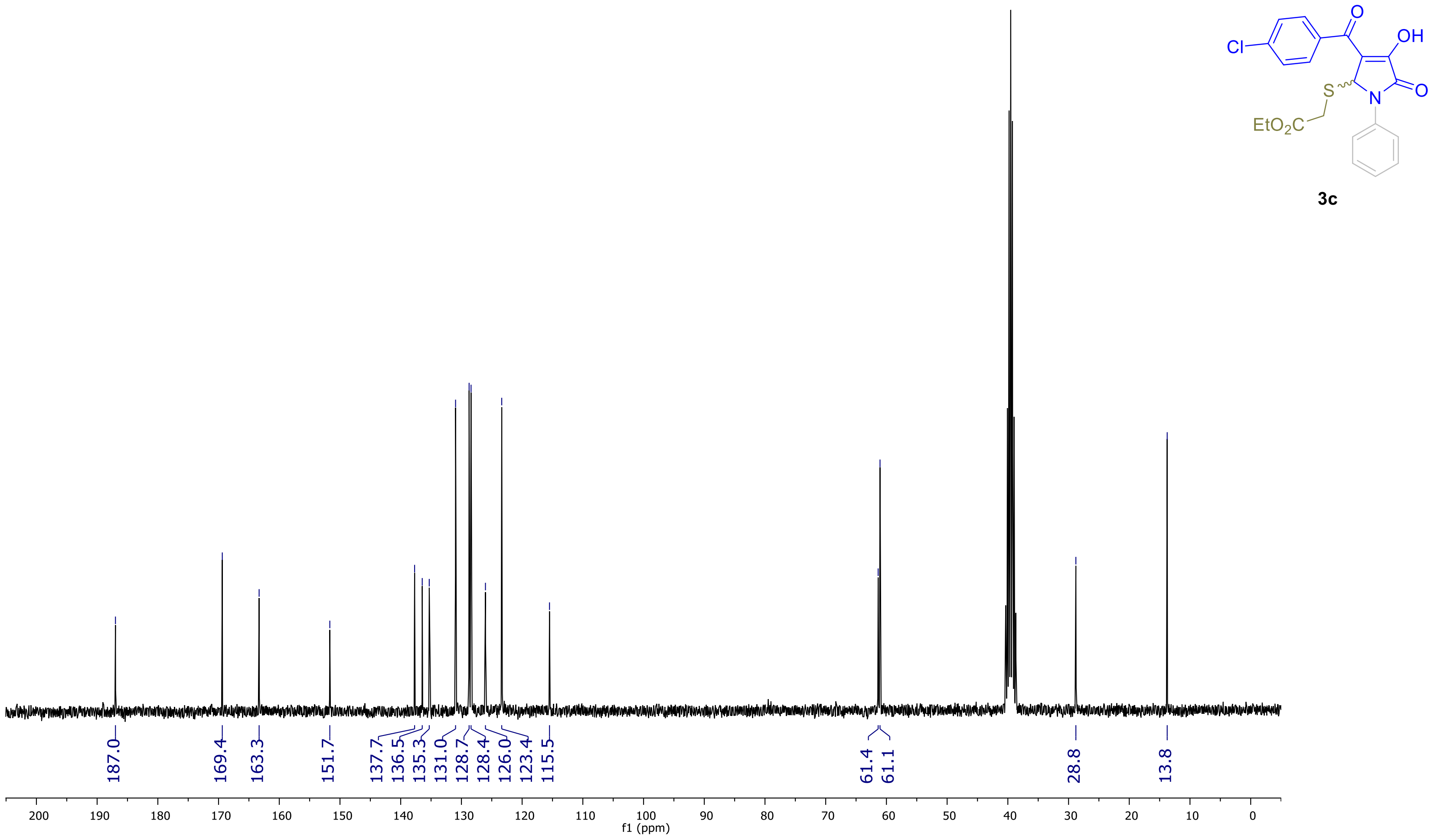

Figure S11. $^{13}C\{^{1}H\}$ NMR Spectrum (75.46 MHz, DMSO-d_6) of compound **3c**

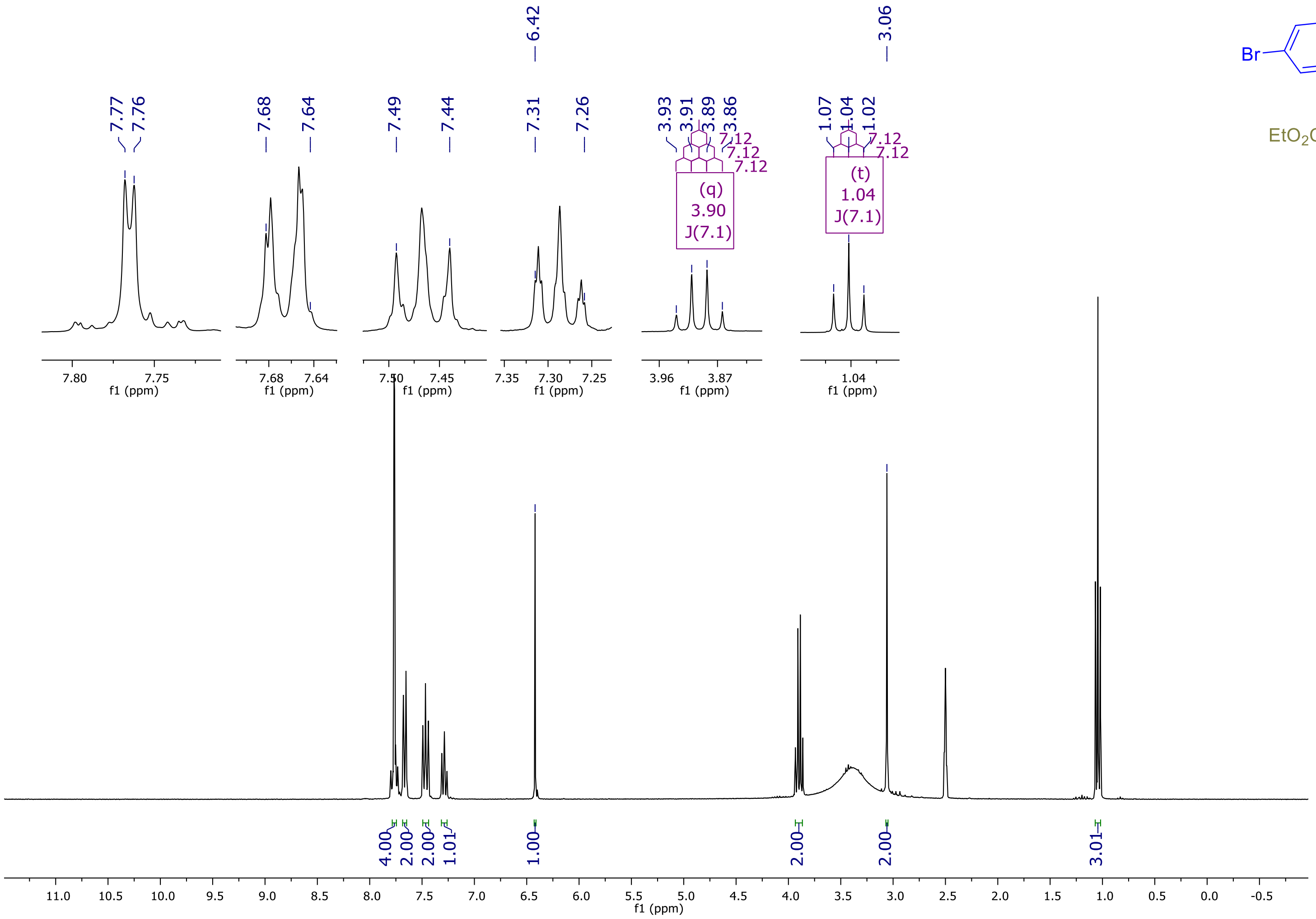

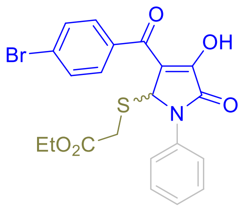

3d

Figure S12. ^{1}H NMR Spectrum (300.06 MHz, DMSO-d$_6$) of compound **3d**

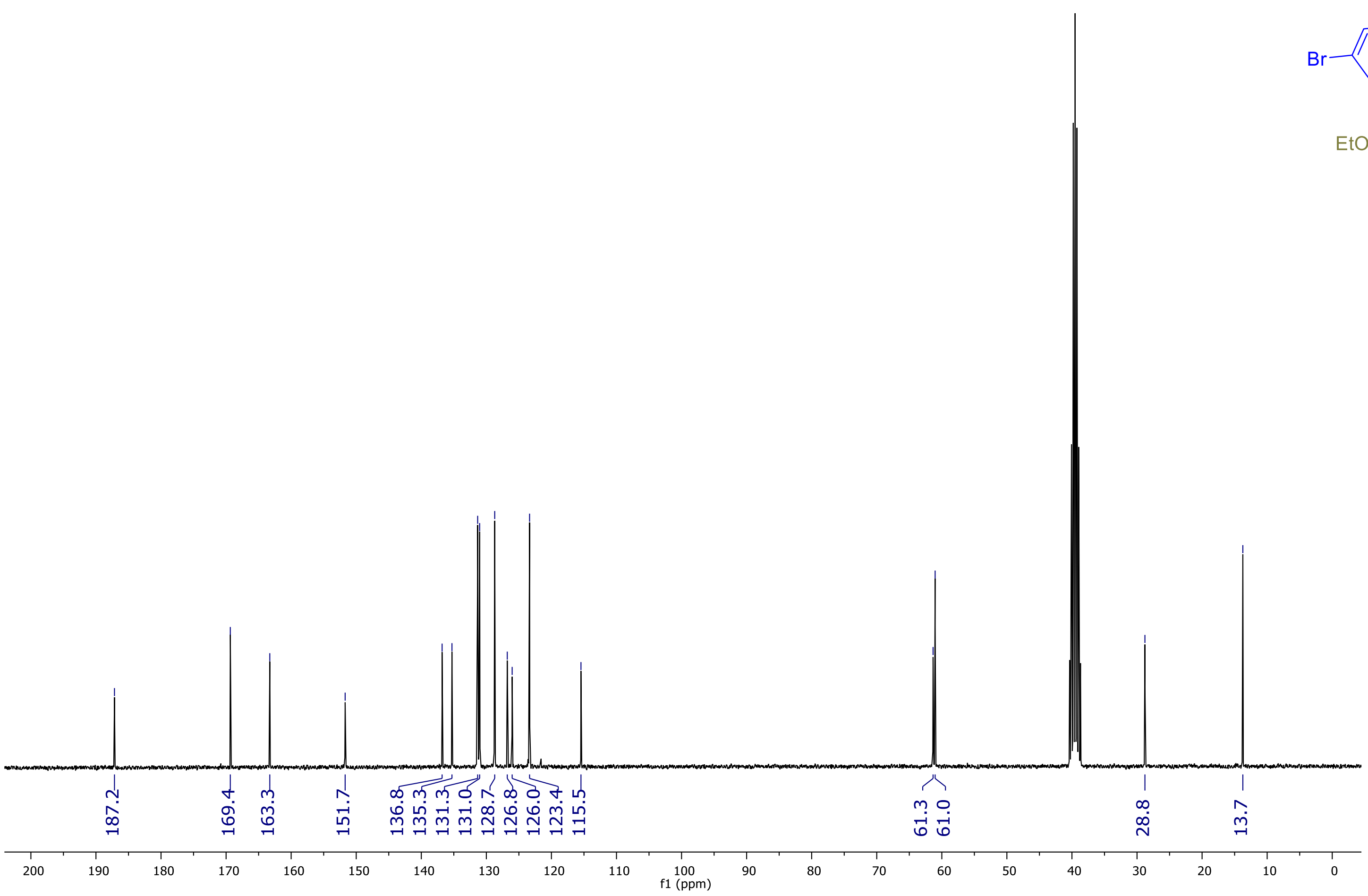

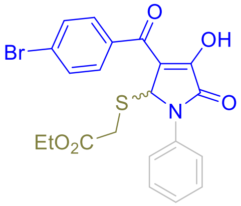

3d

Figure S13. $^{13}C\{^1H\}$ NMR Spectrum (75.46 MHz, DMSO-d_6) of compound **3d**

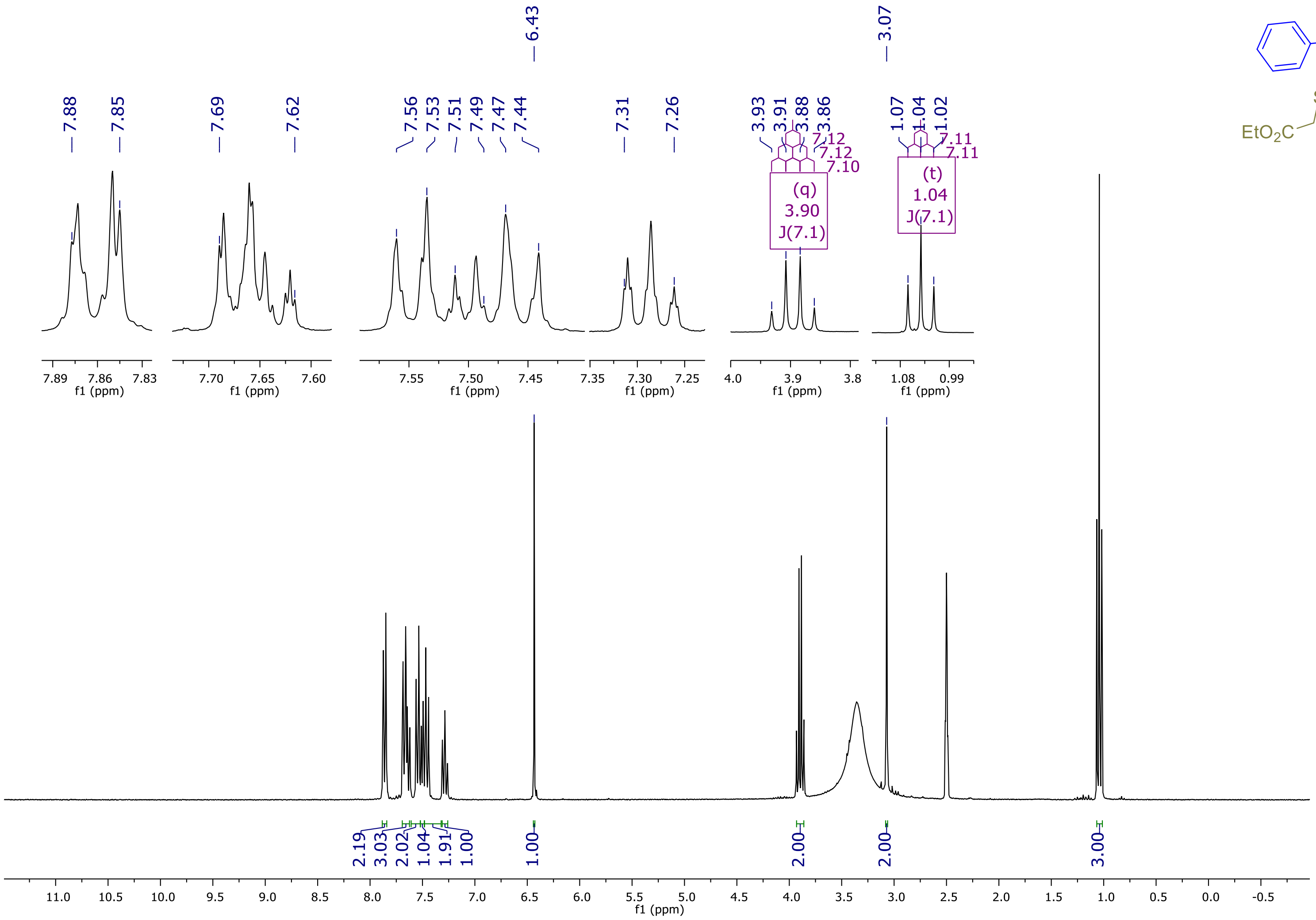

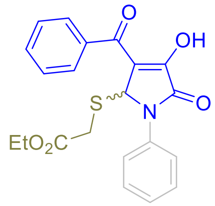

3e

Figure S14. ^{1}H NMR Spectrum (300.06 MHz, DMSO-d$_6$) of compound **3e**

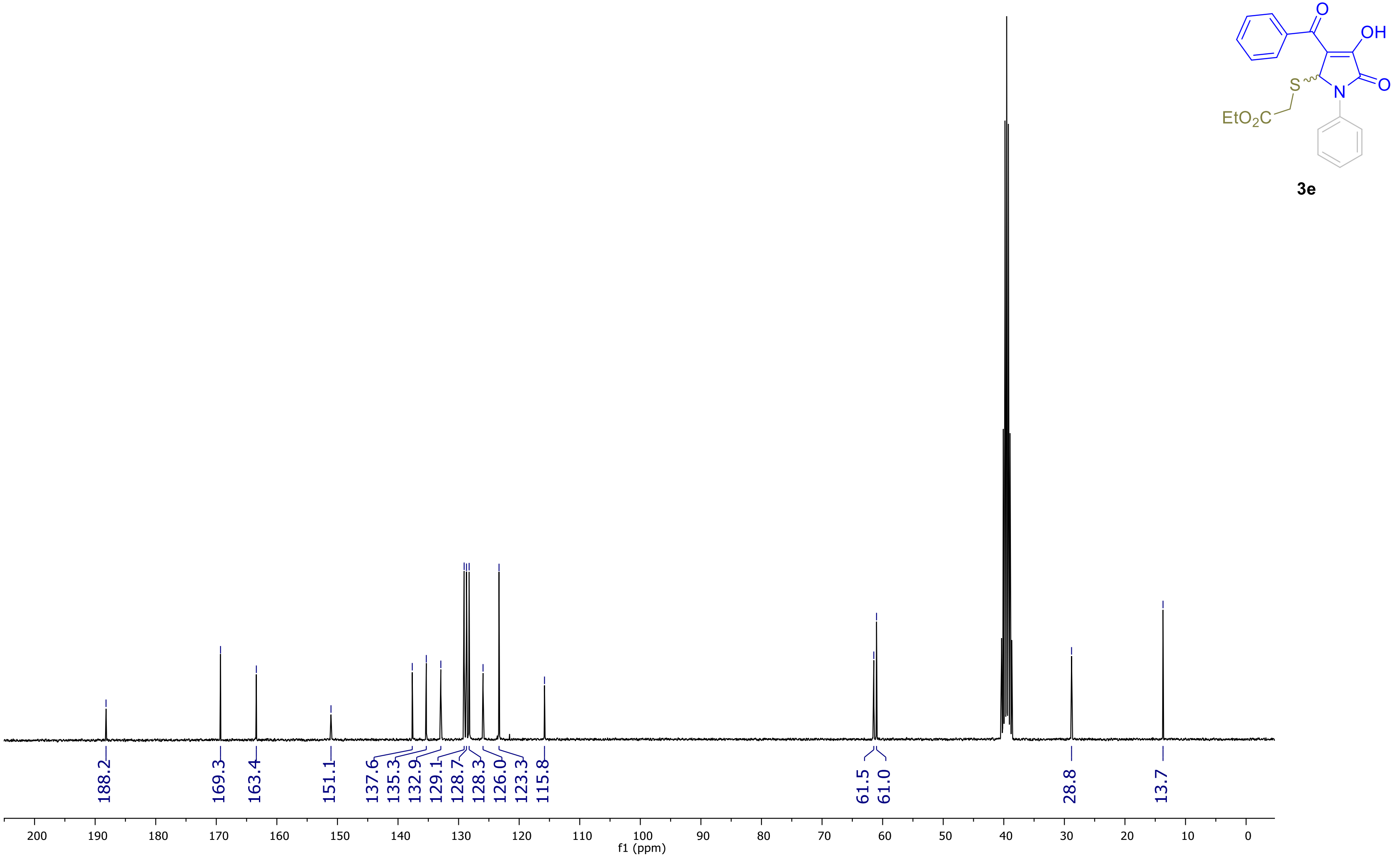

Figure S15. $^{13}C\{^1H\}$ NMR Spectrum (75.46 MHz, DMSO-d_6) of compound **3e**

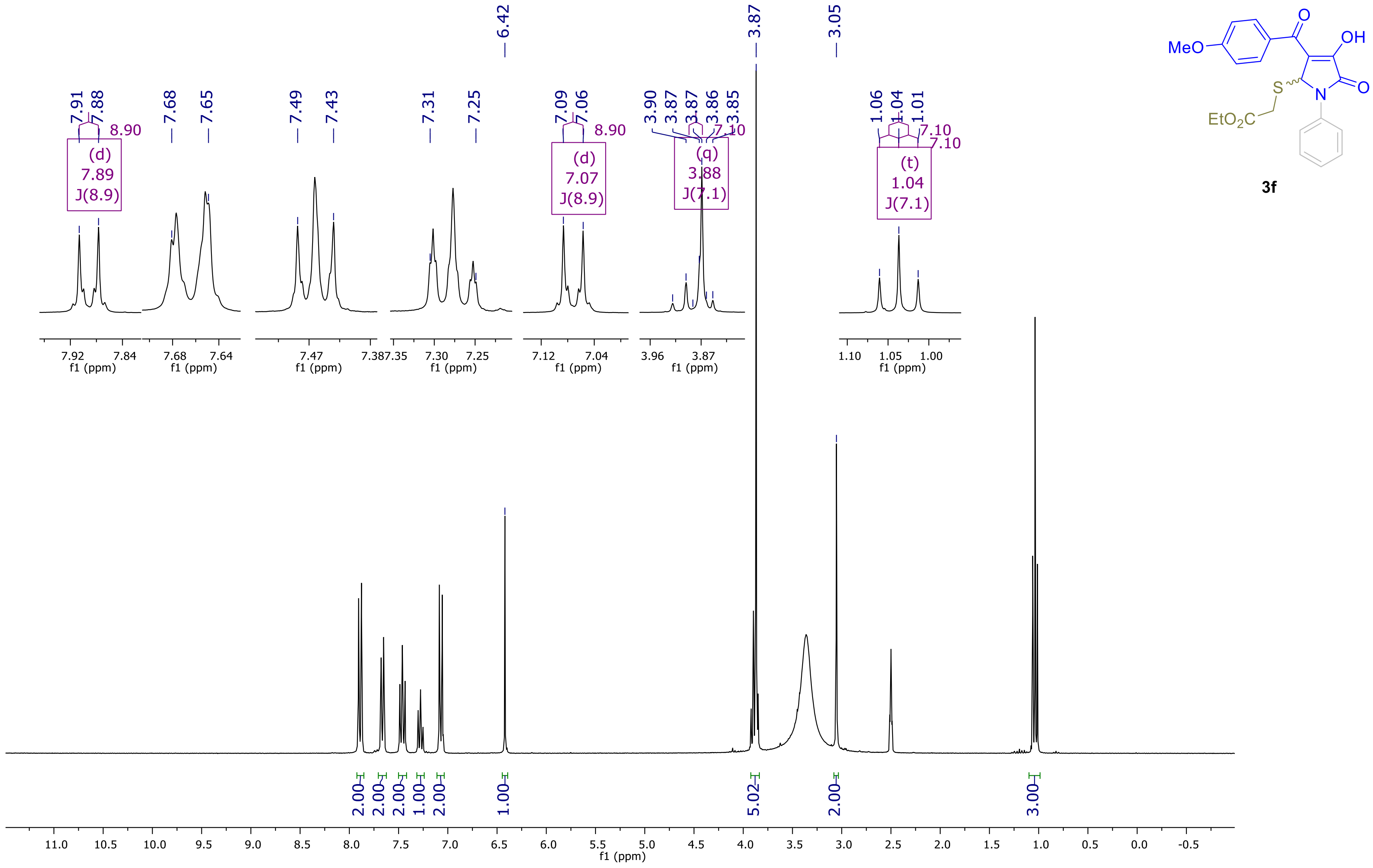

3f

Figure S16. ^{1}H NMR Spectrum (300.06 MHz, DMSO-d_6) of compound **3f**

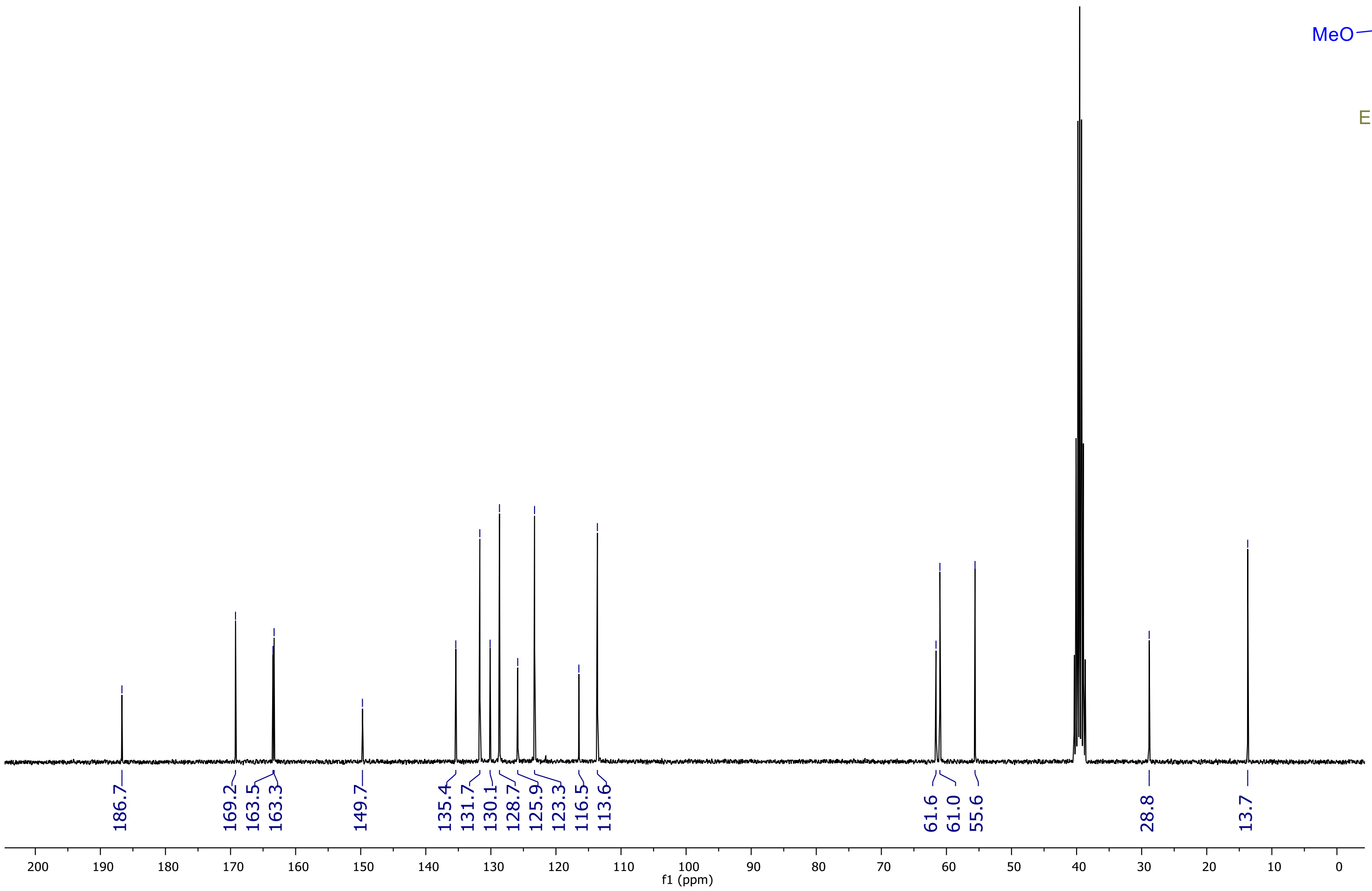

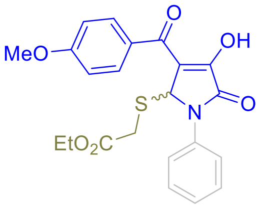

3f

Figure S17. $^{13}C\{^1H\}$ NMR Spectrum (75.46 MHz, DMSO-d_6) of compound **3f**

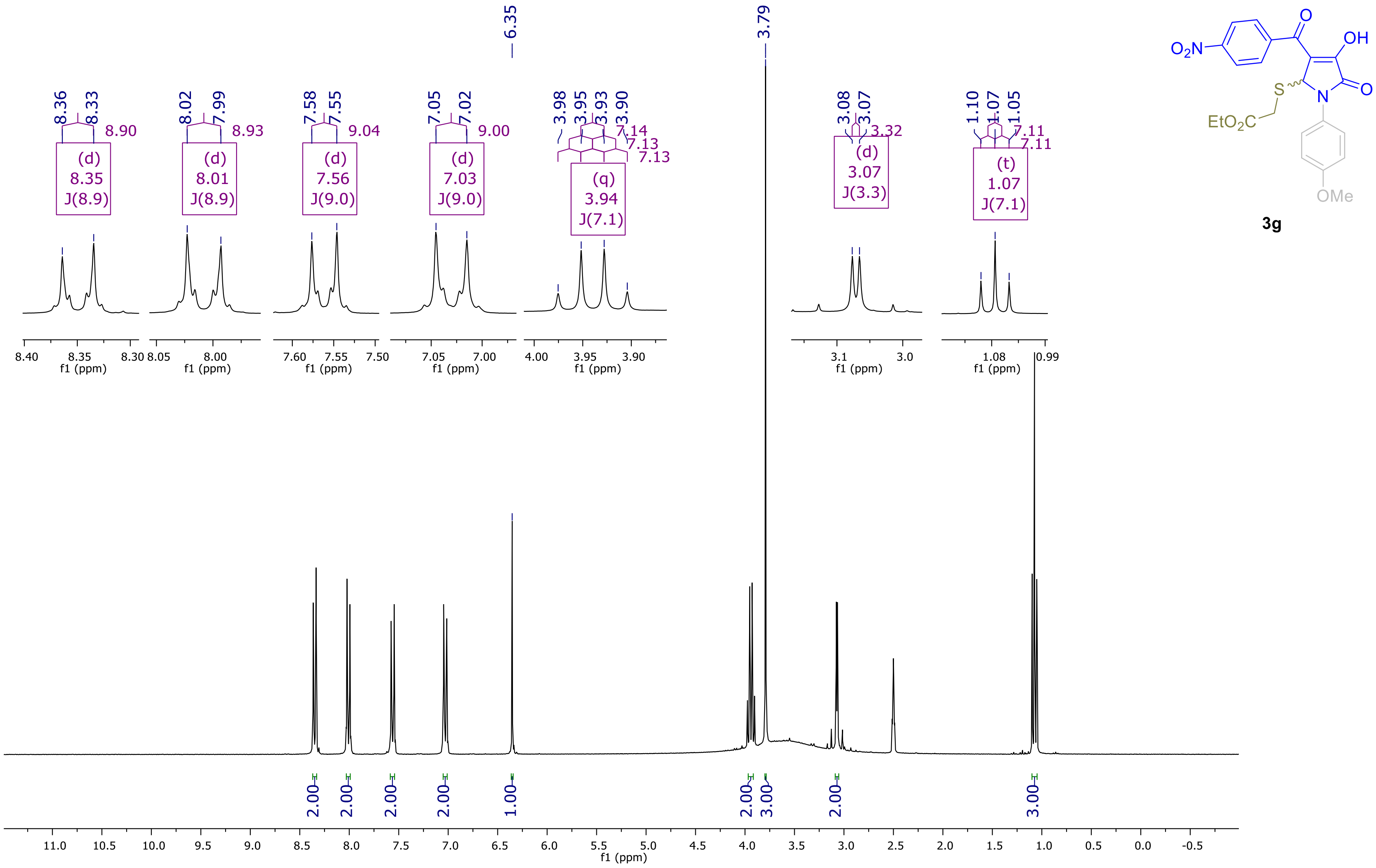

Figure S18. ^{1}H NMR Spectrum (300.06 MHz, DMSO-d_6) of compound **3g**

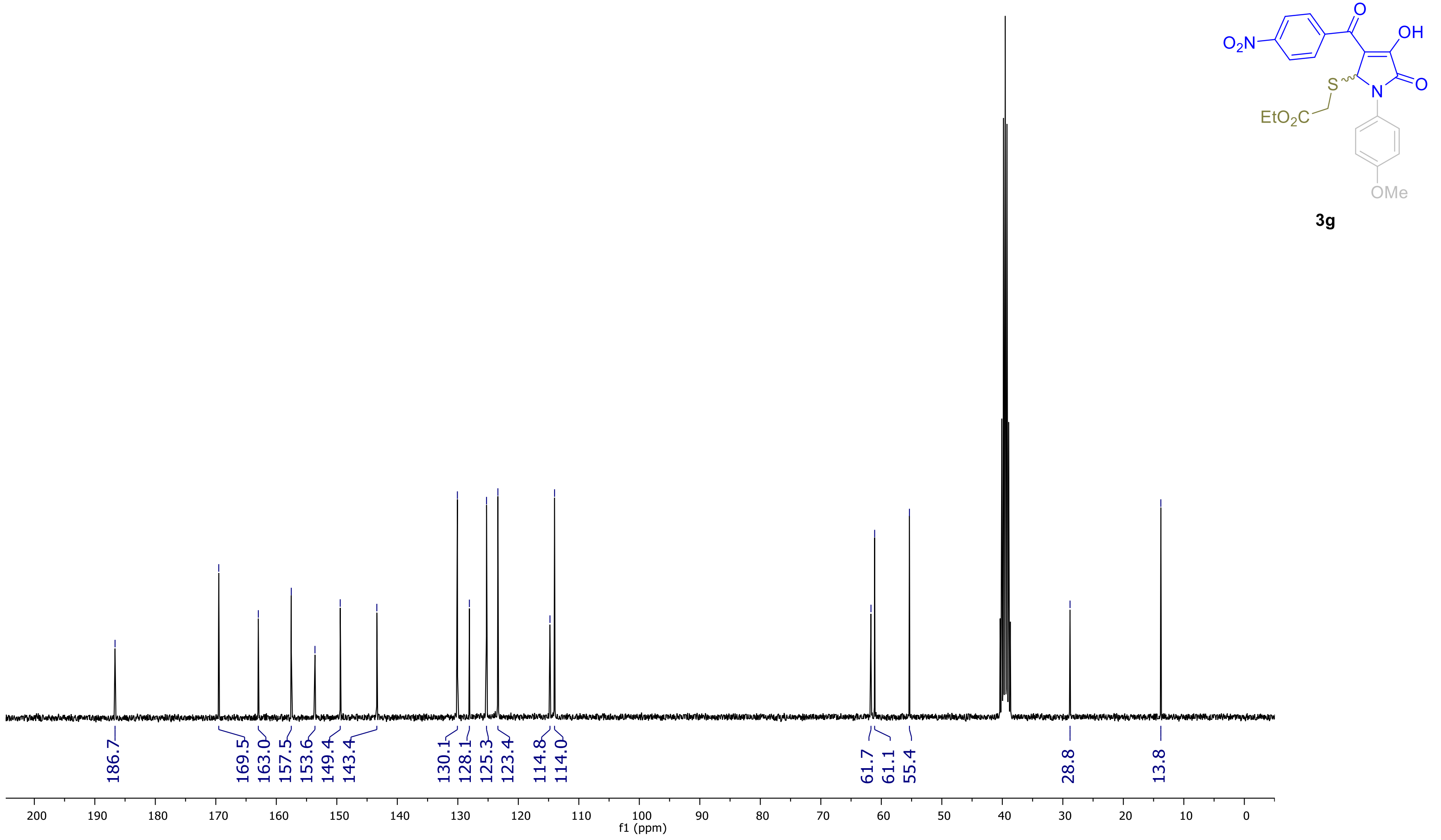

3g

Figure S19. $^{13}C\{^1H\}$ NMR Spectrum (75.46 MHz, DMSO-d_6) of compound **3g**

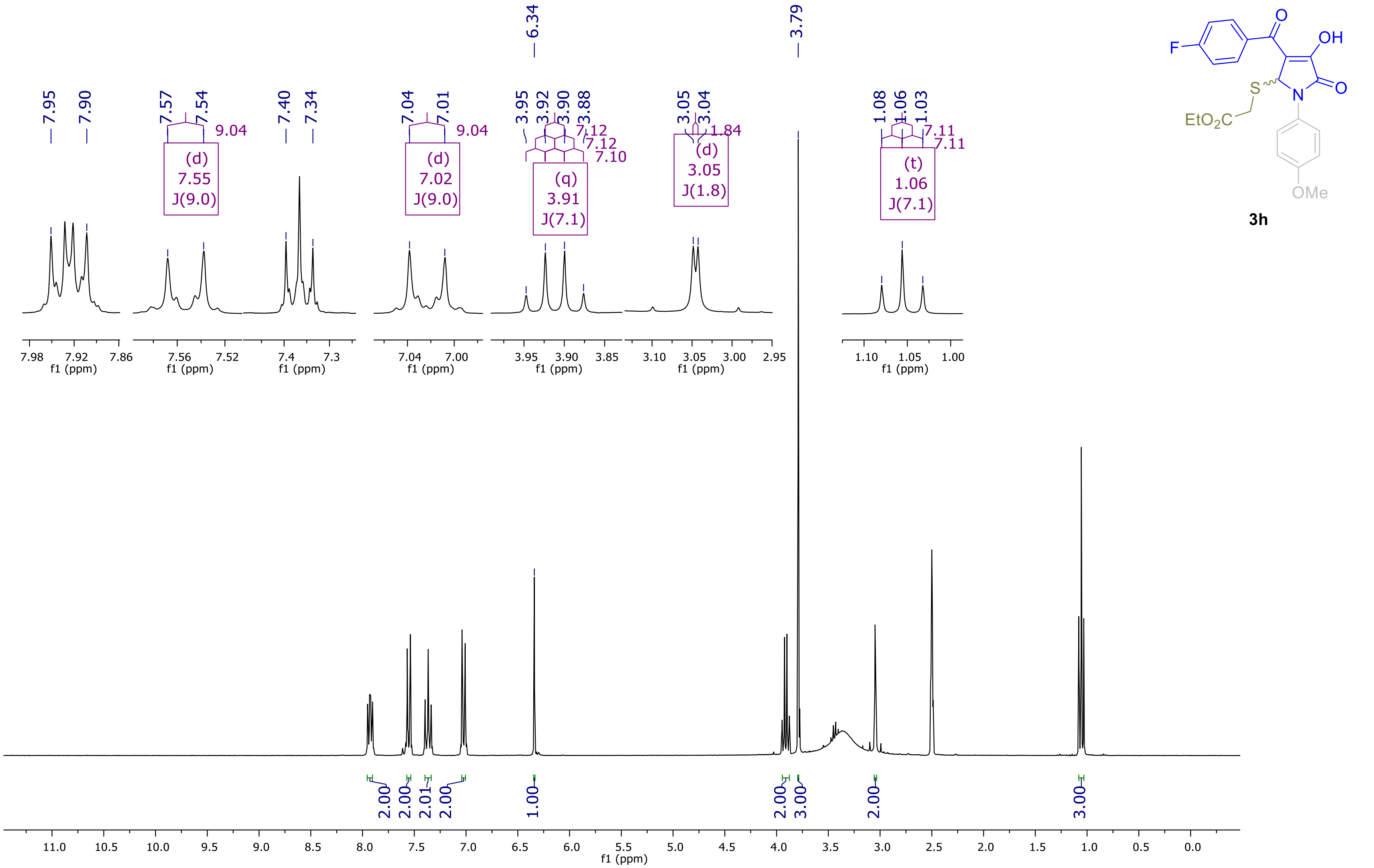

Figure S20. ^{1}H NMR Spectrum (300.06 MHz, DMSO-d_6) of compound **3h**

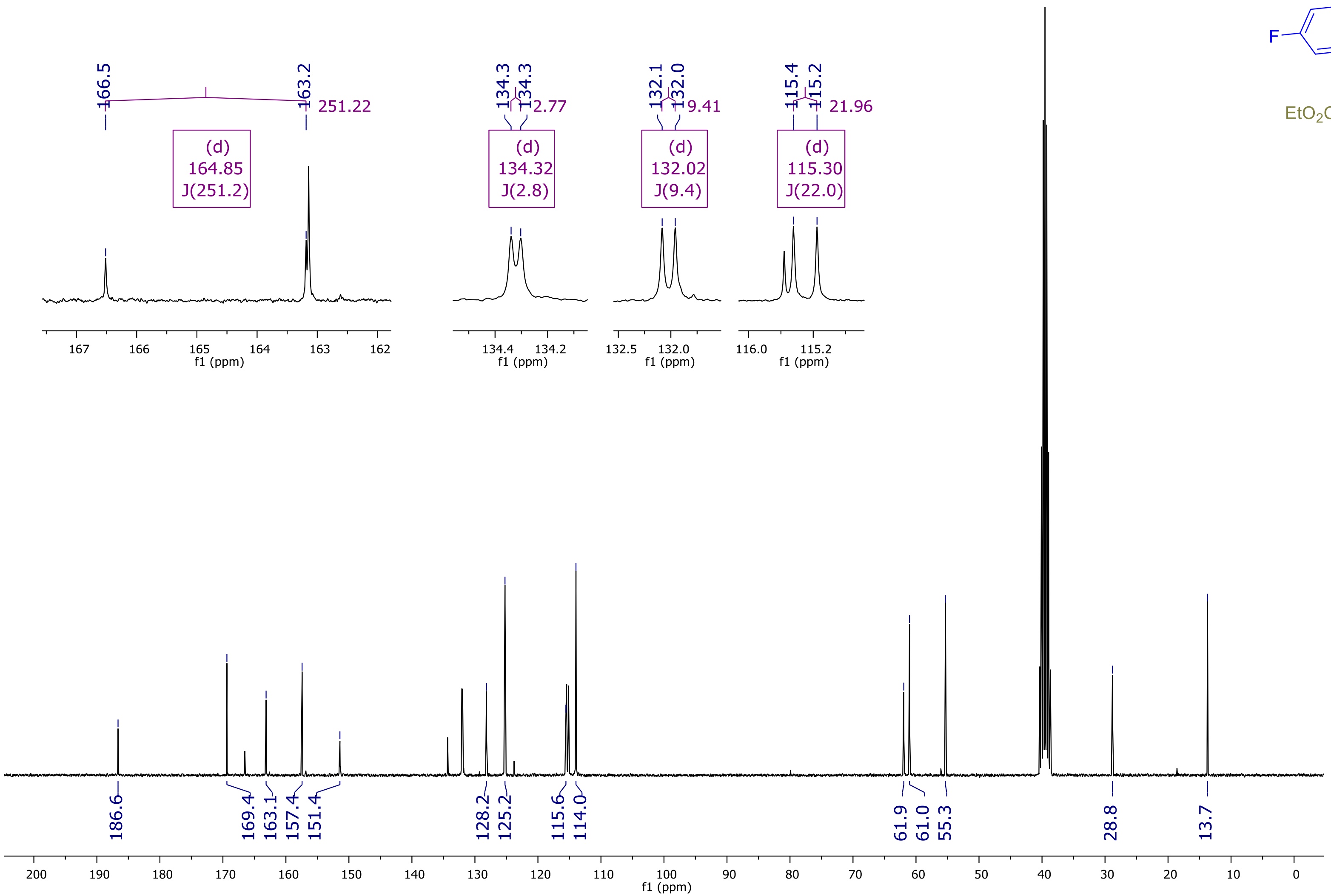

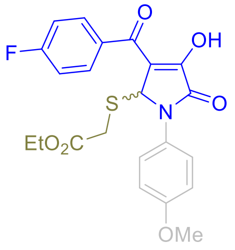

3h

Figure S21. $^{13}C\{^{1}H\}$ NMR Spectrum (75.46 MHz, DMSO-d_6) of compound **3h**

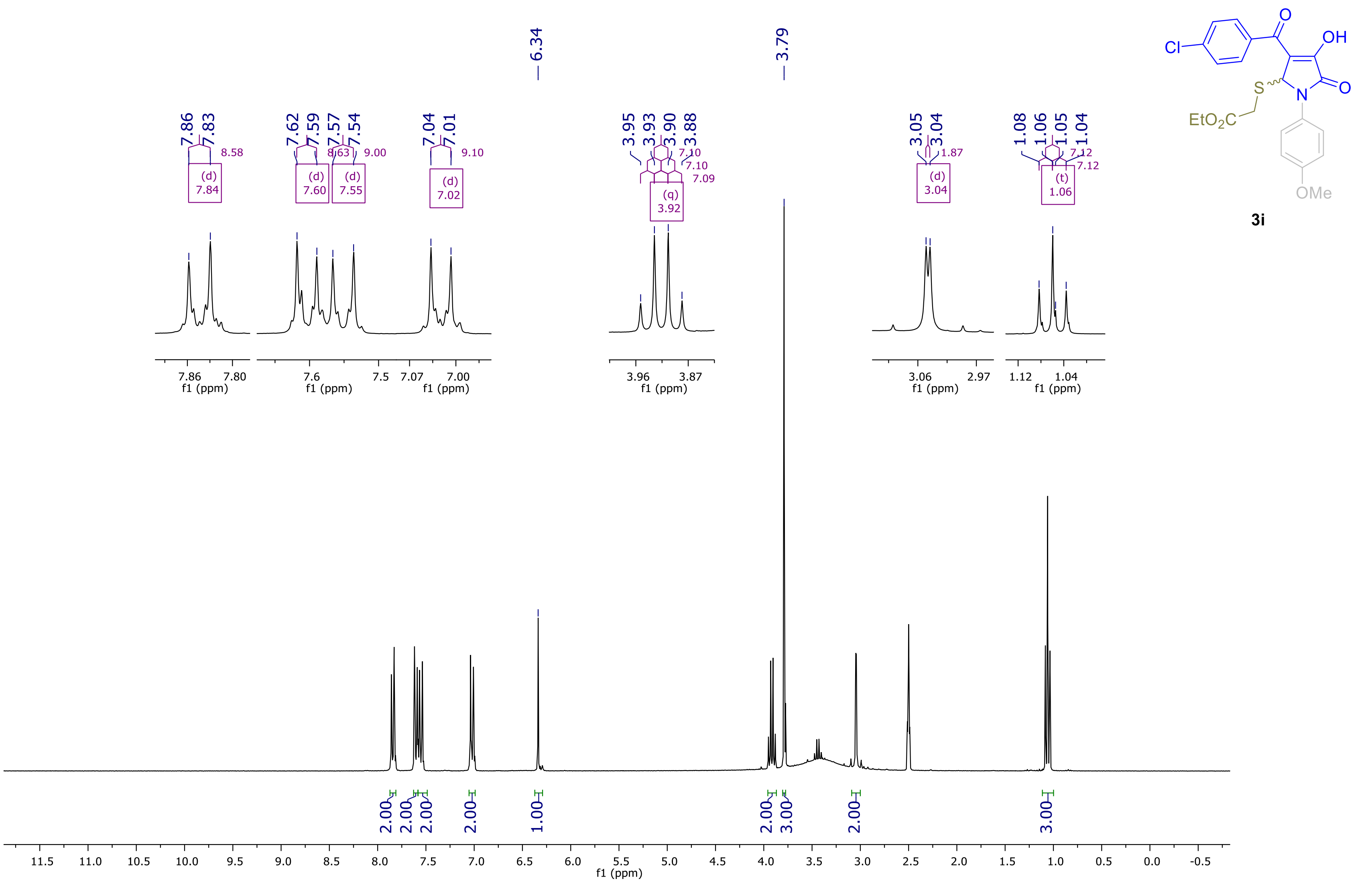

Figure S22. ^{1}H NMR Spectrum (300.06 MHz, DMSO-d_6) of compound **3i**

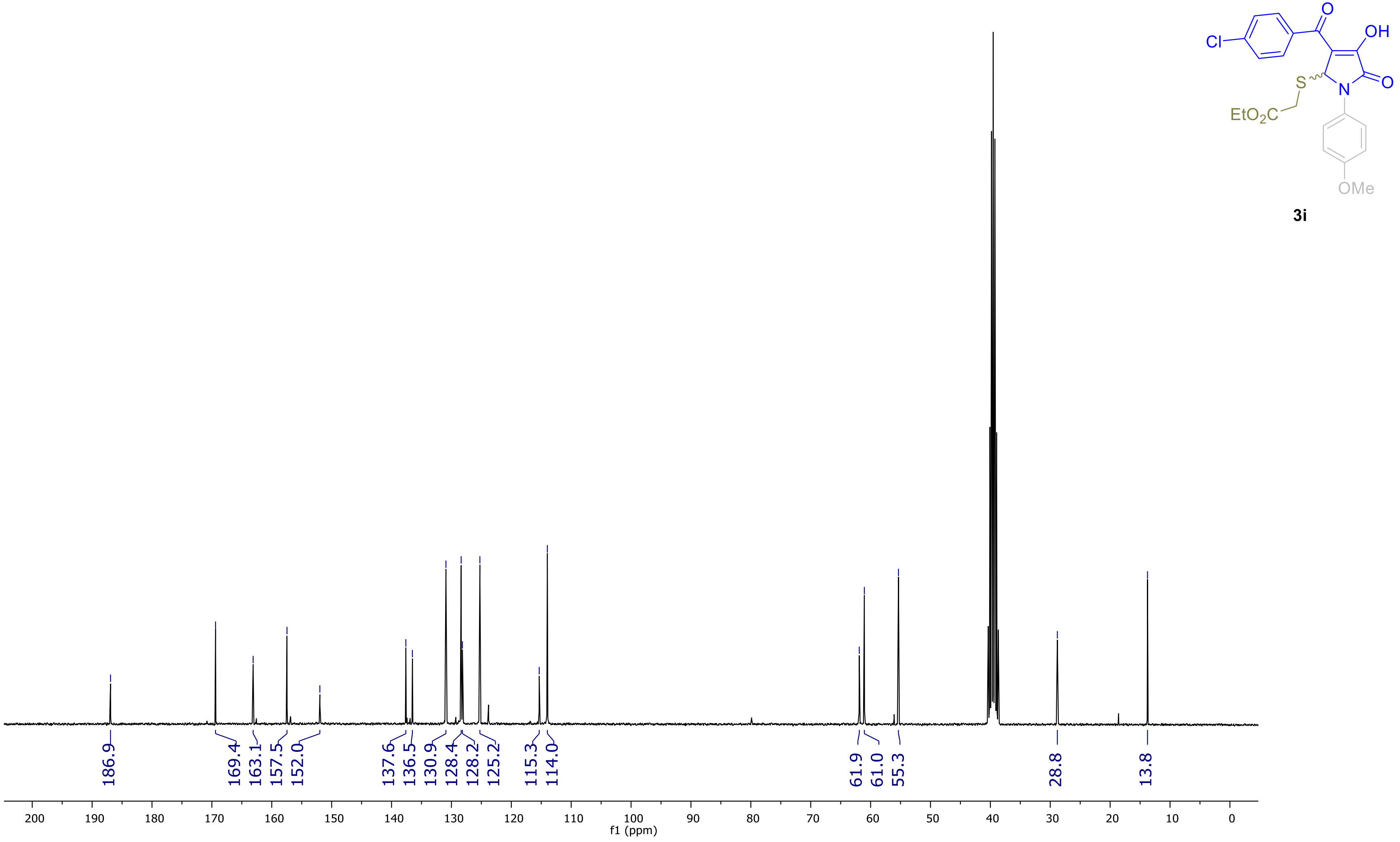

Figure S23. $^{13}C\{^{1}H\}$ NMR Spectrum (75.46 MHz, DMSO-d_6) of compound **3i**

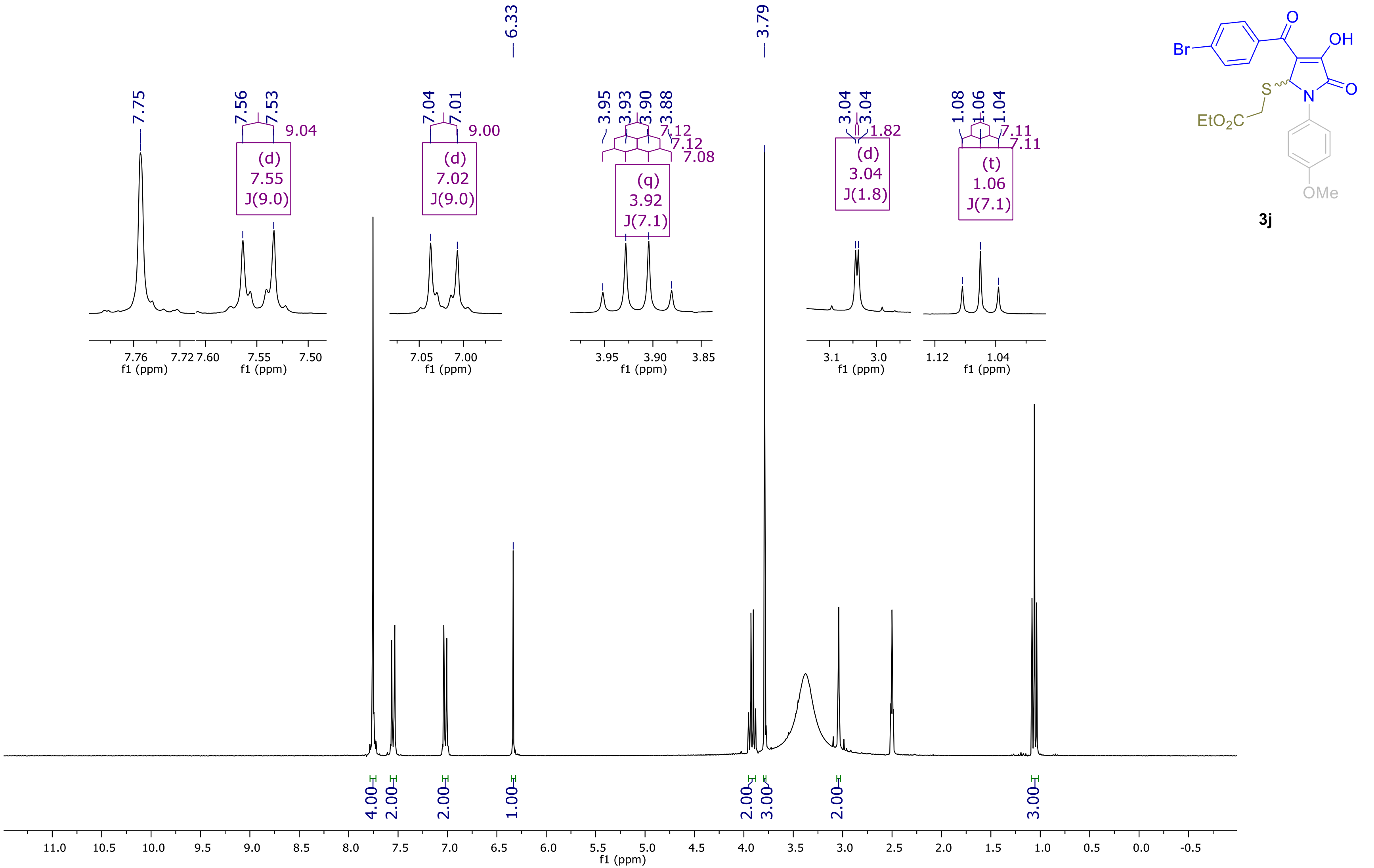

Figure S24. ^{1}H NMR Spectrum (300.06 MHz, DMSO-d_6) of compound **3j**

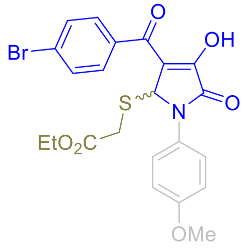

3j

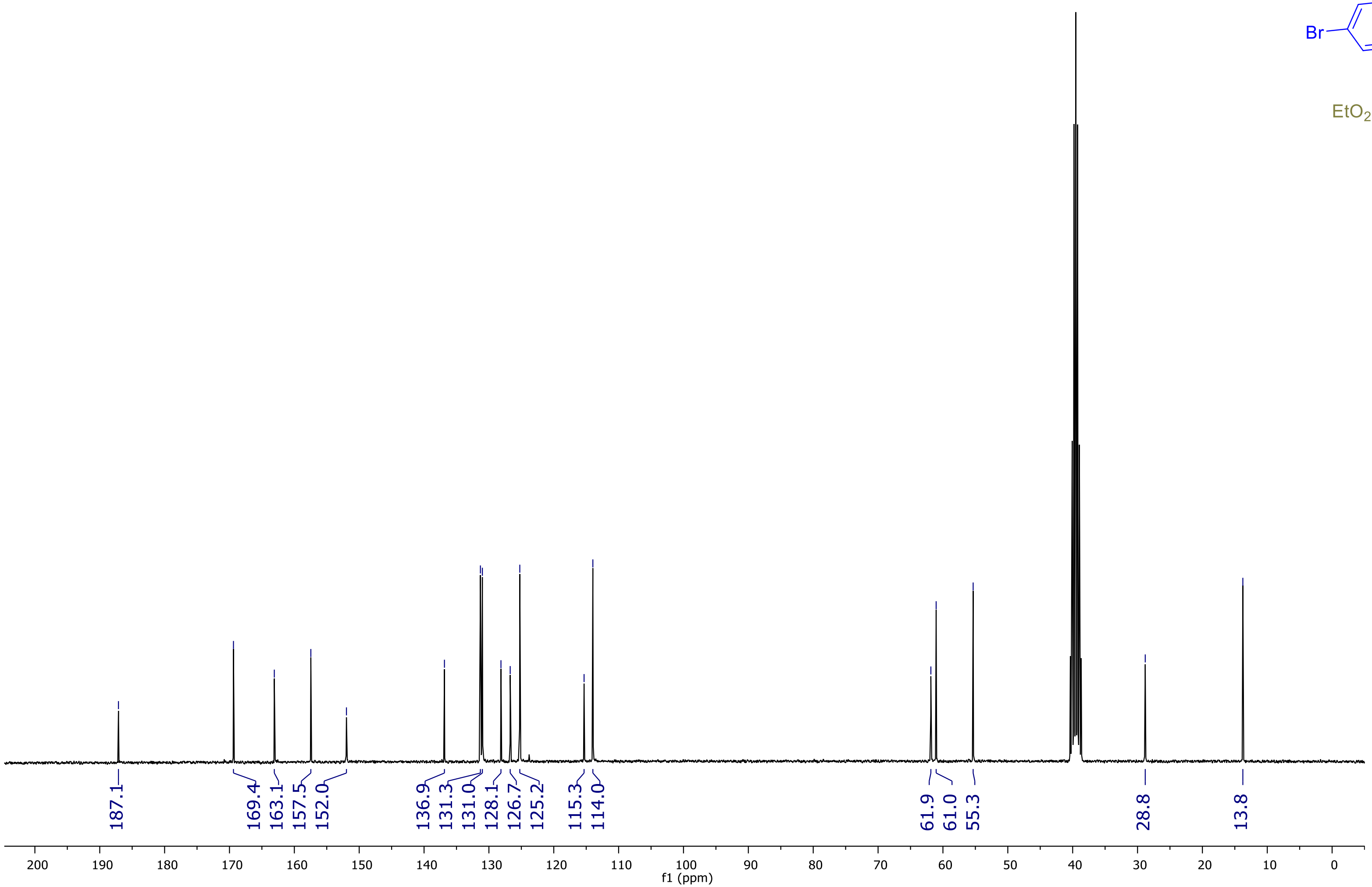

Figure S25. $^{13}C\{^{1}H\}$ NMR Spectrum (75.46 MHz, DMSO-d_6) of compound **3j**

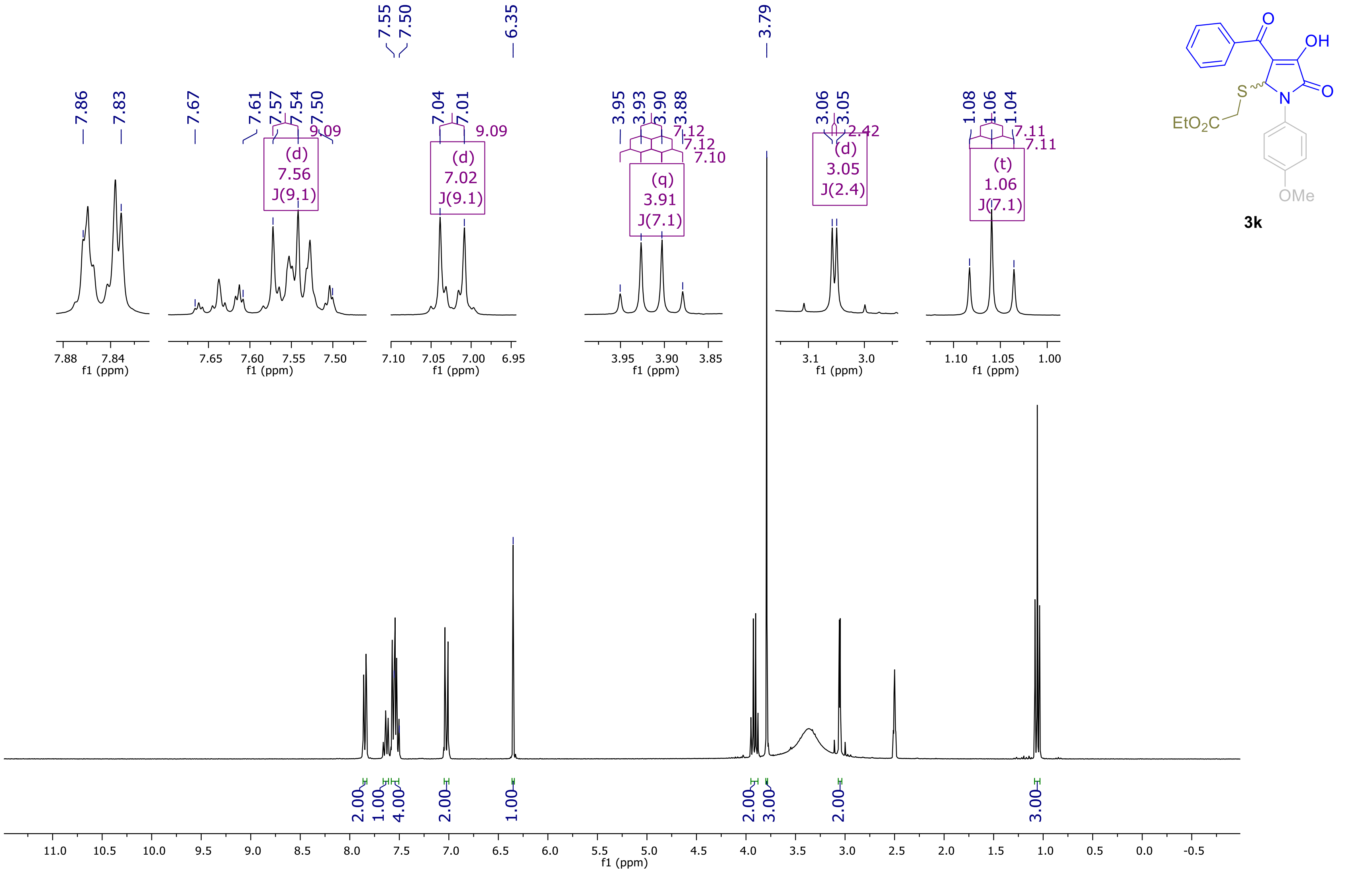

Figure S26. ^{1}H NMR Spectrum (300.06 MHz, DMSO-d_6) of compound **3k**

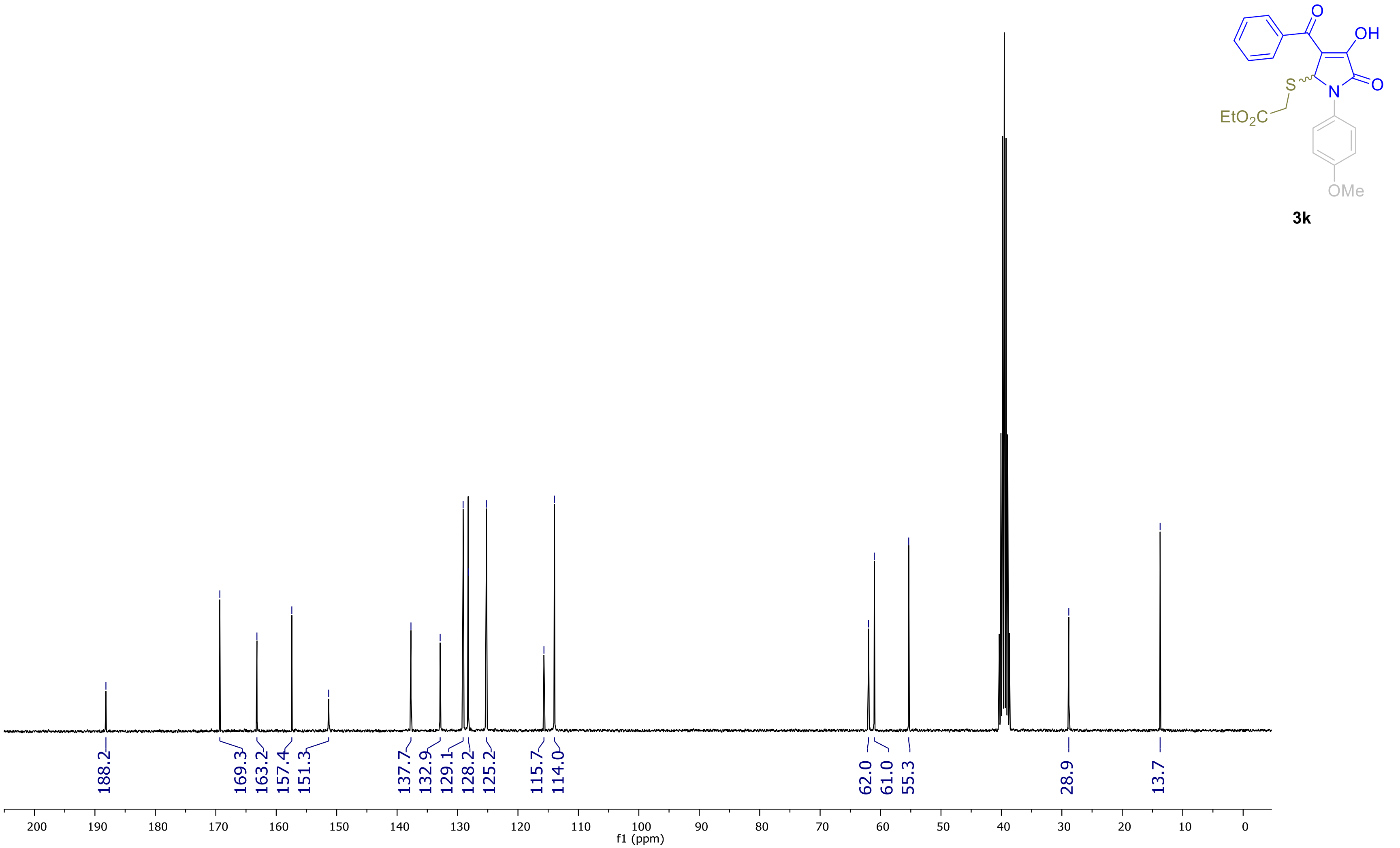

Figure S27. $^{13}C\{^{1}H\}$ NMR Spectrum (75.46 MHz, DMSO-d_6) of compound **3k**

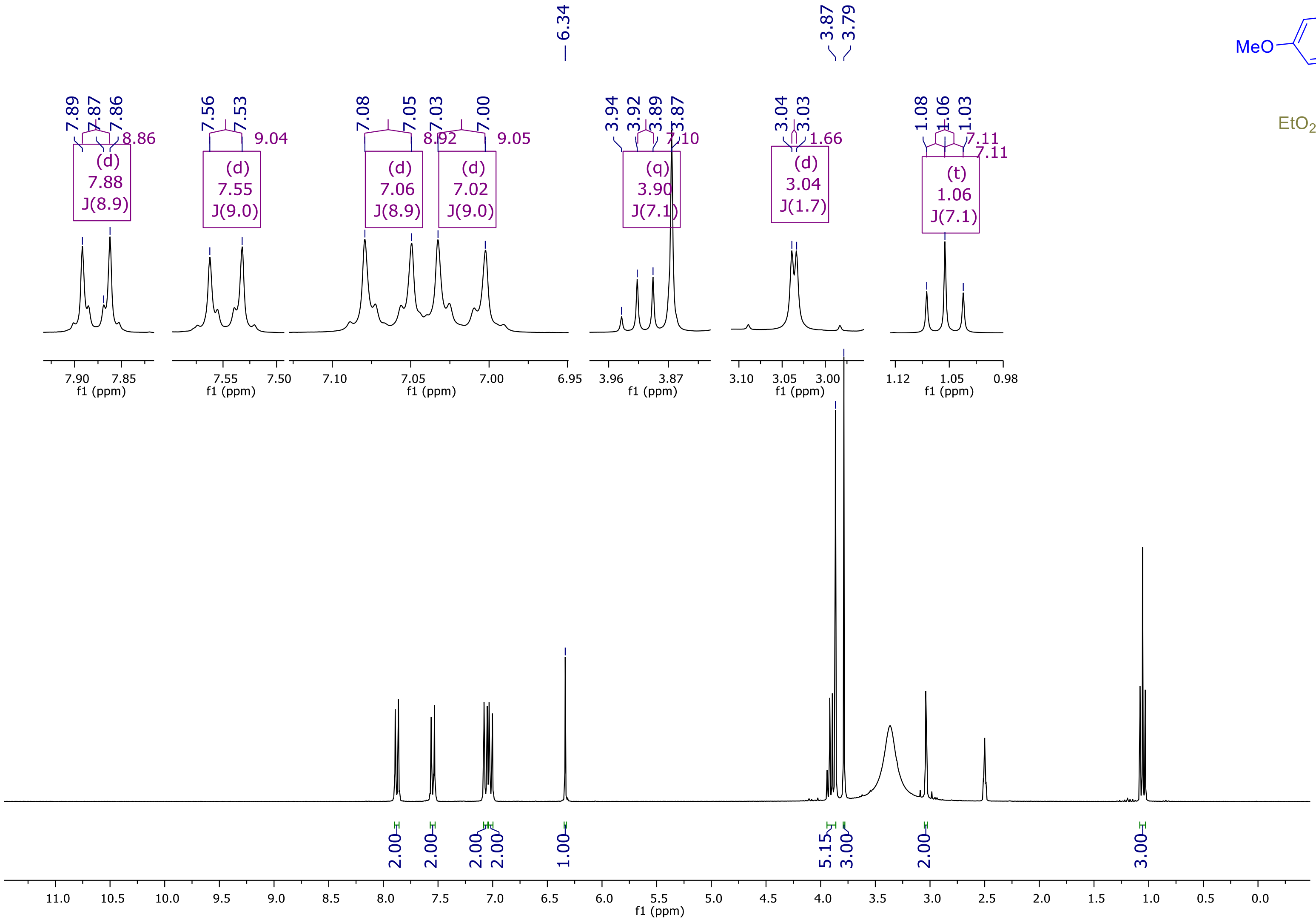

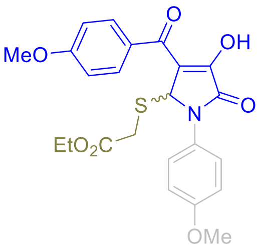

3l

Figure S28. ^1H NMR Spectrum (300.06 MHz, DMSO-d_6) of compound **3l**

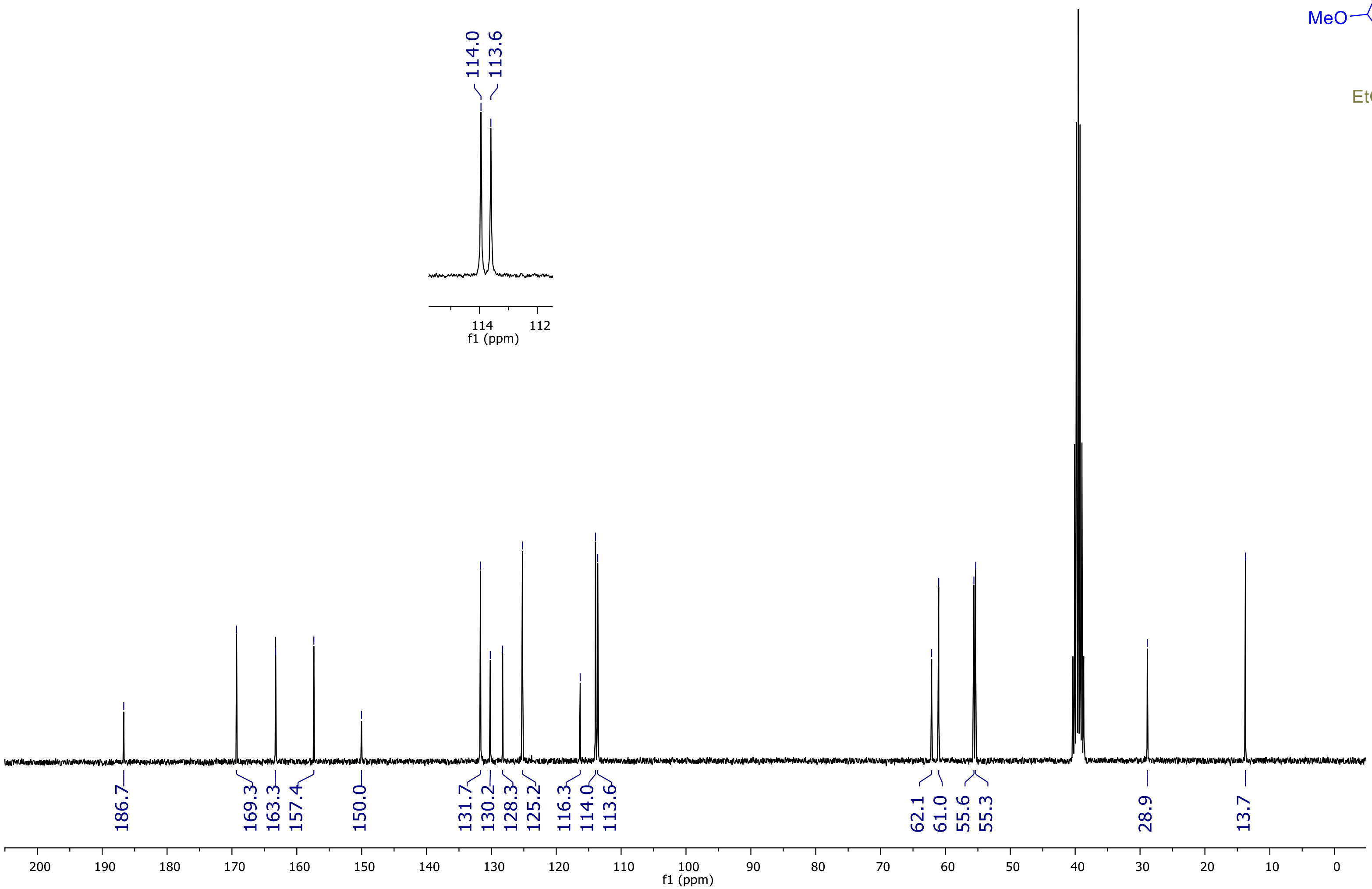

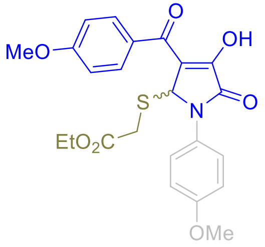

3l

Figure S29. $^{13}C\{^{1}H\}$ NMR Spectrum (75.46 MHz, DMSO-d_6) of compound **3l**

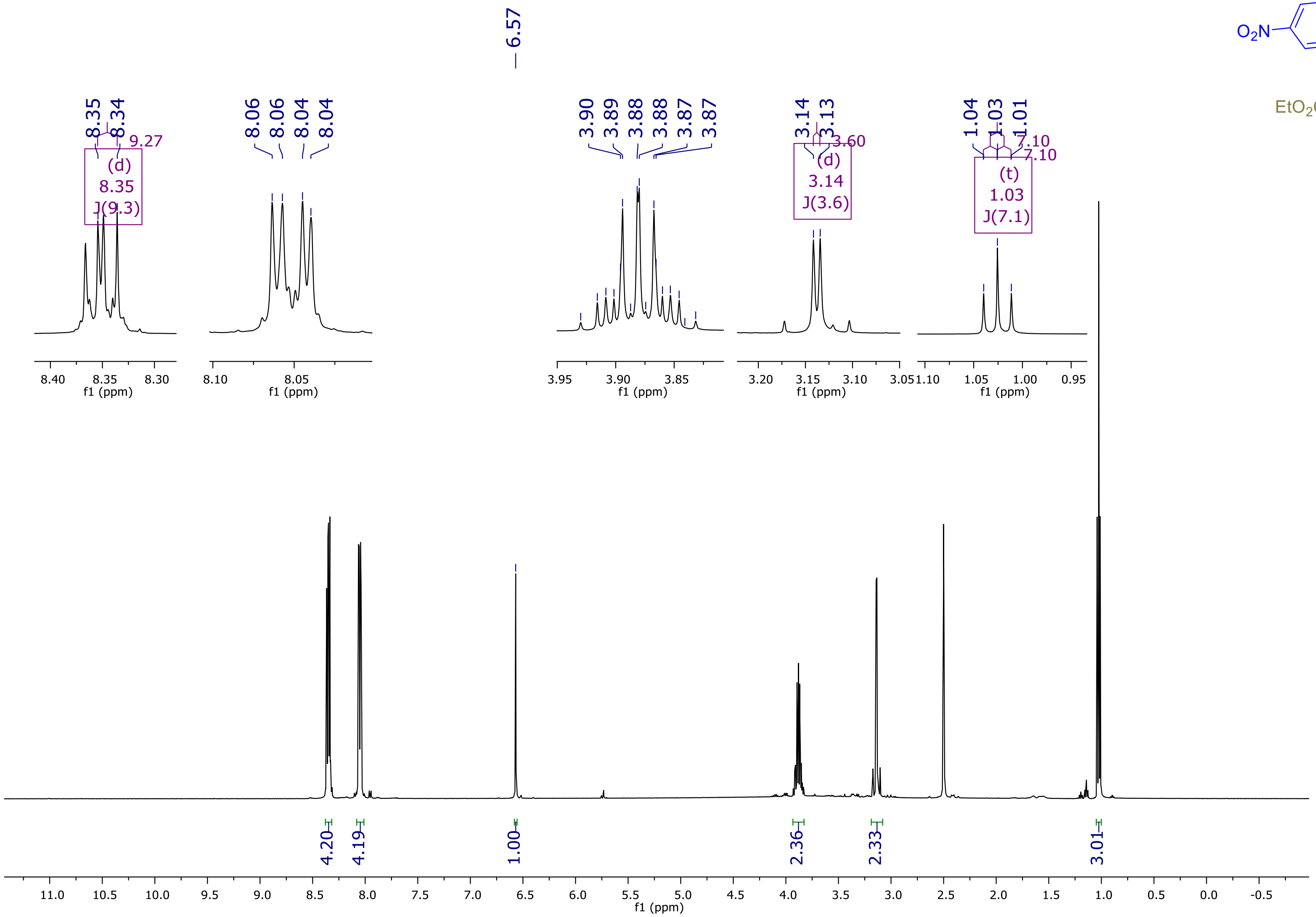

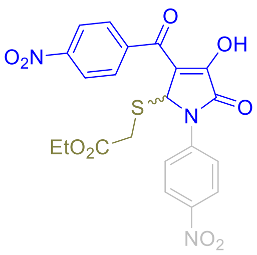

3m

Figure S30. ^{1}H NMR Spectrum (500.13 MHz, DMSO-d$_6$) of compound **3m**

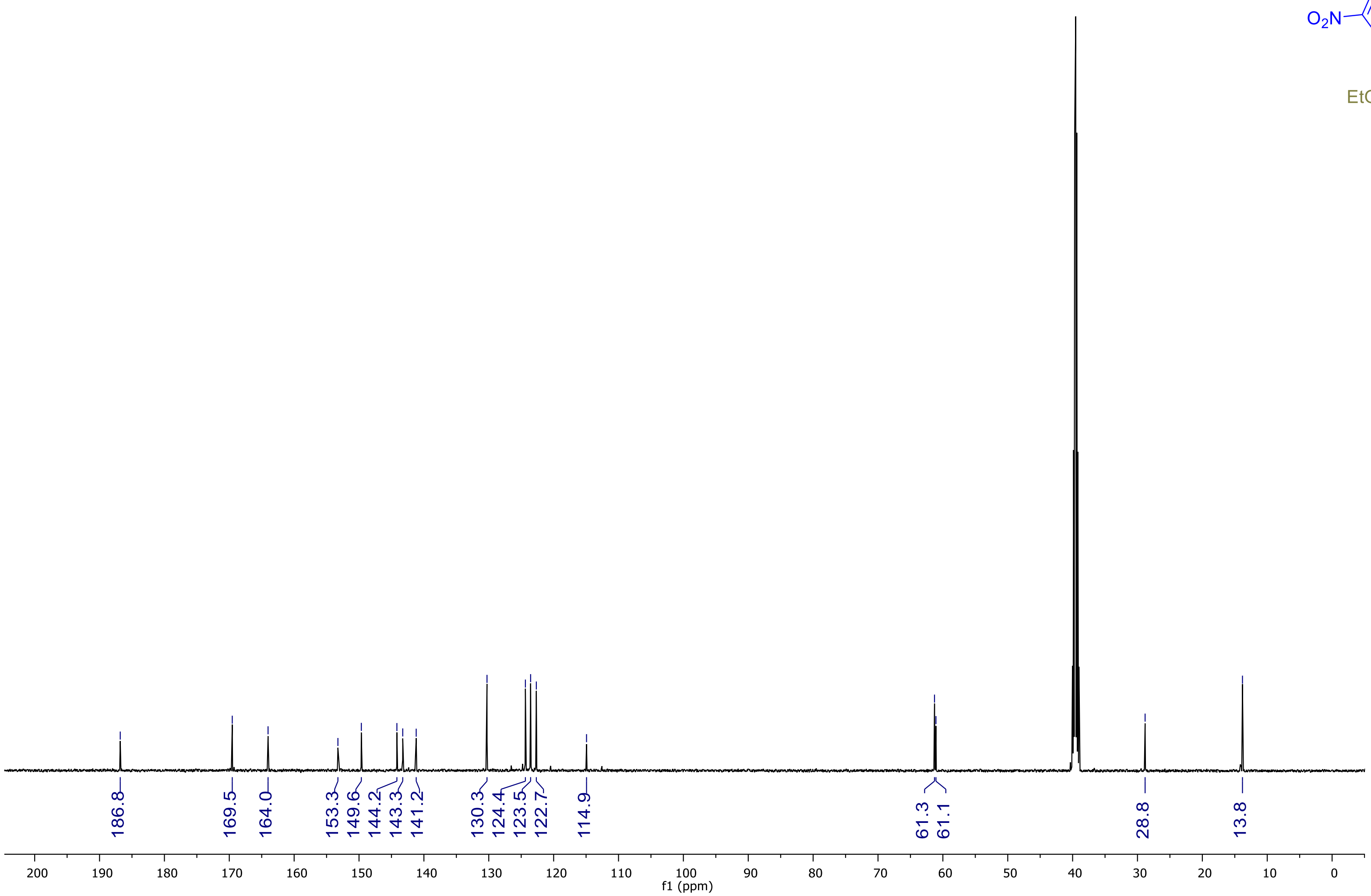

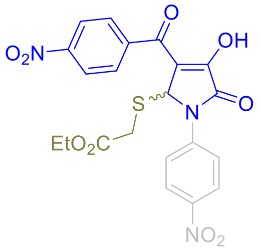

3m

Figure S31. $^{13}C\{^{1}H\}$ NMR Spectrum (125.77 MHz, DMSO-d_6) of compound **3m**

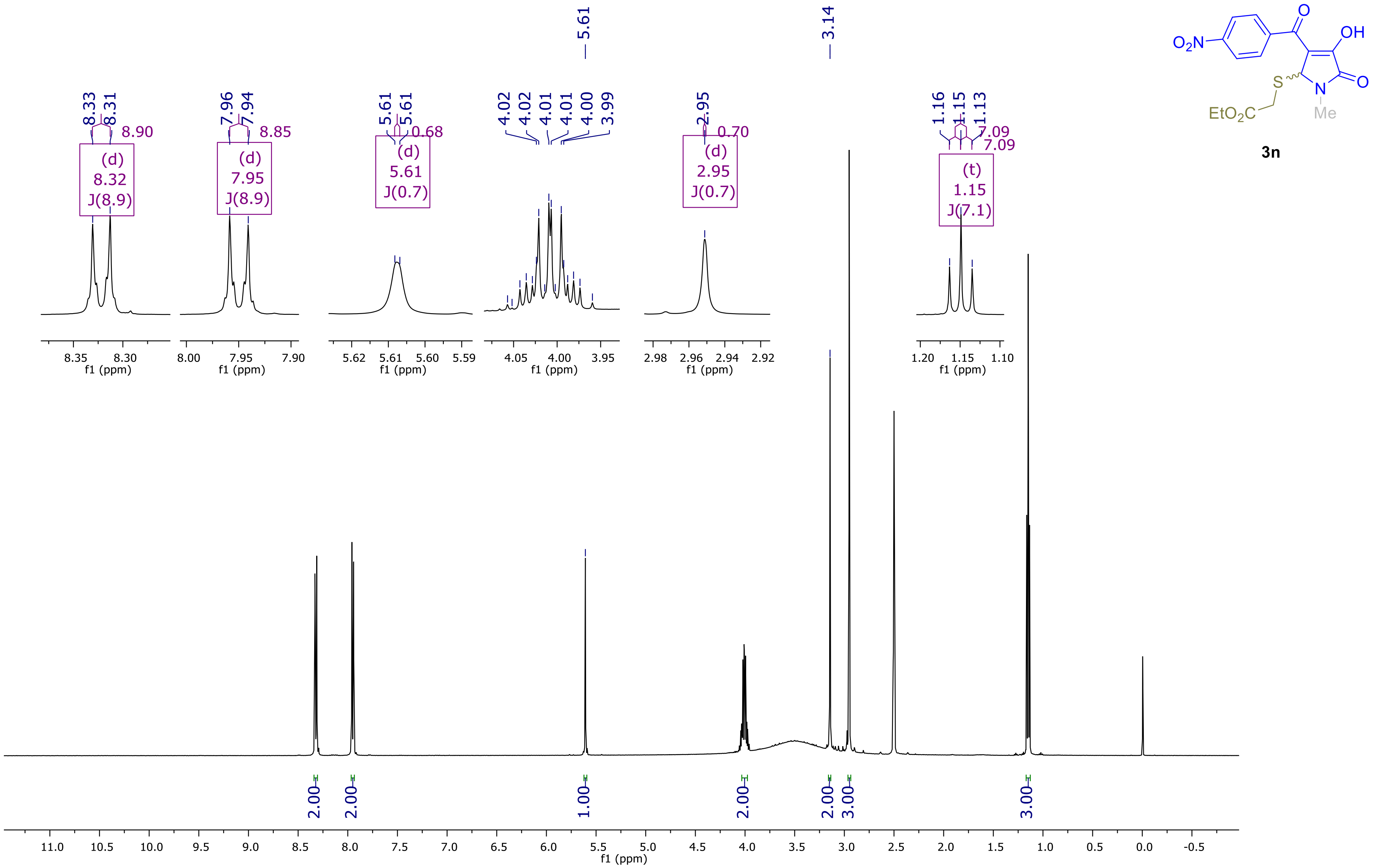

Figure S32. ^{1}H NMR Spectrum (500.13 MHz, DMSO-d_6) of compound **3n**

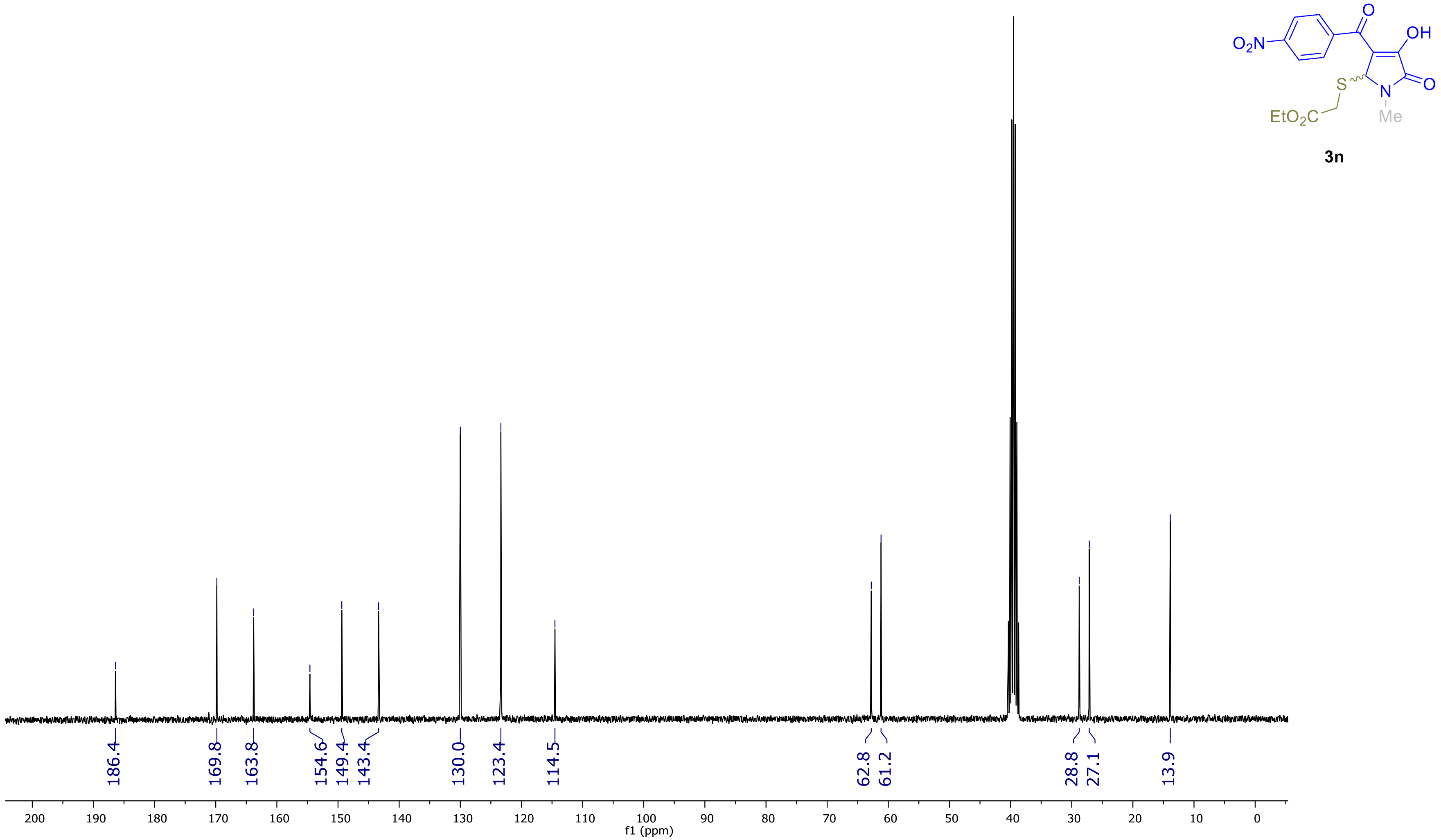

Figure S33. $^{13}C\{^{1}H\}$ NMR Spectrum (75.46 MHz, DMSO-d_6) of compound **3n**

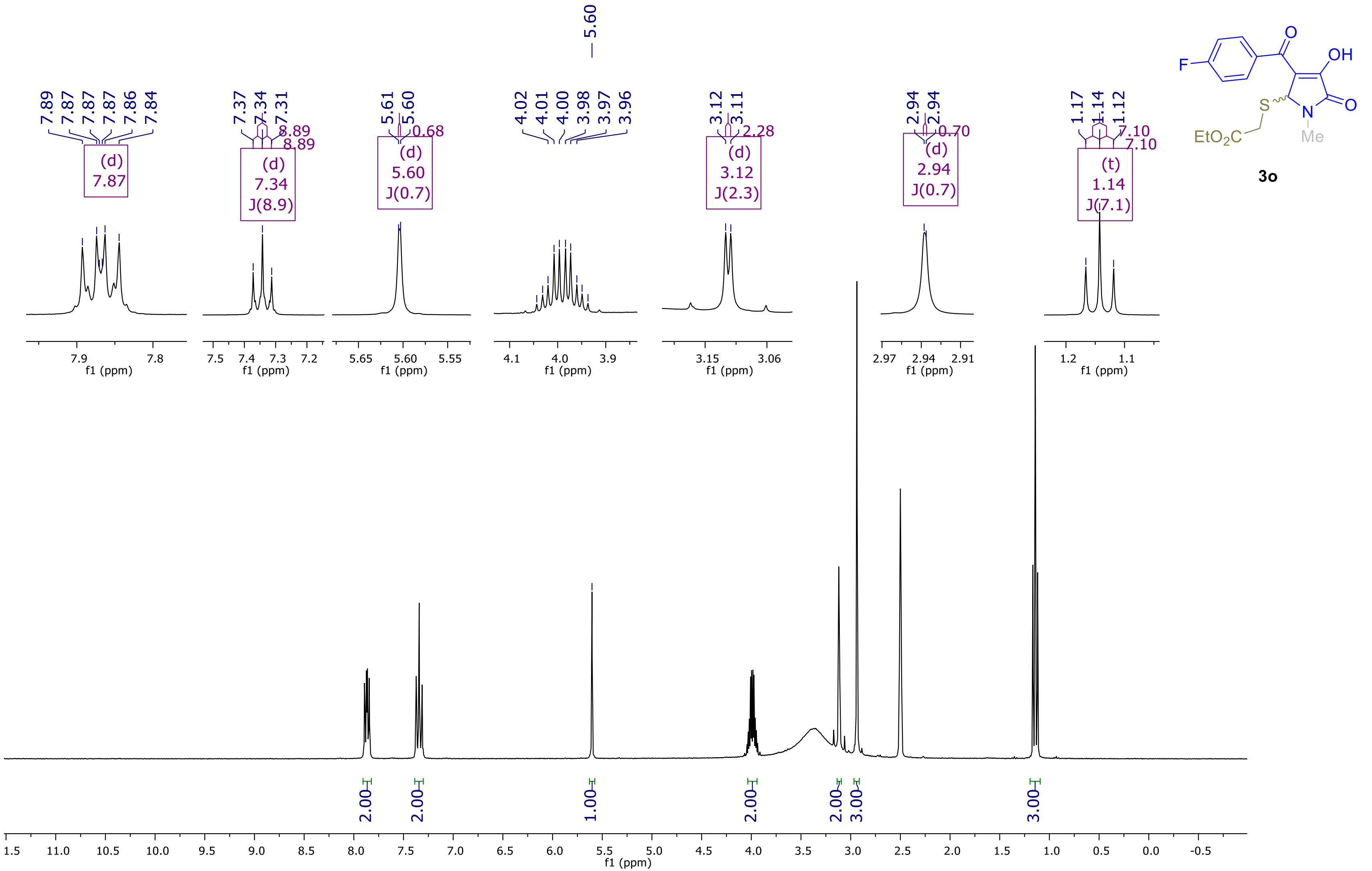

Figure S34. ^{1}H NMR Spectrum (300.06 MHz, DMSO-d_6) of compound **3o**

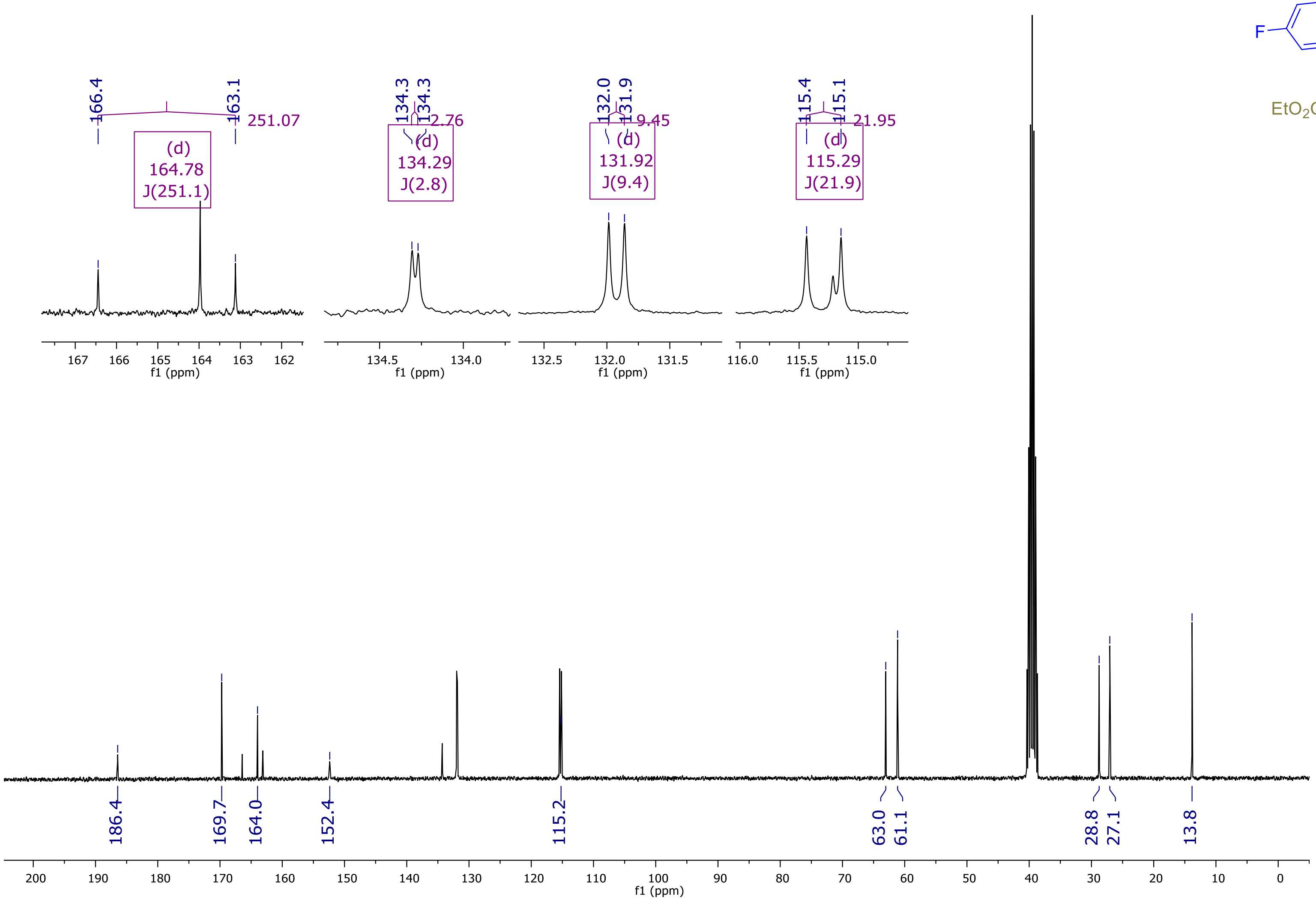

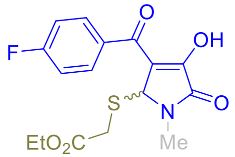

3o

Figure S35. $^{13}C\{^{1}H\}$ NMR Spectrum (75.46 MHz, DMSO-d_6) of compound **3o**

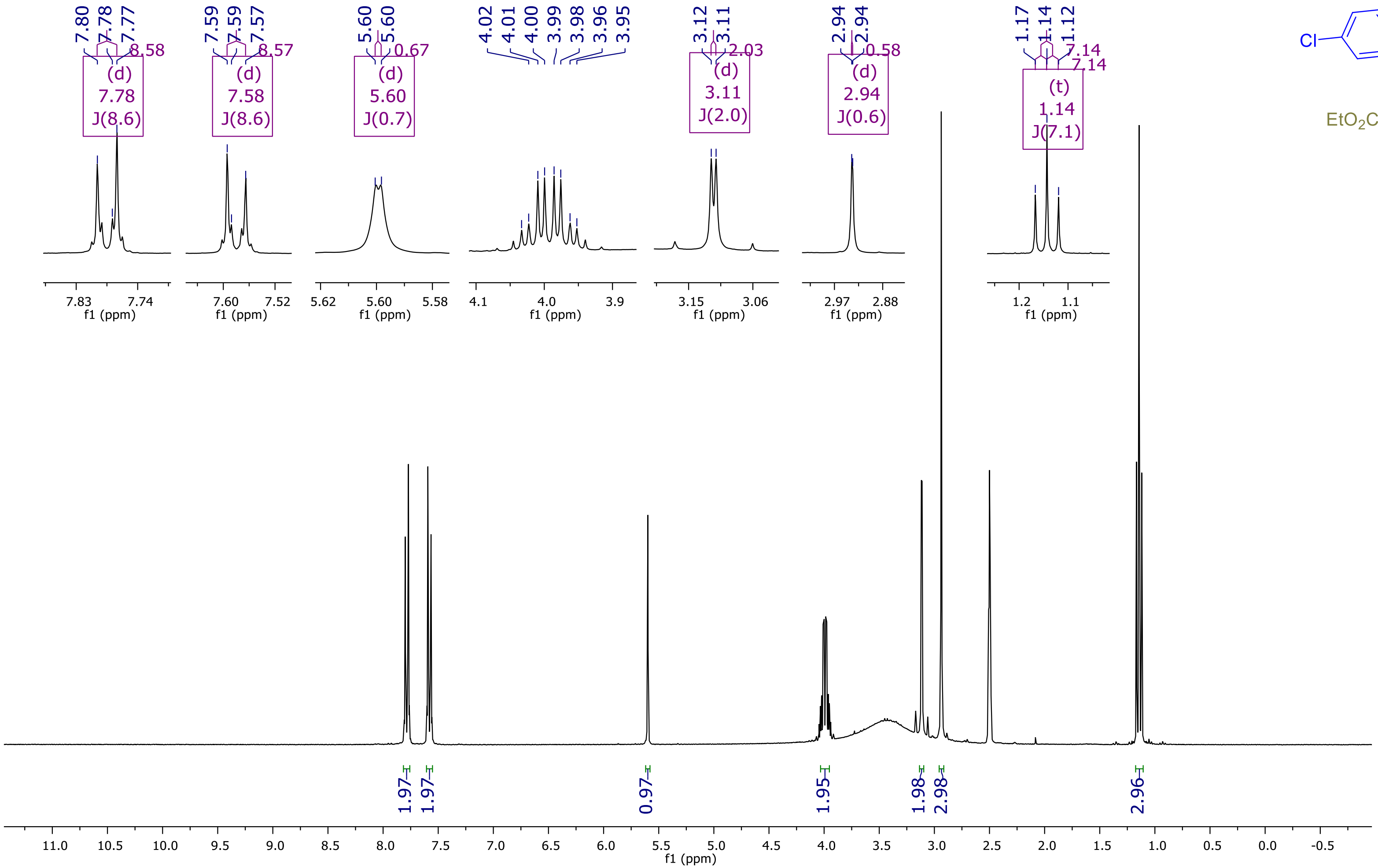

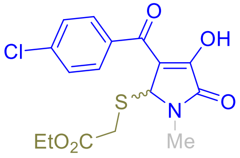

3p

Figure S36. ^{1}H NMR Spectrum (300.06 MHz, DMSO-d_6) of compound **3p**

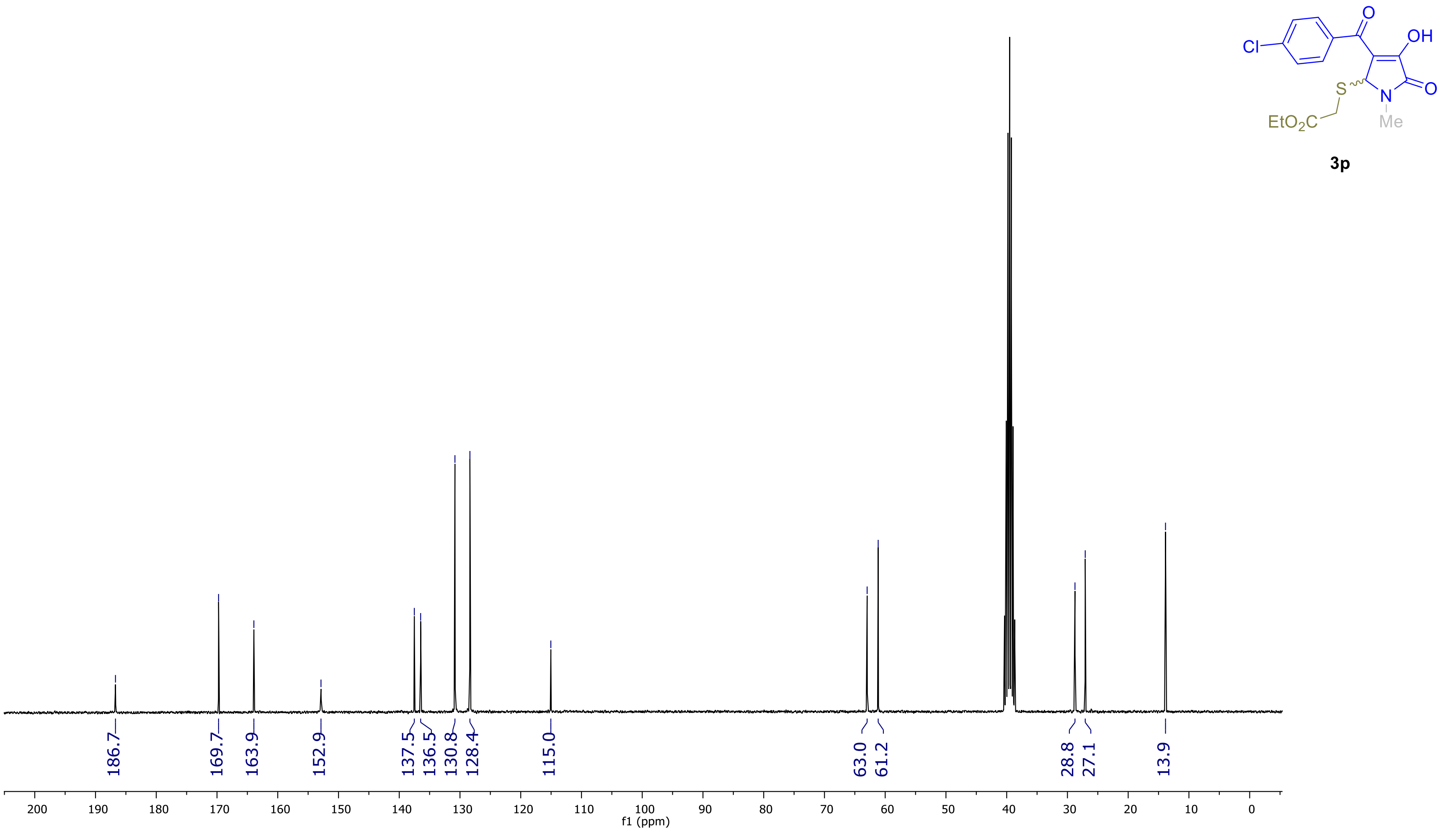

Figure S37. $^{13}C\{^{1}H\}$ NMR Spectrum (75.46 MHz, DMSO-d_6) of compound **3p**

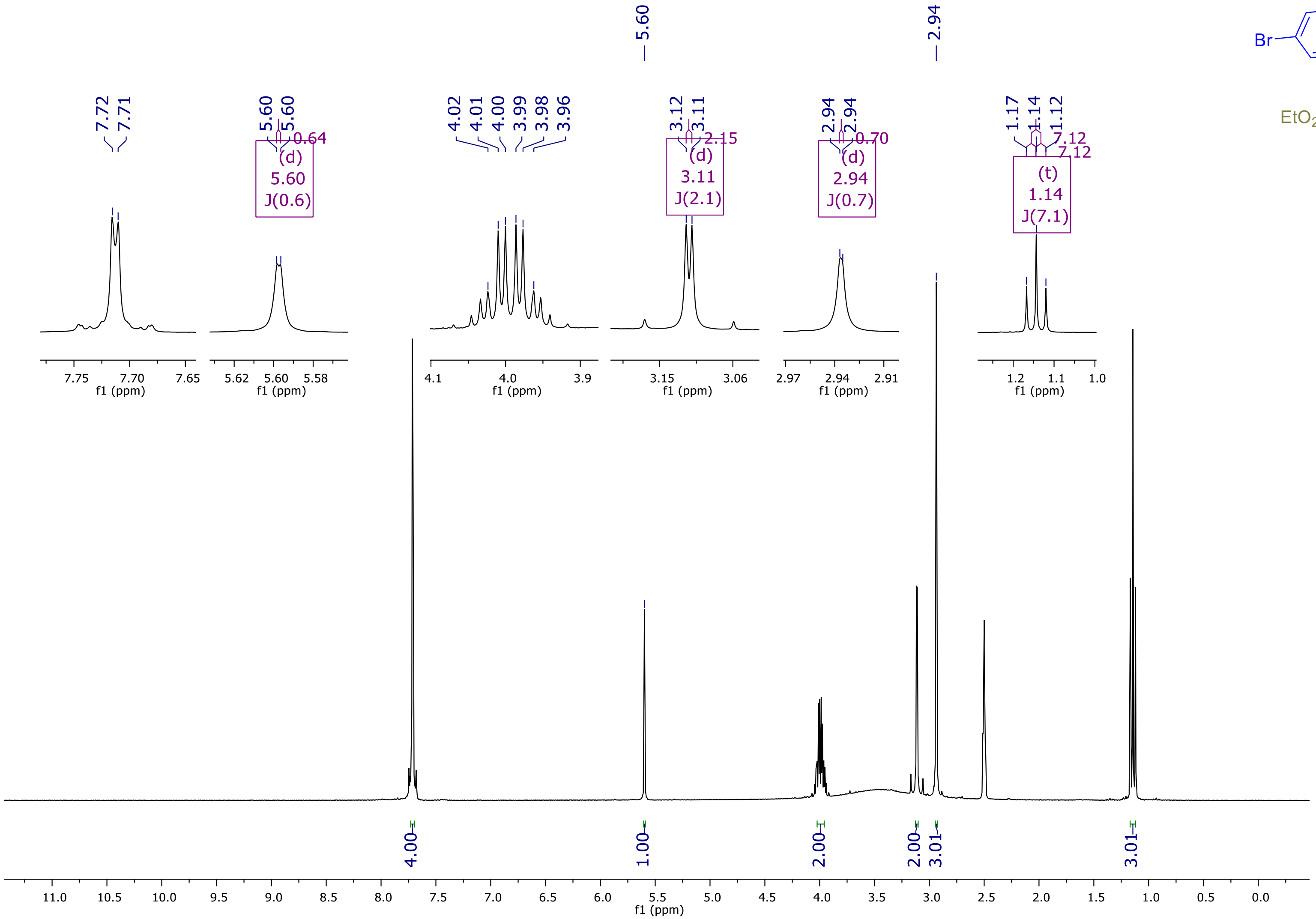

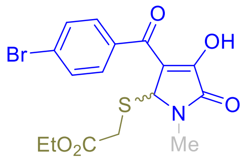

3q

Figure S38. ^{1}H NMR Spectrum (300.06 MHz, DMSO-d_6) of compound **3q**

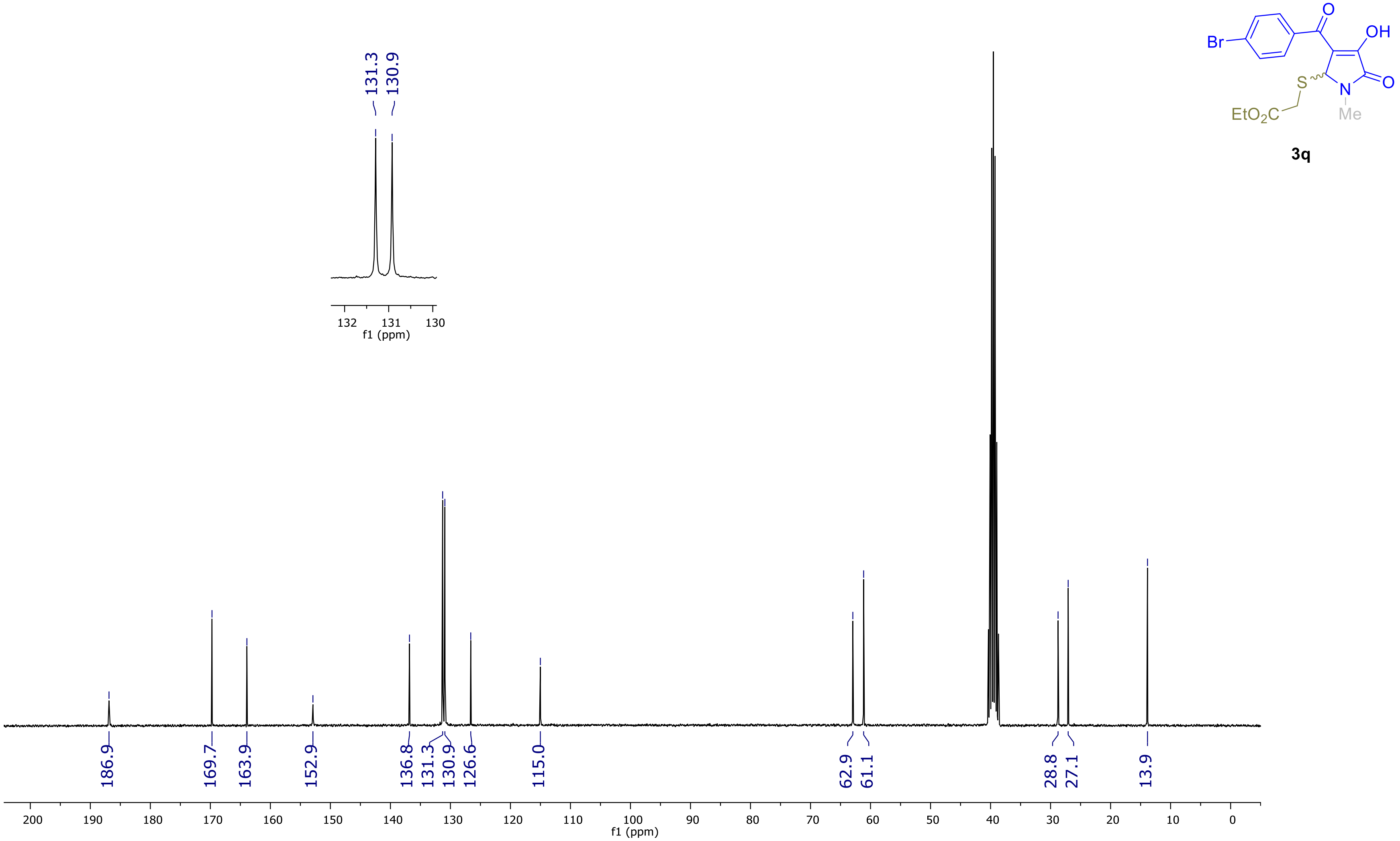

3q

Figure S39. $^{13}C\{^{1}H\}$ NMR Spectrum (75.46 MHz, DMSO-d_6) of compound **3q**

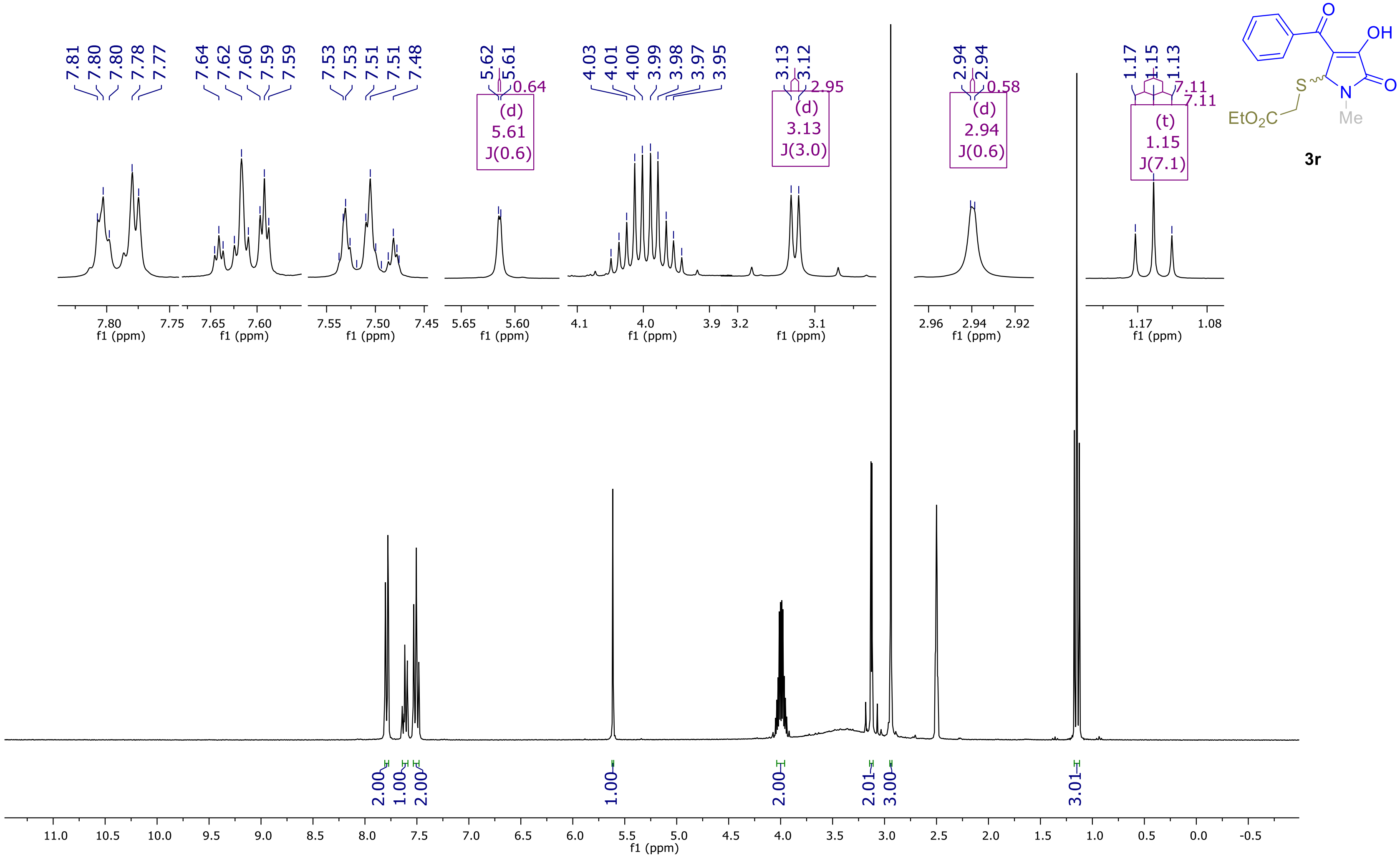

Figure S40. ^{1}H NMR Spectrum (300.06 MHz, DMSO-d_6) of compound **3r**

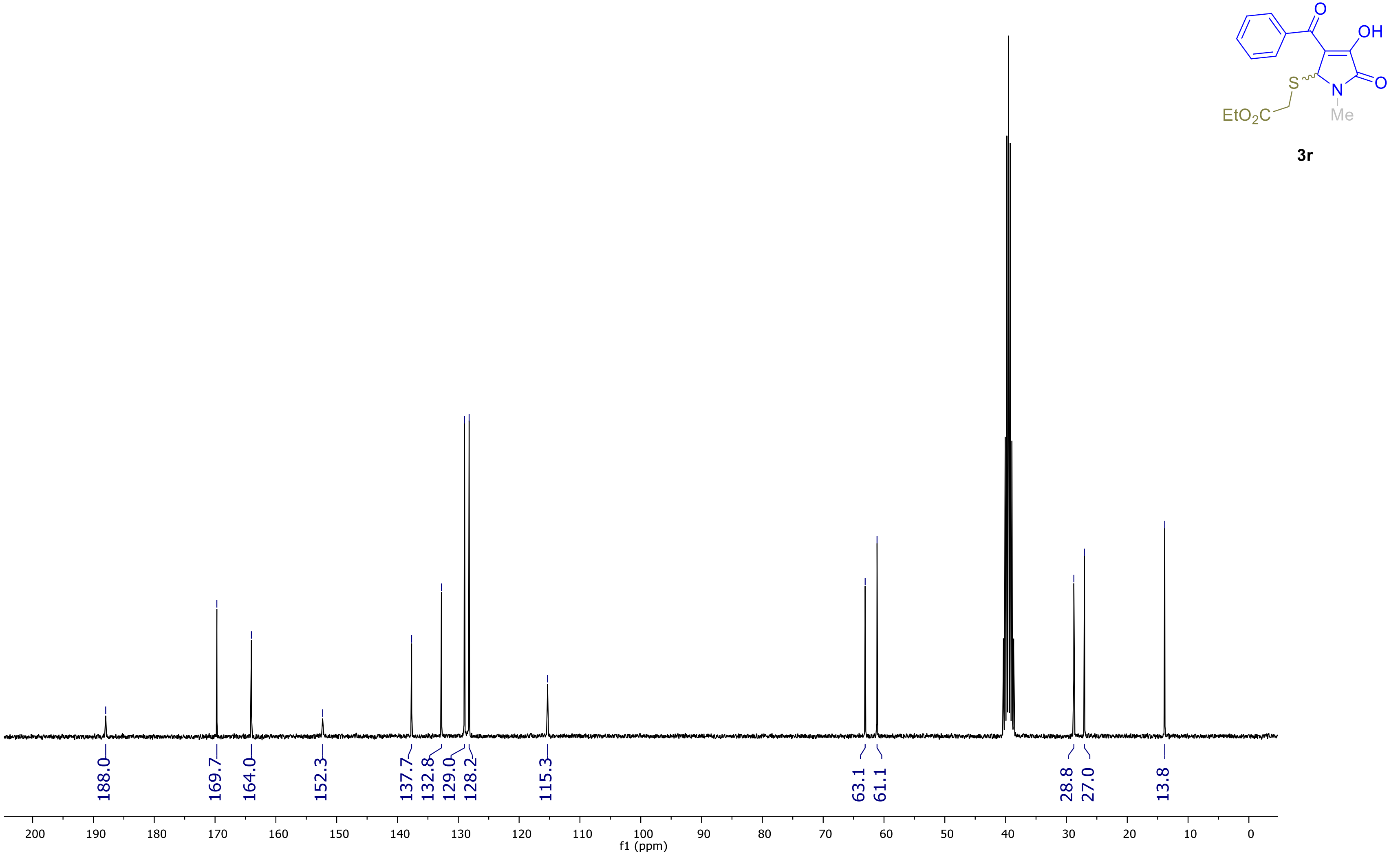

Figure S41. $^{13}C\{^{1}H\}$ NMR Spectrum (75.46 MHz, DMSO-d_6) of compound **3r**

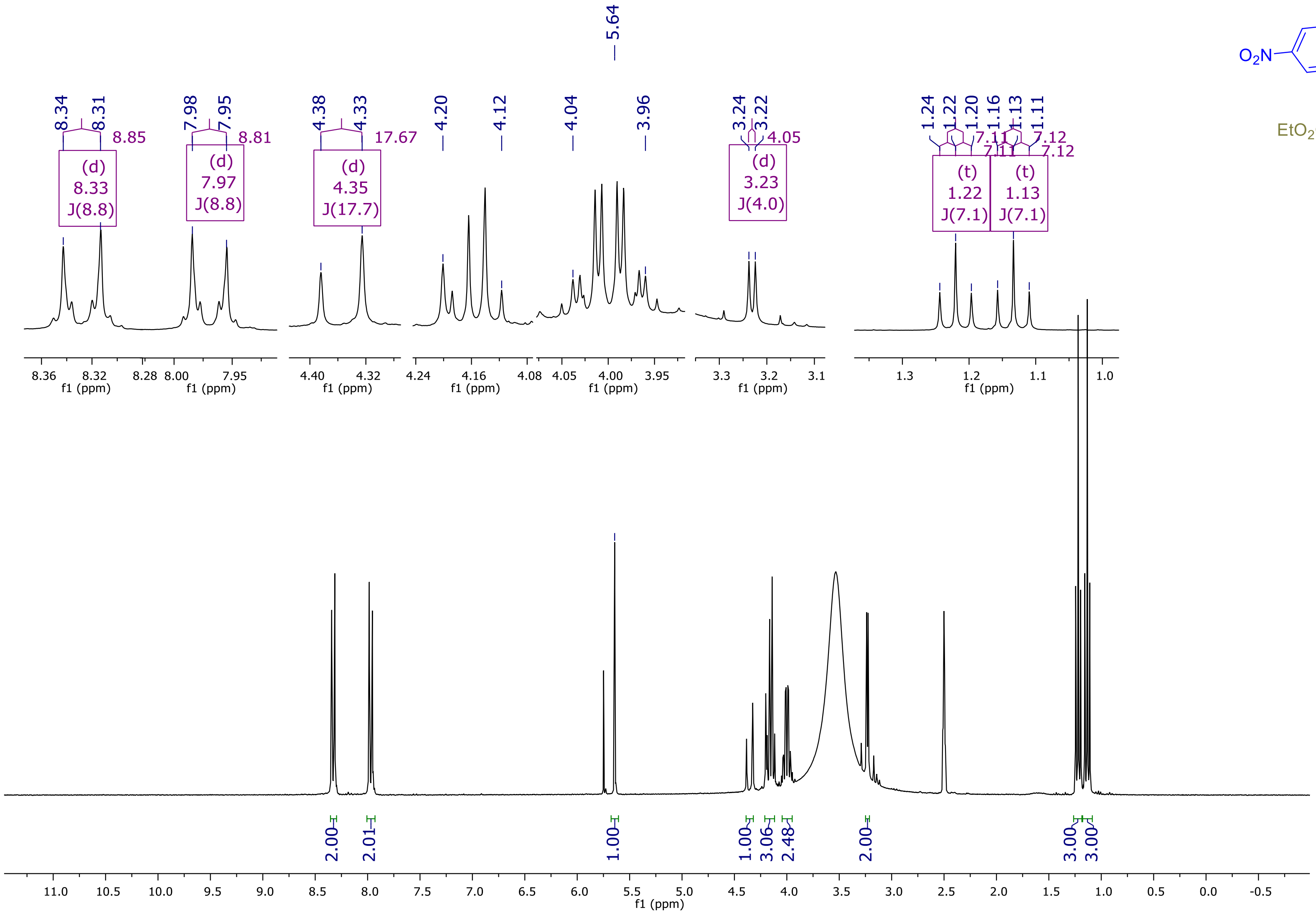

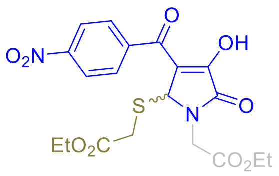

3s

Figure S42. ^{1}H NMR Spectrum (300.06 MHz, DMSO-d_6) of compound **3s**

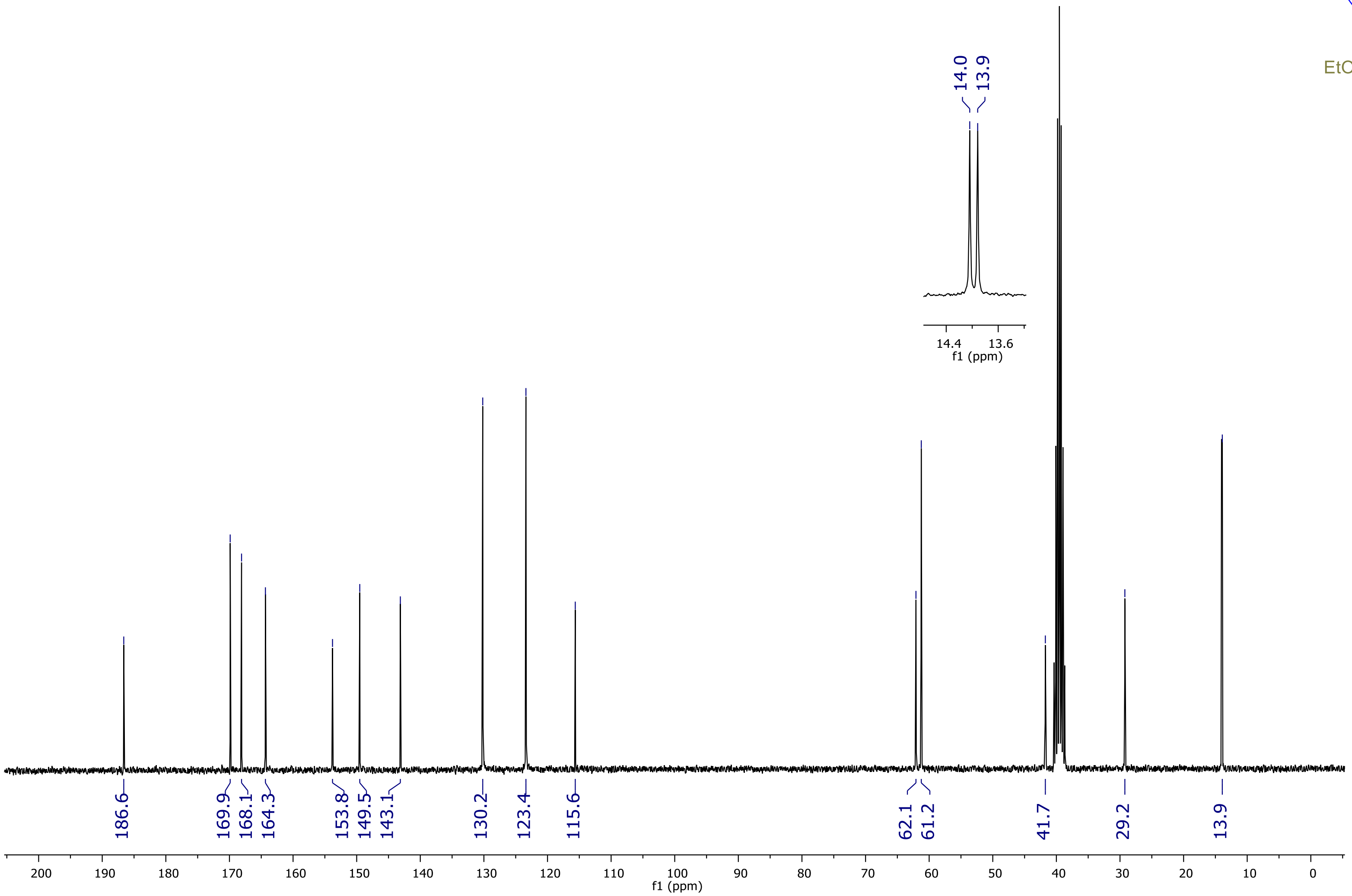

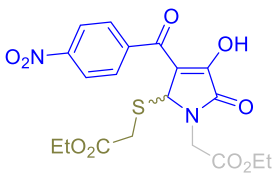

3s

Figure S43. $^{13}C\{^1H\}$ NMR Spectrum (75.46 MHz, DMSO-d_6) of compound **3s**

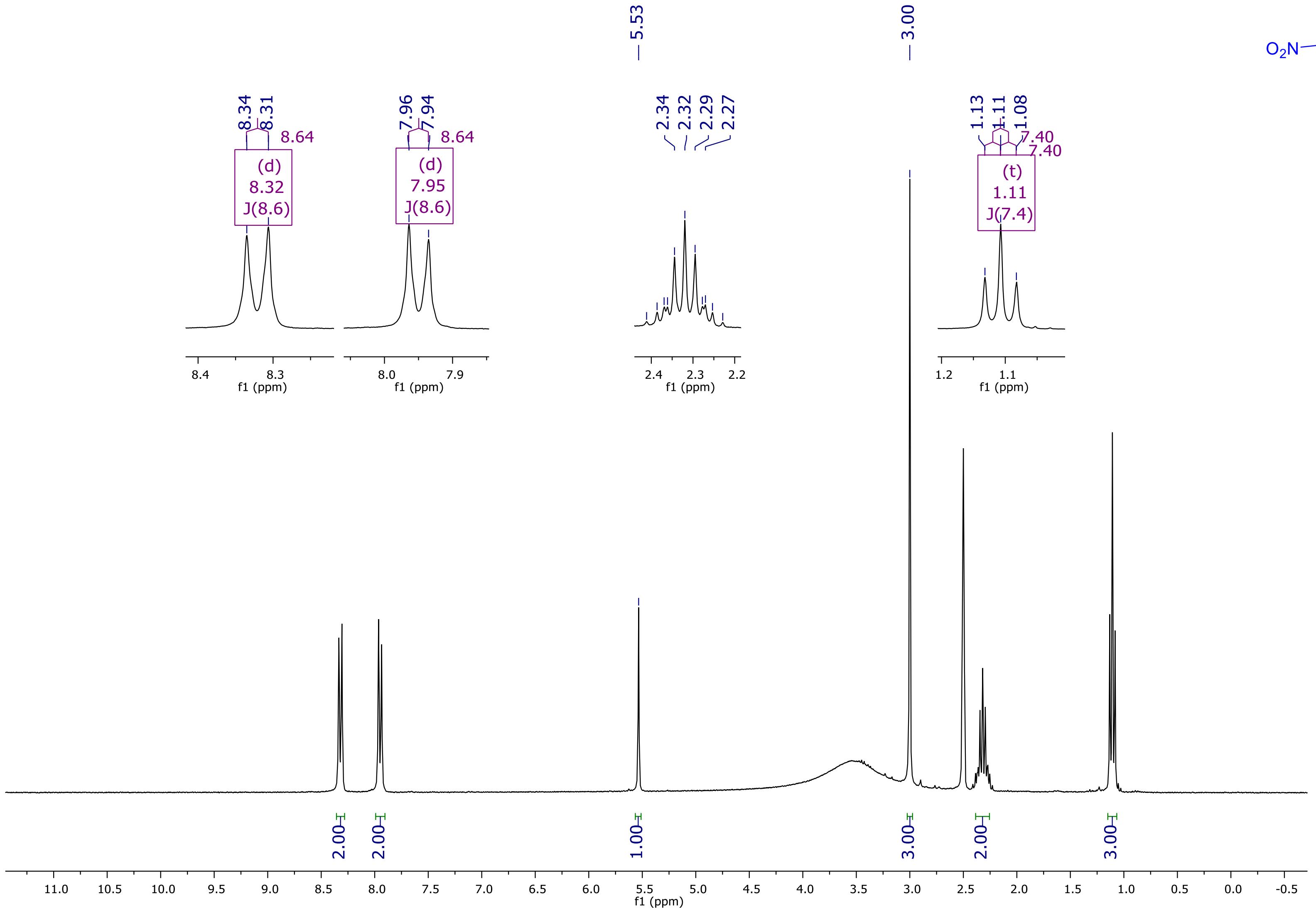

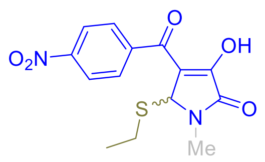

3t

Figure S44. ^{1}H NMR Spectrum (300.06 MHz, DMSO-d_6) of compound **3t**

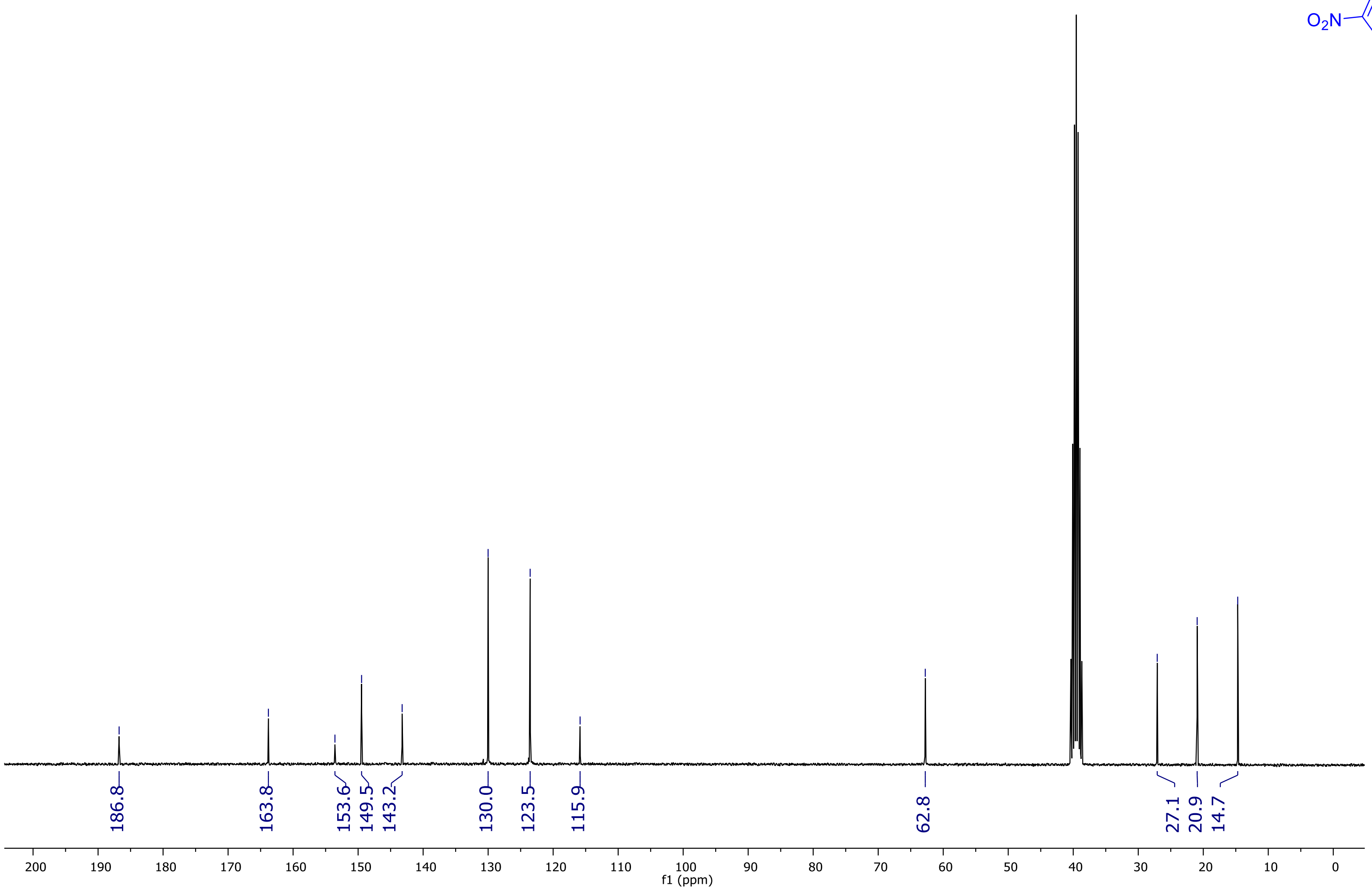

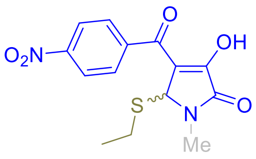

3t

Figure S45. $^{13}C\{^{1}H\}$ NMR Spectrum (75.46 MHz, DMSO-d_6) of compound **3t**

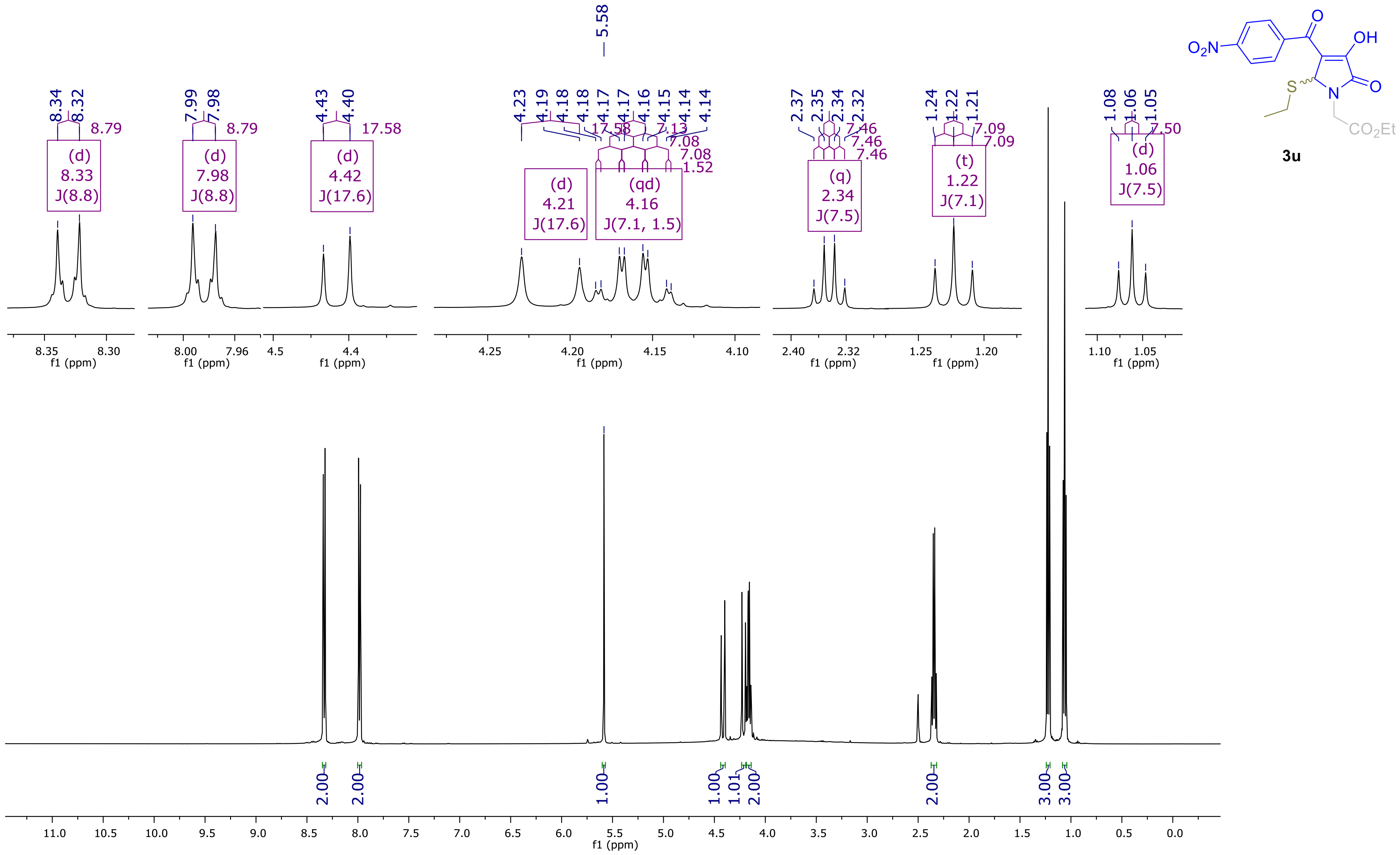

Figure S46. ^{1}H NMR Spectrum (500.13 MHz, DMSO-d_6) of compound **3u**

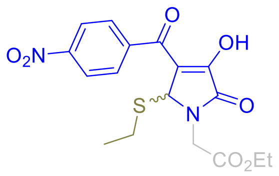

3u

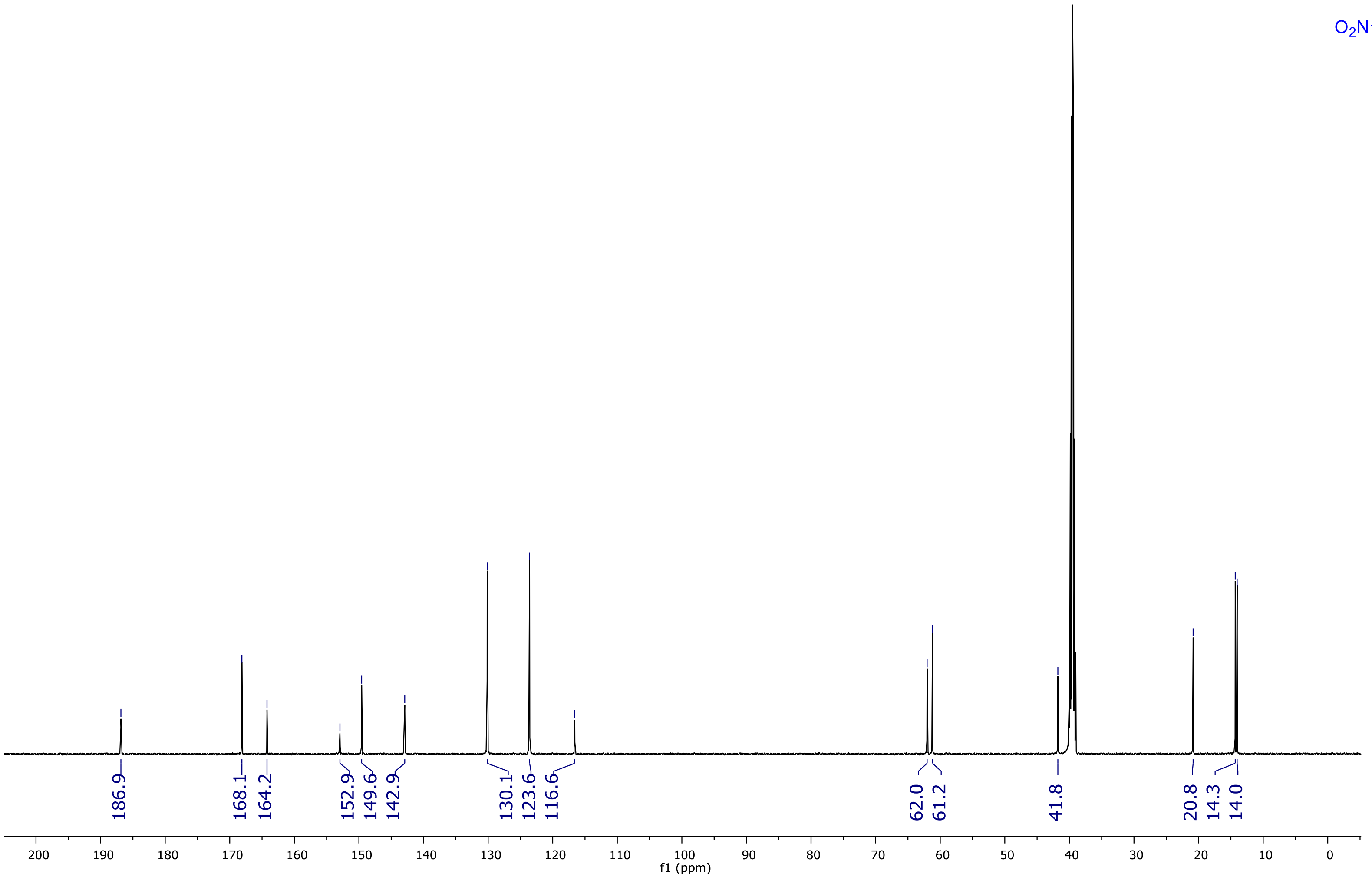

Figure S47. $^{13}C\{^{1}H\}$ NMR Spectrum (125.77 MHz, DMSO-d_6) of compound **3u**

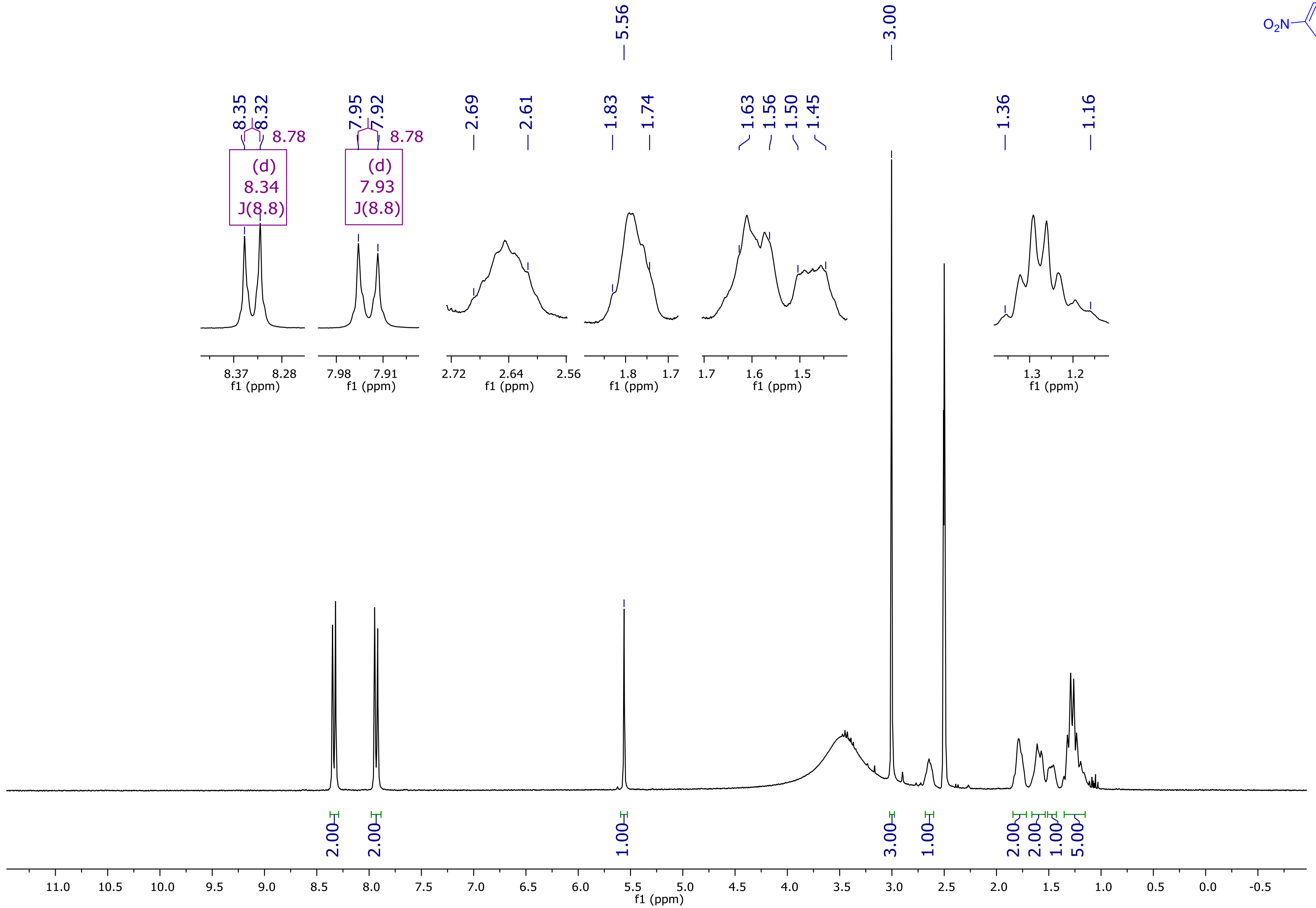

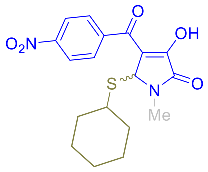

3v

Figure S48. ^{1}H NMR Spectrum (300.06 MHz, DMSO-d$_6$) of compound **3v**

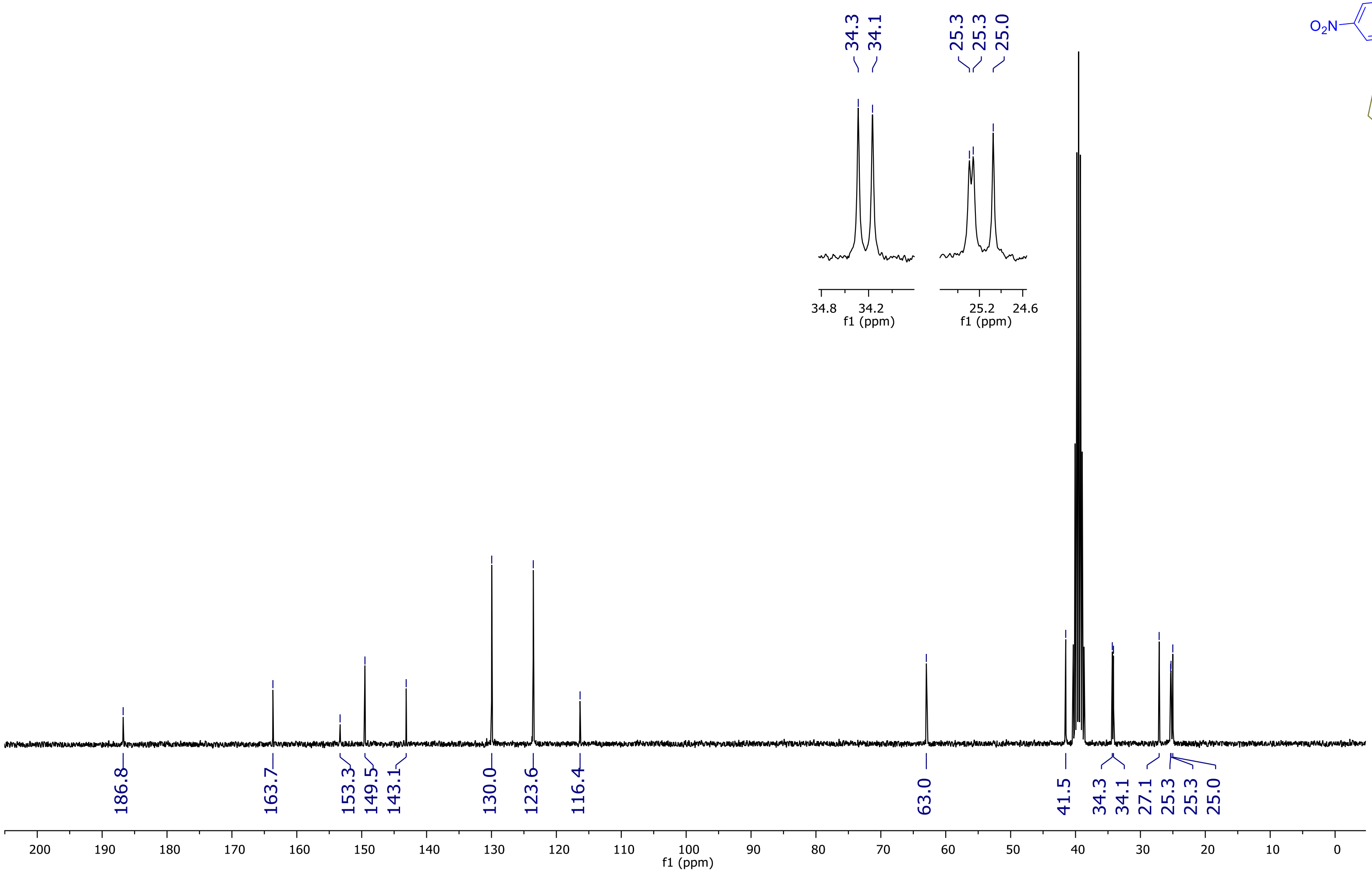

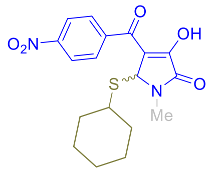

3v

Figure S49. $^{13}C\{^1H\}$ NMR Spectrum (75.46 MHz, DMSO-d_6) of compound **3v**

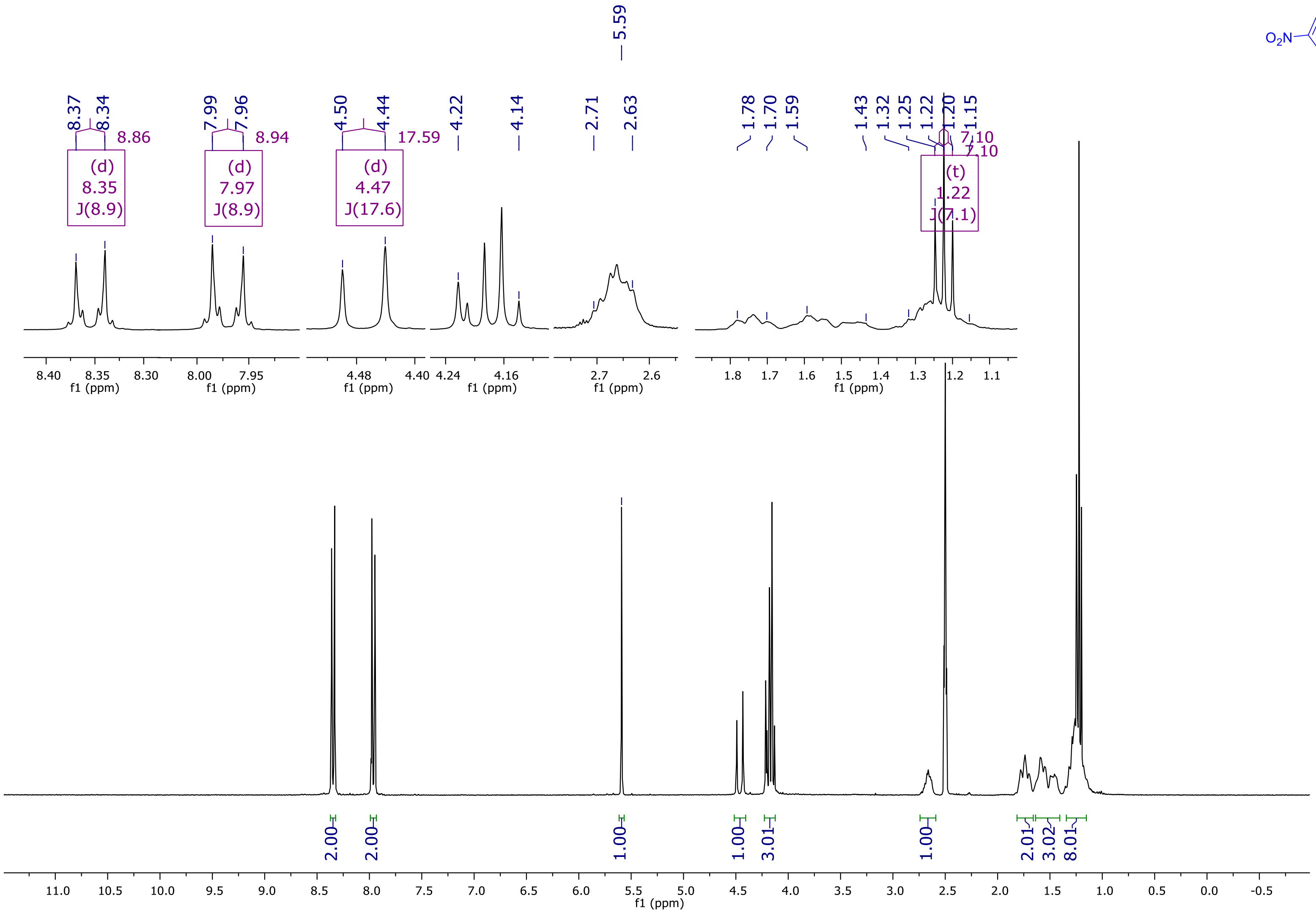

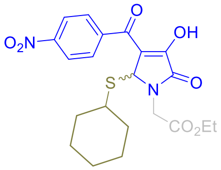

3w

Figure S50. ^{1}H NMR Spectrum (300.06 MHz, DMSO-d$_6$) of compound **3w**

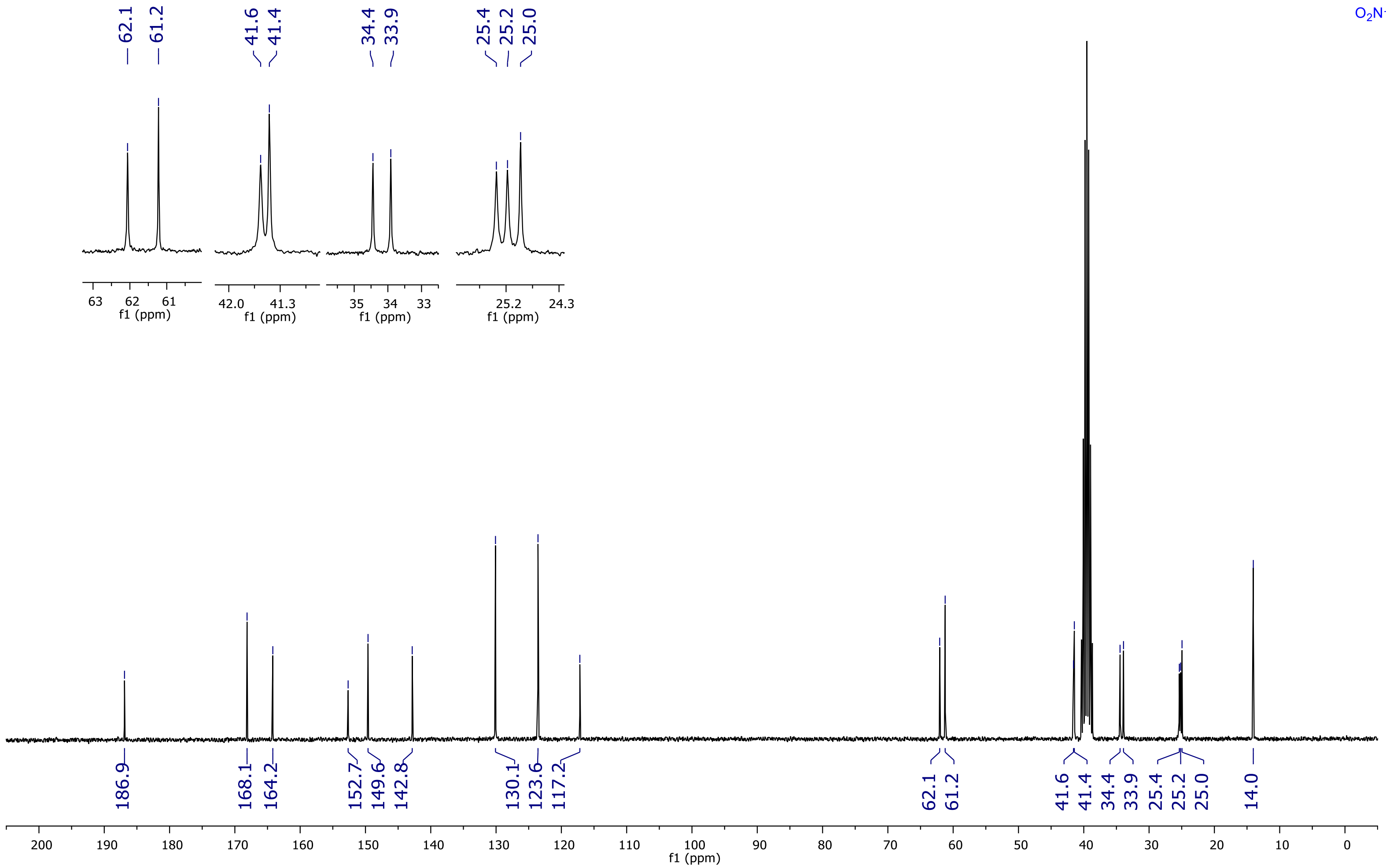

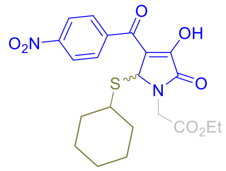

3w

Figure S51. $^{13}C\{^{1}H\}$ NMR Spectrum (75.46 MHz, DMSO-d_6) of compound **3w**

4. References

(1) Perrin, D. D.; Armarego, L. F. In Purification of Laboratory Chemicals, Pergamon Press, New York, 3rd ed, 1996.

(2) (a) Rosa, F. A.; Machado, P.; Rossatto, M.; Vargas, P. S.; Bonacorso, H. G.; Zanatta, N.; Martins, M. A. P. *Synlett* **2007**, 20, 3165-3171. (b) da Silva, M. J. V.; Poletto, J.; Jacomini, A. P.; Pianoski, K. E.; Gonçalves, D. S.; Ribeiro, G. M.; Melo, S. M. S.; Back, D. F.; Moura, S.; Rosa, F. A. *J. Org. Chem.* **2017**, 82, 12590-12602. (c) Poletto, J.; Ribeiro, G. M.; da Silva, M. J. V.; Jacomini, A. P.; Basso, E. A.; Back, D. F.; Moura, S.; Rosa, F. A. *Org. Lett*. **2019**, 21, 6325-6328. (d) Poletto, J.; da Silva, M. J. V.; Pianoski, K. E.; Willig, J. C. M.; Rosa, F. A. *J. Org. Chem*. **2022**, 87, 8544-8550.
